# Factors impacting university students’ quality of life

**DOI:** 10.1371/journal.pone.0329851

**Published:** 2025-08-06

**Authors:** Munir Ibn Mahin, Md. Shamsur Rahman, Sk Mustafizur Rahman, Fahmida Binte Ilias, Md. Mehedi Hasan, Mafia Akter, Abdul Rabbi Mredul

**Affiliations:** Department of Nutrition and Food Engineering, Daffodil International University, Daffodil Smart City, Birulia, Savar, Dhaka, Bangladesh; University of Diyala College of Medicine, IRAQ

## Abstract

**Introduction:**

Quality of life (QoL) is a broad multidimensional concept that incorporates a person’s physical health, psychological well-being, social relations, and environmental factors. Although previous studies have explored QoL among students in health-related disciplines, limited research has assessed QoL in students from diverse academic programs in Bangladesh. This study sought to assess the QoL of University students in Bangladesh and to identify the key factors that determine it.

**Methods:**

In this cross-sectional study, 417 students were recruited from Daffodil International University. Data were obtained using the WHOQoL-BREF, Pittsburgh Sleep Quality Index (PSQI), Rosenberg Self-Esteem Scale (RSES), and a structured questionnaire that included information pertaining to sociodemographic and lifestyle variables.

**Results:**

The highest mean score was observed in the social relationships domain (62.93%), while the environmental health domain had the lowest (58.07%). Students who were physically active and had normal self-esteem reported significantly higher scores across physical and psychological health domains. Poor sleep quality, low self-esteem, and lack of exercise were consistently associated with lower QoL scores, particularly in the physical, psychological, and social domains. Female students also reported significantly lower scores than males in three of the four domains. The most consistent and strongest predictors of quality of life across domains were self-esteem and sleep quality, followed by gender, physical activity, and screen time.

**Conclusion:**

The findings highlight the significant impact of emotional, behavioral, and lifestyle factors on the quality of life of university students. These understandings reinforce the need for campus-based interventions that prioritize mental health support, encourage regular physical exercise, and promote sleep hygiene. Such efforts are essential to enhancing overall student well-being and fostering a healthier, more resilient academic community.

## Introduction

Quality of life (QoL) is a general and dynamic concept that comprises a multitude of physical health, emotional stability, social interaction, environmental conditions, personal beliefs, etc [1]. It is defined differently, but its meaning has been changing over time, and scholars do not agree on the most suitable way to conceptualize and assess QoL across disciplines [2]. To address this challenge, the World Health Organization (WHO) defines QoL as an individual’s perception of his/her position in life within the context of his or her culture, value systems, personal beliefs, personal goals, expectations, concerns, etc [3,4]. This definition reiterates the inherently subjective nature of QoL and thus the importance of considering it in different populations and settings to understand overall well-being and base relevant interventions [5]. The beginning of university life is a new developmental stage for a student since it brings with it new social, intellectual, and personal obligations [6,7]. This is the period during which young adults deal with such things as competitive living environments, academic pressure, lack of parental oversight, changes in lifestyle, and living arrangements [8,9]. That said, students encounter these challenges in tandem with psychosocial transformations of early adulthood, which can be very stressful and, as a result, may lead them to perceive their quality of life [10,11]. Several studies have reported that QoL of university students is generally reported as being in the moderate to low level, and psychological health is shown to be most compromised domain [12–15].

Previous researchers have identified several of these factors as correlated to student QoL [12], including gender [16], financial stability [17], academic satisfaction [18], stress levels [19], sleep quality [20], and body mass index [21]. Higher self-esteem [12], fewer mental health symptoms [15], and healthier lifestyle habits [19] were reported by students who also said that they were more satisfied with QoL in multiple domains [22]. However, much of the current literature has limited its scope to students in health-related programs, including medicine and nursing, and these fields are well-known for their high academic and emotional demands [23]. This leaves the QoL of students from non-Western and developing contexts that are other than the academic discipline and have been understudied.

In Karachi, Pakistan, 62.2% of medical students rated their quality of life as good, though fewer (46.8%) were satisfied with their health [24]. Similar trends appeared in Gujranwala, where 57% described their quality of life as good and 29% as very good [25]. Over time, medical students’ quality of life tends to decline due to increasing academic pressures, leading to 8–11% lower scores compared to non-medical peers [26–28]. Previous studies in international countries have shown that university students often report moderate to low quality of life [29–33]. In Spain, 66.2% of students rated their QoL positively [12]; in Saudi Arabia, the overall mean QoL was 3.99/5 [34]; in Egypt, non-medical students reported better QoL [35]; in Vietnam, dentistry students had lower scores, especially females [36]; in New Zealand, international students reported lower social and environmental QoL [37]; in China, third-year students had the lowest psychological health [38]; and globally, insomnia was widespread, reducing QoL, with wealthier countries less affected [39].

In Bangladesh, where the pace of growth in university education remains quite high, there is a need to understand the quality of life of university students [40]. Research on university students’ quality of life has been growing. However, in Bangladesh, most studies have looked only at students in health-related fields like medicine and nursing [29–33]. These health-related programs are well-known for causing high academic stress [41]. Because of this narrow focus, there is a little evidence about how quality of life varies among students in other subjects and non-health programs in Bangladesh. Past studies have also examined only a few factors at a time [12,20]. Previous studies have rarely looked at how self-esteem, sleep quality, physical activity, and screen time combine to affect well-being of university students in Bangladesh. This study aims to fill that gap.

Therefore, this research aims to assess the factors impacting university students’ quality of life in Bangladesh.

## Methods

### Procedure and participants

This was a cross-sectional study of undergraduate students from four different faculties (Science and Information Technology, Health and Life Sciences, Business and Entrepreneurship and Humanities & Social Sciences) at Daffodil International University (DIU), Dhaka, Bangladesh. The data collection was started from 03/09/2024 and ended on 31/12/2024. Eligible participants were undergraduate students studying full-time in one of four faculties: Science and Information Technology (7 departments), Health and Life Sciences (5 departments), Business and Entrepreneurship (8 departments), and Humanities and Social Sciences (5 departments). Their ages ranged from 18 to 27 years. The inclusion criteria were: (1) being enrolled in any year of undergraduate study during data collection, (2) being able to read and complete the questionnaires in English or Bangla, and (3) giving informed consent to take part. Students who were on a leave of absence or who submitted incomplete or inconsistent questionnaires were excluded from the final analysis. The study used a convenience sampling method, and participation was completely voluntary. The research team explained the objectives of the study in detail in a classroom setting and approached the participants. Questionnaires were distributed to all participants, and each participant provided informed consent. Participation was entirely voluntary, and students were assured that their responses would be anonymous and confidential. Of the 16,905 students enrolled at the university, the sample size was calculated using the Equation 1 [42].


$$Sample\;size,\;n=\frac{N\times Z^{2}\times p(1-p)}{E^{2}\times (N-1)+Z^{2}\times p(1-P)}\;$$ (1)

$$=\frac{16905\times {\left(1.96\right)}^{2}\times 0.5(1-0.5)}{{\left(0.05\right)}^{2}\times (16905-1)+{\left(1.96\right)}^{2}\times 0.5(1-0.5)}$$

$$=\frac{16905\times 3.8416\times 0.25}{0.0025\times 16904+3.8416\times 0.25}$$

$$=\frac{16905\times 3.8416\times 0.25}{0.0025\times 16904+3.8416\times 0.25}$$

$$=\frac{16257.29}{0.0025\times 16904+3.8416\times 0.25}$$

$$=\frac{16257.29}{43.2204}$$

≈376.03=376

Where, N is the population size = 16,905; for 95% confidence, Z score = 1.96; the estimated population proportion, *p* = 50% = 0.5; the margin of error, *E* = 0.05.

**Fig 1:**
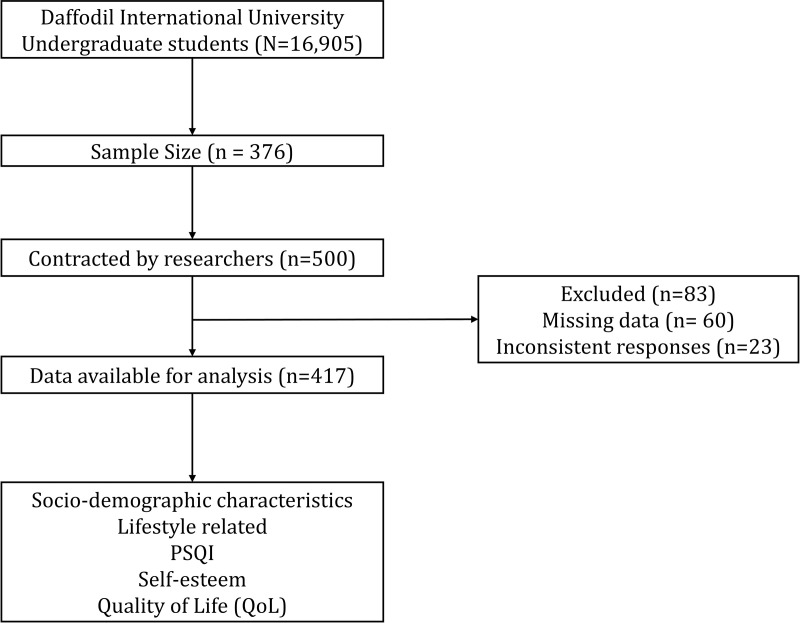
Study flowchart.

The calculation indicates that the requisite minimum sample size to get the acceptable accuracy is roughly 376 students. Since there were total 500 questionnaires data collection completed, from that 83 were excluded due to incomplete or inconsistent responses, making the final sample that consisted of 417 valid responses (**Fig 1**).

The research protocol was reviewed and approved by the Institutional Review Board, Daffodil International University (Ref: FAHSREC/DIU/2024SMIG-12) before the study began. Furthermore, throughout the entire process all national and international ethical standards for research involving human beings followed and applied.

### Data collection instruments

Three internationally validated psychometric tools and sociodemographic items were used to gather data through a structured questionnaire. Demographic, academic, and behavioral data were collected in the form of age, gender, academic year, faculty, residence, body mass index, frequency of physical activity, screen time, pattern of sleep, and religious practice through the first section of the structured questionnaire.

The WHOQoL-BREF, a 26-item instrument designed by the World Health Organization (WHO), was used to assessment of quality of life [43,44]. General perceptions of quality of life and health were measured using the first two items, and the remaining 24 items assessed four separate domains: physical health (7 items), psychological health (6 items), social relationships (3 items), and environmental health (8 items). The items were rated on a 5-point Likert scale. The domain scores were transformed to a 0–100 scale based on WHO guidelines, where a higher scale indicates a better quality of life [44].

To evaluate sleep quality, the Pittsburgh Sleep Quality Index (PSQI) is a 19-item measurement tool derived from seven components of sleep measured over the past month, was used [45]. A Total PSQI score above 5 indicates poor sleep quality. This tool has been widely used and validated in academic populations [46].

The Rosenberg Self-Esteem Scale (RSES) was used to measure self-esteem [47]. A 4-point Likert scale was employed with a total score range of 10 to 40, and 10 items were used to form this 10-item scale. The cut-offs established the pre-contained scores as high (≥ 30), moderate (26 to ≤ 29), and low (≤ 25) [47].

### Data analysis

IBM SPSS Statistical software for Windows version 27.0 was used to analyze the data [48]. All variables were calculated using descriptive statistics, and the mean and standard deviation for continuous data, as well as frequency and percentage for categorical data, were presented. Normality was tested using Shapiro-Wilk test and graphical methods (histograms, Q-Q plots).

Cronbach’s alpha was calculated for each of the WHOQoL-BREF domains to examine internal consistency; values above 0.70 were accepted as acceptable. For comparisons between groups with non-normal distribution, the Mann-Whitney U test and Kruskal-Wallis test was applied. Spearman correlation coefficients were used to bivariate analysis to relate the QoL domains to other continuous variables.

Finally, for each WHOQoL-BREF domain, multiple linear regression analyses were performed to identify the independent predictors of quality of life. Variables with a p < 0.10 threshold were included in the analysis in bivariate terms. All analyses were performed at p < 0.05.

## Results

### Socio-demographic characteristics

Statistical analyses were performed to determine differences in quality of life (QoL) between demographic and lifestyle factors (**Table 1**). The gender effect for three of the four QoL domains was significant with an independent-sample t-test. In the areas of physical health, psychological health, social relationships, and environmental health, males reported higher scores than females, and the differences were statistically significant at p < 0.05, except for the difference in environmental health. The results of one-way ANOVA revealed statistical differences in physical health scores across age groups (p < 0.001), as participants aged 25 and above scored higher (M = 70.24) than those of the younger age groups. Age-related differences were not observed in the psychological, social, or environmental domains. Similarly, comparisons of faculties also presented a marked variation, as the students studying in the Faculty of Science and Information Technology reported the highest physical (M = 67.34) and psychological (M = 64.58) scores, while those in Health and Life Sciences and Business and Entrepreneurship reported lower scores. The highest scores for environmental (M = 63.07) and social relationships (M = 66.67) were given by the Humanities & Social Sciences’ students.

**Table 1:** Participant characteristics and quality of life (QoL) scores across WHOQoL-BREF domains by sociodemographic, behavioral, and lifestyle factors.

	Number(%)/Mean ± SD	Physical Health Domain	Psychological Health Domain	Social Relationships Domain	Environmental Health Domain
		Mean ± SD/ Spearman Correlation Coefficient	Mean ± SD/ Spearman Correlation Coefficient	Mean ± SD/ Spearman Correlation Coefficient	Mean ± SD/Spearman Correlation Coefficient
Gender					
Male	111 (26.6%)	67.3 ± 13.4^b^	64.0 ± 15.9^b^	66.5 ± 14.7^b^	59.9 ± 16.4^a^
Female	306 (73.4%)	61.3 ± 13.6^a^	56.0 ± 15.6^a^	61.6 ± 14.5^a^	57.4 ± 14.9^a^
Age group	1.69 ± 0.54	0.165**	0.101*	0.075	0.096*
<=21	144 (34.5%)	61.81 ± 12.40^a^	56.37 ± 14.42^a^	62.33 ± 15.30^a^	56.55 ± 14.15^a^
22-24	258 (61.9%)	63.08 ± 14.61^ab^	58.98 ± 17.07^a^	63.28 ± 14.58^a^	58.77 ± 15.80^a^
>=25	15 (3.6%)	70.24 ± 11.10^b^	61.67 ± 10.59^a^	62.78 ± 11.30^a^	60.63 ± 17.19^a^
Faculty					
Science and Information Technology	104 (24.9%)	67.34 ± 13.53^b^	64.58 ± 16.08^b^	66.83 ± 14.73^b^	59.56 ± 16.64^ab^
Health and Life Sciences	106 (25.4%)	59.94 ± 12.10^a^	53.46 ± 15.93^a^	58.88 ± 14.65^a^	55.16 ± 13.15^a^
Business and Entrepreneurship	103 (24.7%)	62.69 ± 13.43^ab^	54.05 ± 13.97^a^	59.39 ± 13.39^a^	54.52 ± 13.40^a^
Humanities and Social Sciences	104 (24.9%)	61.68 ± 15.19^b^	60.66 ± 15.52^b^	66.67 ± 14.17^b^	63.07 ± 16.33^b^
Education					
1st year	43 (10.3%)	61.88 ± 12.73^a^	55.72 ± 14.09^a^	64.15 ± 13.05^a^	59.23 ± 11.79^a^
2nd year	223 (53.5%)	61.27 ± 13.82^a^	56.82 ± 16.00^a^	61.32 ± 15.35^a^	56.56 ± 15.41^a^
3rd year	121 (29.0%)	65.53 ± 13.30^a^	61.88 ± 16.15^a^	65.91 ± 13.99^a^	59.89 ± 16.31^a^
Final year	30(7.2%)	65.83 ± 15.94^a^	56.81 ± 16.79^a^	61.11 ± 13.37^a^	60.31 ± 14.22^a^
Place Of Living					
Hostel	162 (38.8%)	63.84 ± 13.10^a^	57.51 ± 15.97^a^	62.29 ± 14.81^a^	57.37 ± 14.01^a^
University Hall	70 (16.8%)	63.42 ± 14.72^a^	59.70 ± 14.96^a^	65.24 ± 13.38^a^	57.90 ± 14.99^a^
Relatives house	4 (1.0%)	64.29 ± 10.51^a^	46.88 ± 17.14^a^	58.33 ± 15.21^a^	55.47 ± 14.29^a^
Home	181(43.4%)	61.82 ± 14.19^a^	58.43 ± 16.49^a^	62.71 ± 15.10^a^	58.82 ± 16.59^a^
Father’s Occupation					
Service	159 (38.1%)	63.34 ± 14.07^a^	58.94 ± 16.22^a^	63.52 ± 15.82^a^	60.16 ± 16.16^a^
Business	175 (42.0%)	62.37 ± 13.81^a^	57.67 ± 15.61^a^	62.10 ± 14.31^a^	57.13 ± 14.93^a^
Professional	45 (10.8%)	63.49 ± 13.66^a^	59.72 ± 17.06^a^	63.70 ± 13.19^a^	57.22 ± 15.68^a^
Other jobs	3 (0.7%)	70.24 ± 20.31^a^	61.11 ± 12.03^a^	66.67 ± 16.67^a^	46.88 ± 10.83^a^
No response	35 (8.4%)	62.14 ± 12.99^a^	55.00 ± 16.57^a^	63.10 ± 13.60^a^	55.36 ± 12.00^a^
Mother’s Working Status					
Does not work	336 (80.6%)	62.50 ± 13.93^a^	58.47 ± 15.58^a^	63.00 ± 14.88^a^	58.33 ± 15.69^a^
Part time	16 (3.8%)	64.96 ± 11.16^a^	62.50 ± 16.60^a^	63.02 ± 13.93^a^	55.66 ± 13.27^a^
Full time	65 (15.6%)	64.45 ± 13.90^a^	55.58 ± 18.03^a^	62.56 ± 14.14^a^	57.31 ± 13.80^a^
Religious Practice (Prayer)					
Regular	169 (40.5%)	63.91 ± 12.62^a^	58.41 ± 15.90^a^	63.12 ± 14.82^a^	59.56 ± 15.21^a^
Irregular	162 (38.8%)	62.85 ± 14.30^a^	57.33 ± 16.19^a^	62.45 ± 14.95^a^	56.33 ± 14.27^a^
Prefer not to say	86 (20.6%)	61.01 ± 15.11^a^	59.30 ± 16.15^a^	63.47 ± 14.13^a^	58.43 ± 17.14^a^
Physical Exercise					
≥5 days/week	24 (5.8%)	69.20 ± 13.92^b^	66.15 ± 15.99^b^	69.79 ± 14.29^b^	62.37 ± 18.74^a^
3-4 days/week	25 (6.0%)	67.14 ± 12.88^ab^	63.67 ± 15.19^ab^	65.33 ± 11.46^ab^	61.25 ± 15.57^a^
1-2 days/week	103 (24.7%)	64.94 ± 11.67^ab^	61.45 ± 12.04^ab^	64.40 ± 13.37^ab^	59.13 ± 14.10^a^
No exercise	265 (63.5%)	61.13 ± 14.38^a^	55.66 ± 16.94^a^	61.51 ± 15.31^a^	56.97 ± 15.35^a^
Screen Time(Devices)	6.43 ± 3.17	−0.022	−0.053	−0.047	−0.105*
<4hrs	105 (25.2%)	63.81 ± 12.57^a^	59.33 ± 14.05^a^	64.21 ± 15.06^a^	59.94 ± 15.63^b^
5-8hrs	249 (59.7%)	62.55 ± 14.56^a^	57.78 ± 17.00^a^	62.55 ± 14.74^a^	58.08 ± 14.94^ab^
>8hrs	63 (15.1%)	62.76 ± 12.95^a^	57.80 ± 15.38^a^	62.30 ± 14.03^a^	54.91 ± 15.93^a^
SELF-ESTEEM	22.55 ± 4.39	−0.372**	−0.557**	−0.300**	−0.333**
Low self-esteem	324 (77.7%)	65.41 ± 12.28^b^	61.93 ± 13.79^b^	64.58 ± 13.40^b^	60.13 ± 15.05^b^
Moderate self-esteem	69 (16.5%)	54.30 ± 14.91^a^	46.26 ± 16.73^a^	56.52 ± 18.52^a^	50.05 ± 13.63^a^
High self-esteem	24 (5.8%)	53.72 ± 16.90^a^	41.67 ± 15.64^a^	59.03 ± 14.10^ab^	53.39 ± 15.33^ab^
Sleep Quality	9.00 ± 3.05	−0.180**	−0.190**	−0.035	−0.135**
Good	174 (41.7%)	66.32 ± 13.33^b^	61.97 ± 16.42^b^	63.51 ± 15.32^a^	61.06 ± 15.11^b^
Poor	243 (58.3%)	60.45 ± 13.68^a^	55.45 ± 15.22^a^	62.52 ± 14.26^a^	55.93 ± 15.12^a^
BMI	22.05 ± 1.43	0.118*	0.114*	0.069	0.029
Underweight	84 (20.1%)	59.61 ± 14.67^a^	54.96 ± 15.62^a^	63.19 ± 14.30^a^	56.81 ± 14.71^a^
Normal	247 (59.2%)	63.50 ± 13.41^a^	59.04 ± 16.00^ab^	62.01 ± 15.22^a^	58.60 ± 15.21^a^
Overweight	66 (15.8%)	63.91 ± 14.11^a^	56.50 ± 16.81^a^	64.52 ± 13.26^a^	56.58 ± 16.18^a^
Obese	20 (4.8%)	65.89 ± 13.13^a^	66.46 ± 12.13^b^	67.92 ± 13.86^a^	61.72 ± 16.25^a^
Waist Circumference	21.94 ± 4.09	−0.002	0.023	0.044	−0.013
<=30 inch	91 (21.8%)	65.89 ± 13.78^b^	58.75 ± 18.11^a^	64.38 ± 15.86^a^	56.97 ± 15.38^a^
>=31 inch	326 (78.2%)	62.06 ± 13.75^a^	58.01 ± 15.44^a^	62.53 ± 14.36^a^	58.38 ± 15.30^a^
Family Income BDT					
<=50,000 BDT	105 (25.2%)	64.32 ± 12.42^a^	58.17 ± 15.84^a^	65.40 ± 12.11^b^	55.77 ± 14.75^a^
>=51,000 BDT	312 (74.8%)	62.42 ± 14.26^a^	58.17 ± 16.13^a^	62.10 ± 15.41^a^	58.84 ± 15.44^a^
Monthly Expenses BDT					
<=20000 BDT	360 (86.3%)	62.28 ± 14.01^a^	57.85 ± 16.25^a^	62.50 ± 14.81^a^	57.98 ± 15.45^a^
>=21000 BDT	57 (13.7%)	66.79 ± 12.02^b^	60.23 ± 14.63^a^	65.64 ± 13.83^a^	58.66 ± 14.53^a^

Values are presented as Mean(M) ± Standard Deviation (SD) or Spearman Correlation Coefficients, where applicable. Superscripts (a, b) indicate statistically significant differences between subgroup means (p < 0.05). Significance: *p < 0.05, **p < 0.001. BDT: Bangladeshi Taka ($1 USD = 122.37 BDT). Faculties: Science and Information Technology (7 departments), Health and Life Sciences (5 departments), Business and Entrepreneurship (8 departments), and Humanities and Social Sciences (5 departments).

All QoL domains, except environmental health, were strongly associated with physical activity levels. There were significant differences in physical (M = 69.20), psychological (M = 66.15), and social relationship (M = 69.79) domains for participants who exercised five or more days per week (M = 69.20, M = 66.15, and M = 69.79, respectively) compared to those who did not exercise (M = 63.05, M = 57.27, and M = 63.10, respectively). This indicates that the higher the perceived quality of life, the more physically active the individual. There was a negative correlation between screen time and environmental health (r = −0.105, p < 0.05), which suggests that individuals with more screen time may perceive the environment of their lives as less healthy.

All four QoL domains were significantly positively associated with self-esteem. However, participants who scored high in self-esteem scored lower in the physical (M = 65.41), psychological (M = 61.93), and social domains (M = 64.58) than those who scored moderately (M = 65.11, M = 62.72, and M = 64.58) or extremely low in self-esteem (M = 65.33, M = 61.14, and M = 64.90), respectively. Finally, students with good sleep quality scored higher in the physical (M = 66.32), psychological (M = 61.97), and environmental (M = 61.06) domains, all of which were statistically significant (p < 0.05). Both BMI and waist circumference had minimal associations with the QoL domain, with small effect sizes. Finally, BMI was very weakly correlated with physical health (r = 0.118, p < 0.001) and psychological health (r = 0.114, p < 0.001). Income and expense did not have a large influence; spenders reported higher dichotomously reported slightly higher physical health scores.

The difference in Quality of Life domain scores between male and female participants was tested using an independent samples t-test. There were 111 (26.6%) male and 306 (73.4%) female participants were there. From the boxplot of the data, it can be judged that there were no outliers. Using the Shapiro-Wilk’s test (p > 0.05) and Levene’s test for equality of variances, scores for each domain were normally distributed, and there was homogeneity of variances.

In the Physical Health Domain (67.3, SD = 13.4 vs 61.3, SD = 13.6), Psychological Health Domain (64.0, SD = 15.9 vs 56.0, SD = 15.6) and Social Relationships Domain (66.5, SD = 14.7 vs 61.6, SD = 14.5), males had higher scores than females (all d.f. (1) p < 0.05). However, in the Environmental Health Domain, the difference was not statistically significant (M = 59.9, SD = 16.4 vs. M = 57.4, SD = 14.9, p > .05). Thus, we can conclude that the first three domains imply that gender is associated with differences in perceived quality of life.

To test whether the groups with different levels of physical exercise had different scores in the quality of life domain, a one-way ANOVA was conducted. The participants were divided into four groups: no exercise (n = 265), 1 to 2 days a week (n = 103), 3–4 days a week (n = 25) and greater than or equal to 5 days a week (n = 24). There were no outliers as per the boxplot, data were normally distributed for each group as per the Shapiro-Wilk test (p > 0.05) and variances were homogeneous as per Levene’s test (p > 0.05).

There were statistically significant differences in physical health, psychological health, and social relationships domain scores between the physical activity groups in the physical health domain (p < 0.001), psychological health domain (p < 0.05), and social relationships domain (p < 0.05). The scores increased consistently as a function of the frequency of exercise in the no exercise group (M = 61.13, SD = 14.38) to the ≥ 5 days group (M = 69.2, SD = 13.92). Finally, Tukey’s post hoc analysis showed differences that were particularly significant between the no-exercise group and subjects that exercised at least 5 days/week. No significant difference was found in the Environmental Health domain (p > .05). Therefore, the first three domains can be rejected by the null hypothesis and take the form of an alternative hypothesis.

### WHOQoL-BREF scores reliability

**Fig 2:**
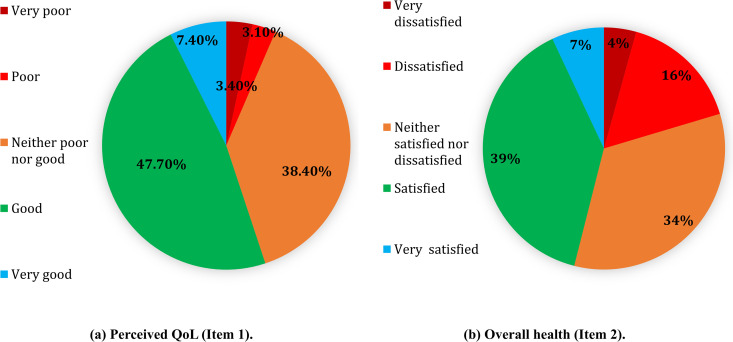
Students’ perception of their quality of life and satisfaction with their health (a, b).

The quality of life (QOL) scores in all domains showed a normal distribution. A total of 47.7% of students positively evaluated their QOL, as well as 39% were satisfied with their overall health, as measured by WHOQOL-BREF items 1 and 2 (**Fig 2**).

Each domain of the WHOQoL-BREF was calculated using descriptive statistics and estimates of internal consistency (**Table 2**). On a 5 point scale from 1 to 5, the mean level of perceived quality of life reported by participants was 3.53 (SD = 0.81), and the mean level of overall health rating was 3.28 (SD = 0.96).

**Table 2:** Participant scores on the WHOQoL-BREF and reliability for each domain.

	mean ± SD	Minimum and Maximum Scores Possible	Minimum and Maximum Scores Obtained	Cronbach’s Alpha
Item 1. Perceived QoL	3.53 ± 0.81	1 - 5	1 - 5	—
Item 2. Overall health	3.28 ± 0.96	1 - 5	1 - 5	—
Physical Health Domain	62.90 ± 13.83	0 - 100	14 - 100	0.714
Psychological Health Domain	58.17 ± 16.04	0 - 100	0 - 100	0.712
Social Relationships Domain	62.93 ± 14.70	0 - 100	0 - 100	0.885
Environmental Health Domain	58.07 ± 15.31	0 - 100	15 - 100	0.772

QoL = Quality of Life; SD = standard deviation. Scores for each domain of the WHOQoL-BREF were transformed to a 0–100 scale. Cronbach’s alpha values indicate internal consistency reliability for each domain >0.70.

The physical health domain had a mean score of 62.90 (SD = 13.83) among the four quality of life domains, with scores ranging from 14 to 100. The mean score for the psychological health domain was less than 58.17 (SD = 16.04), slightly lower, and the social relationships domain had a mean score of 62.93 (SD = 14.70). The mean score (SD = 15.31) for the environmental health domain was 58.07.

Reliability of WHOQoL 26 items scale in terms of internal consistency was measured by Cronbach’s alpha, ranged from moderate to good (**Table 3**). The lowest internal consistency was observed in the psychological health domain (α = 0.712), followed by the physical health domain (α = 0.714). The social relationships domain showed highest reliability (α = 0.885), while the high internal consistency was found in the environmental health domain (α = 0.772). Overall, the WHOQoL-BREF demonstrated acceptable psychometric properties in this sample.

**Table 3:** Bivariate correlation between different domains of the WHOQoL-BREF.

	Perceived QoL	Overall health	Physical Health Domain	Psychological Health Domain	Social Relationships Domain
Perceived QoL					
Overall health	0.411*				
Physical Health Domain	0.383*	0.439*			
Psychological Health Domain	0.506*	0.466*	0.589*		
Social Relationships Domain	0.317*	0.245*	0.376*	0.465*	
Environmental Health Domain	0.458*	0.394*	0.474*	0.502*	0.457*

* Correlation is significant at the 0.001 level (2-tailed).

Spearman correlation coefficients was performed to assess the relationships between the different domains of the WHOQoL-BREF (**Table 3**). The Shapiro-Wilk test (p > 0.05) suggested that the relationships between variables were linear, normally distributed, and free from outliers. The correlations between the perceived quality of life and all other domains were statistically significant and positive. There was a moderately positive correlation between perceived QoL and psychological (r = 0.506, p < 0.001), environmental (r = 0.458, p < 0.001), overall (r = 0.411, p < 0.001), physical (r = 0.383, p < 0.001) and social (r = 0.317, p < 0.001) health.

Positive correlations were obtained for overall health (r = 0.439, p < 0.001), physical (r = 0.466, p < 0.001), psychological (r = 0.394, p < 0.001), environmental health (r = 0.245, p < 0.001), and social relationships (r = 0.245, p < 0.001). The strongest correlations were between psychological and physical health (r = 0.589, p < 0.001) and between psychological and environmental health (r = 0.502, p < 0.001) among the domains themselves. All other domains were positively associated with social relationships, which in turn were positively associated with psychological health (r = 0.465, p < 0.001). All other domains showed a substantially moderate correlation (r = 0.394–0.502), all significant at the 0.001 level with environmental health.

Therefore, it can be concluded that there is an association between higher perceived quality of life and overall health, with better scores on all specific QoL domains, most notably psychological, environmental, and physical health. Given that, the null hypothesis can be rejected and accept the alternative hypothesis that there are statistically significant associations between the Quality of Life(QoL) domains.

### Relationship between socio-demographic variables and QoL domains

To explore the associations between different demographic, behavioral, and psychosocial factors and each of the four domains of the WHOQoL-BREF (i.e., physical health, psychological health, social relationships, and environmental health), a multivariate linear regression analysis was conducted (**Table 4**). The assumptions of homoscedasticity and normality of residuals were considered. The model explained 19.1% (adjusted R² = 17.2%) of the variance in physical health, 27.8% (adjusted R² = 26.0%) in psychological health, 8.28% (adjusted R² = 6.02%) in social relationships, and 10.4% (adjusted R² = 8.16%) in environmental health.

**Table 4:** Multivariate analysis of factors associated with the different QoL domains.

Factors	Physical Health Domain B (95% CI)	Psychological Health Domain B (95% CI)	Social Relationships Domain B (95% CI)	Environmental Health Domain B (95% CI)
Gender (Ref. Male)				
Female	−3.669 (−6.54, −0.7924)*	−5.2170 (−8.371, −2.06)*	−3.6270 (−6.884, −0.370)*	−1.396 (−4.748, 1.96)
Family Income (Ref. <=50,000 BDT)				
>=51,000 BDT	−1.787 (−4.65, 1.0801)	0.0370 (−3.107, 3.18)	−2.8293 (−6.076, 0.418)	2.978 (−0.364, 6.32)
Physical Exercise (Ref. ≥ 5 days/week)				
3-4 days/week	1.308 (−5.84, 8.4535)	2.3309 (−5.504, 10.17)	−3.0383 (−11.131, 5.054)	1.188 (−7.141, 9.52)
1-2 days/week	−2.983 (−8.63, 2.6622)	−3.2949 (−9.484, 2.89)	−4.3206 (−10.714, 2.072)	−2.378 (−8.958, 4.20)
No exercise	−5.293 (−10.62, 0.0316)	−6.8746 (−12.713, −1.04)*	−6.2381 (−12.268, −0.208)*	−3.512 (−9.719, 2.69)
Screen Time (Ref. < 4hrs)				
5-8hrs	0.509 (−2.48, 3.4985)	0.7105 (−2.567, 3.99)	−0.6097 (−3.995, 2.776)	−0.682 (−4.166, 2.80)
>8hrs	1.574 (−2.46, 5.6074)	2.2838 (−2.139, 6.71)	−0.8291 (−5.397, 3.739)	−2.685 (−7.386, 2.02)
Sleep quality (Ref. Good)				
Poor	−4.743 (−7.26, −2.2304)*	−4.7744 (−7.529, −2.02)*	0.0690 (−2.776, 2.914)	−4.138 (−7.066, −1.21)*
Self Esteem (Ref. High self-esteem)				
Low self-esteem	11.576 (6.23, 16.9243)*	19.8159 (13.953, 25.68)*	5.4942 (−0.562, 11.550)	5.536 (−0.697, 11.77)
Moderate self-esteem	1.690 (−4.25, 7.6276)	5.6481 (−0.862, 12.16)	−1.6625 (−8.386, 5.061)	−3.379 (−10.300, 3.54)
R^2^ (R^2^ corrected)	0.191 (0.172)	0.278 (0.260)	0.0828 (0.0602)	0.104 (0.0816)

B: unstandardized regression coefficient, CI: confidence interval, Ref.: reference group, BDT: Bangladeshi Taka(1USD ≈ 122.37BDT). *p < 0.05.

In the physical health domain, poor sleep quality (B = −4.743, 95% CI [−7.26, −2.23], *p* < 0.05) and lack of physical exercise (B = −5.293, 95% CI [−10.62, 0.03], *p* < 0.05) were significantly associated with lower scores. Interestingly, low self-esteem was significantly associated with *higher* physical health scores (B = 11.576, 95% CI [6.23, 16.92], *p* < 0.05), while female students also reported significantly lower scores compared to males (B = −3.669, 95% CI [−6.54, −0.79], *p* < 0.05).

In the psychological health domain, poor sleep quality (B = −4.774, 95% CI [−7.53, −2.02], *p* < 0.05), being female (B = −5.217, 95% CI [−8.37, −2.06], *p* < 0.05), and no physical exercise (B = −6.875, 95% CI [−12.71, −1.04], *p* < 0.05) were significantly associated with lower scores. Conversely, low self-esteem remained a strong positive predictor (B = 19.816, 95% CI [13.95, 25.68], *p* < 0.05), indicating a paradoxical perception of psychological well-being among students with lower self-perceptions.

For the social relationships domain, although the model explained less of the variance, some significant predictors emerged. Female gender (B = −3.627, 95% CI [−6.88, −0.37], *p* < 0.05) and lack of exercise (B = −6.238, 95% CI [−12.27, −0.21], *p* < 0.05) were negatively associated with social relationship scores. Low self-esteem had a positive but non-significant influence (B = 5.494, 95% CI [−0.56, 11.55]).

In the environmental health domain, poor sleep quality remained a significant negative predictor (B = −4.138, 95% CI [−7.07, −1.21], *p* < 0.05). However, none of the other variables, including gender, income, or self-esteem levels, reached statistical significance in this domain, although low self-esteem showed a positive trend (B = 5.536, 95% CI [−0.697, 11.77]).

Overall, sleep quality, gender, physical activity, and self-esteem emerged as the most consistent and influential predictors across multiple quality of life domains. Notably, low self-esteem was paradoxically associated with higher scores in both physical and psychological health domains, a finding that warrants further investigation. Meanwhile, female students and those lacking regular physical activity or experiencing poor sleep quality were consistently at risk for lower perceived quality of life.

## Discussion

In the present study, the quality of life among Bangladeshi university students from a range of academic backgrounds was assessed, and key factors influencing well-being across the four domains of the WHOQoL-BREF were identified. By integrating measures of self-esteem, sleep quality, physical activity, and screen time, a more comprehensive understanding was sought regarding the determinants of student quality of life. In this way, findings were extended beyond what has been reported in prior studies that have mainly focused on health-related disciplines.

The present study sought to evaluate the quality of life (QoL) of Bangladeshi university students and discern the determinants of QoL. The perceived QoL score and overall health rating were reported with a mean of 3.53 (SD = 0.81) and 3.28 (SD = 0.96), respectively on a 5 point scale. The findings from these studies are in line with global studies that found moderate perceived levels of well-being and health status among university students [49–51]. Among the four domains of the WHOQoL-BREF, the mean score in the social relationships domain was the highest (M = 62.93, SD = 14, 70), whereas the psychological health domain had the lowest score (M = 58.17, SD = 16, 04). These mirrored results have been reported in other countries, where psychological well-being was sometimes a weak area for university students [50,52]. Across studies, these differences in QoL domain scores may represent the contextual influence of students’ patterns of experience and perception, such as educational system [53], cultural expectations [54], and socioeconomic status [51].

Consistent with prior studies, gender was a significant predictor across three domains. Female students reported significantly lower scores in physical (B = −3.669), psychological (B = −5.217), and social relationships (B = −3.627) domains compared to their male counterparts, indicating potential vulnerabilities in female student well-being. Female students often face higher levels of anxiety, depression, academic stress, and financial pressure, all of which take a toll on their quality of life [55,56]. Challenges like poor sleep, limited time for self-care, PMS, and the burden of societal expectations make it harder for them to maintain good physical and mental health [57]. Strong social support and better access to resources are essential to help them cope and thrive during their studies [58]. Self-esteem emerged as the strongest positive predictor of QoL in both the physical (B = 11.576) and psychological (B = 19.816) health domains. Self-esteem strongly shapes students’ quality of life, influencing both physical and psychological health. Higher self-esteem is linked to better health outcomes and lower depression and anxiety [59,60]. Over time, maintaining strong self-esteem can protect well-being [61,62]. Sleep quality was a consistent and significant negative predictor across the physical (B = −4.743), psychological (B = −4.774), and environmental (B = −4.138) domains. Poor sleep is common among students and takes a real toll on both physical and mental health. Those with poor sleep report more fatigue, worse physical health, and higher levels of stress, anxiety, and depression [63–65]. Regular exercise can help improve sleep and reduce these negative effects [66,67]. Environmental factors like dorm living, late-night screen use, and irregular sleep schedules also worsen sleep quality and overall well-being [68,69]. Physical exercise was significantly associated with higher QoL, particularly in the psychological and social domains. Students who did not exercise reported substantially lower scores in psychological (B = −6.875) and social (B = −6.238) domains compared to those who exercised ≥5 days per week. Regular physical activity is linked to better mental health, with studies showing it helps reduce depression and anxiety and builds psychological resilience [70]. It also boosts overall well-being and life satisfaction across age groups [71]. Exercise can improve social connections and foster a sense of community, its social benefits may vary depending on factors like personal networks and demographics [72,73].

Taken together, the findings can be seen as providing new evidence that expands the current literature on students’ Quality of Life(QoL) in Bangladesh. Unlike earlier research that has been limited to specific faculties or single predictors, the combined influence of multiple factors across various academic programs has been showed. Through this broader approach, novel understandings have been offered into how interventions targeting self-esteem, sleep quality, and physical activity might be adapted across different disciplines.

### Limitation

Some limitations of these findings have been identified, as these findings should help enhance the understanding of QoL for Bangladeshi university students. However, the cross-sectional design does not allow for causal inference and does not show temporal changes in QoL. The data were drawn from a single academic institution, which limits the generalizability of the results. Self-report measures may be prone to recall or social desirability biases, and add to this, other sources of artifacts such as social desirability. Future research should be designed longitudinally with additional variables that are more nuanced, such as dietary habits, academic satisfaction, financial stress, and social support systems. Despite these limitations, this study demonstrates how student’s well-being is a multidimensional concept and how factors such as self-esteem, physical activity, and sleep quality are modifiable and could be used to improve health promotion. Interventions that promote a healthy lifestyle and mental health and support a balanced academic environment must be integrated into universities and policymakers’ considerations to improve students’ quality of life.

## Conclusion

This study assessed quality of life among Bangladeshi university students from diverse disciplines, finding overall moderate QoL with the highest scores in social relationships and the lowest in environmental health. Poor sleep, lack of exercise, and being female were linked to lower QoL, while self-esteem was the strongest predictor, despite some unexpected associations. By examining multiple factors beyond health fields, this research highlights the need for targeted interventions to improve sleep, physical activity, and self-esteem among university students. These findings show why universities, mental health professionals, and policymakers need to take action. Helping students improve sleep, stay active, and build self-esteem can make a real difference in their quality of life. Efforts like awareness campaigns, faculty training, wellness programs, and better support services are key to creating a more supportive environment. By tackling the behavioral and psychological challenges students face, universities can help build a healthier, more resilient generation. This study also highlights the need for ongoing research and proactive health initiatives to keep improving student well-being in Bangladesh. Future studies should explore causal links and test strategies to enhance student well-being.

## Supporting information

S1 File Questionnaires in Bangla. (PDF)

S2 File Questionnaires in English. (PDF)

S3 File Datasets. (PDF)
