# Supplementary material for: Factors impacting university students’ quality of life
Source: PLoS One. 2025 Aug 6;20(8):e0329851. doi: 10.1371/journal.pone.0329851 (PMC12327633; doi:10.1371/journal.pone.0329851)
Supplement: S1 File — (PDF) [file pone.0329851.s001.pdf]

অফিসিয়াল ব্যবহারের জন্য: তারিখ: _ _ / _ _ / 2024 | উত্তরদাতা নং: _ _ _ | ডেটা এন্ট্রি: ☐

সম্মতি

আপনাকে একটি গবেষণা অধ্যয়নে অংশগ্রহণ করতে বলা হচ্ছে যা আপনার জীবনমানের সাথে সম্পর্কিত। এই অধ্যয়নের পদ্ধতি বা কার্যক্রম সম্পূর্ণ ঝুকিমুক্ত। এই গবেষণায় আপনার অংশগ্রহণের জন্য কোনো খরচ নেই বা আপনি কোনো প্রণোদনা পাবেন না। আপনি সম্মত হলে, আপনাকে কয়েকটি ব্যক্তিগত প্রশ্ন জিজ্ঞাসা করা হবে। আপনি যেকোন প্রশ্নের উত্তর দিতে অস্বীকার করতে পারেন বা আপনার ইচ্ছামত যেকোনো সময় চলে যেতে পারেন। এই গবেষণায় প্রাপ্ত সমস্ত তথ্য কঠোরভাবে গোপনীয় এবং আপনার পরিচয় প্রকাশ করা হবে না। শুধুমাত্র অধ্যয়ন সম্পর্কিত কর্মীদের তথ্য দেখার অনুমতি দেওয়া হবে। আপনার সহযোগিতা অত্যন্ত কাম্য।

স্বাক্ষর: ________________________

শুরু

ডেমোগ্রাফিক তথ্য

**01.**	**নাম:** ____________________	
**02.**	**লিঙ্গ:** ☐পুরুষ = 1 ☐মহিলা = 2	
**03.**	**বয়স (বছর):** _____________________	**ওজন (কেজি):** _____________________
**04.**	**উচ্চতা (ফুট ইঞ্চি):** _____________________	**কোমরের পরিধি:** ______________
**05.**	**বিশ্ববিদ্যালয়ের ধরন:** ☐প্রাইভেট=1 ☐পাবলিক=2	**বিশ্ববিদ্যালয়ের নাম:**_________________________________
**06.**	**বিভাগের নাম:**______________________	
**07.**	**শিক্ষা:** ☐১ম বছর=১ ☐২য় বছর=২ ☐৩য় বছর=৩ ☐শেষ বছর=৪	
**08.**	**থাকার জায়গা:** ☐হোস্টেল = 1 ☐বিশ্ববিদ্যালয়ের হল = 2 ☐আত্মীয়ের বাড়ি = 3 ☐বাড়ি = 4	
**09.**	**পিতার পেশা:**<br>☐ চাকুরি=1 ☐ব্যবসা=2 ☐পেশাজীবী(ডাক্তার, শিক্ষক, প্রকৌশলী)=3 ☐মেনিয়াল চাকরি (ড্রাইভার/প্লাম্বার/পেইন্টার)=4 ☐ফ্রিল্যান্সিং =5 ☐বলতে ইচ্ছুক নই=6	
**10.**	**মায়ের কাজের অবস্থা:**<br>☐কাজ করে না=1 ☐পার্ট টাইম চাকুরি=2 ☐ফুল টাইম চাকুরি =3	
**11.**	**পারিবারিক আয় (প্রায়)** :_________________BDT	
**12.**	**মাসিক খরচ (প্রায়):** _______________BDT	
**13.**	**স্ক্রীন টাইম (ফোন/মোবাইল ডিভাইস):** ________________________ঘন্টা	
**14.**	**ধর্মীয় অনুশীলন (প্রার্থনা):** ☐নিয়মিত ☐অনিয়মিত ☐না বলা পছন্দ	
**15.**	**ঘুমানোর সময়:** ☐ <4 hrs /d=1 ☐ 4–6 hrs /d=2 ☐ 6–8 hrs /d=3 ☐ >8 hrs /d=4	
**16.**	**শারীরিক ব্যায়াম:** ☐≥5 দিন/সপ্তাহ=1 ☐3-4 দিন/সপ্তাহ=2 ☐1-2 দিন/সপ্তাহ=3 ☐ব্যায়াম নেই=4	

প্রথম অংশ

		খুব খারাপ	খারাপ	খারাপও নয় বা ভালোও নয়	ভাল	খুব ভালো
1 (G1)	আপনার জীবনের যাত্রার মান কেমন?	1	2	3	4	5

		খুব অসন্তুষ্ট	অসন্তুষ্ট	সন্তুষ্ট নয় বা অসন্তুষ্ট নয়	সন্তুষ্ট	খুব সন্তুষ্ট
2 (G4)	আপনার স্বাস্থ্য নিয়ে কি আপনি সন্তুষ্ট?	1	2	3	4	5

		একদমই না	কম	মোটামুটি	বেশি	খুব বেশি
3 (F1.4)	শারীরিক ব্যথার জন্য আপনি কি পরিমাণ কাজ থেকে বিরত ছিলেন?	1	2	3	4	5
4 (F11.3)	আপনার দৈনন্দিন কার্যক্রম ঠিক রাখতে চিকিৎসা কতটুকু প্রয়োজন ?	1	2	3	4	5
5 (F4.1)	আপনি জীবনকে কতটা উপভোগ করেন?	1	2	3	4	5
6 (F24.2)	জীবনকে আপনার কতটা অর্থপূর্ণ মনে হয়?	1	2	3	4	5

		একদমই না	কম	মোটামুটি	বেশি	খুব বেশি
7 (F5.3)	আপনি কাজে কতটা ভালোভাবে মনোনিবেশ করতে পারবেন?	1	2	3	4	5
8 (F16.1)	আপনি দৈনন্দিন জীবনে কতটা নিরাপদ অনুভব করেন?	1	2	3	4	5
9 (F22.1)	আপনার ভৌত পরিবেশ কতটা স্বাস্থ্যকর?	1	2	3	4	5

		একদমই না	একটু	মোটামুটি	অধিকাংশ ক্ষেত্রে	সম্পূর্ণরূপে
10 (F2.1)	আপনার কি প্রতিদিন কাজ করার মত শক্তি আছে?	1	2	3	4	5
11 (F7.1)	আপনি কি আপনার শারীরিক গড়ন নিয়ে সন্তুষ্ট?	1	2	3	4	5
12 (F18.1)	আপনার কি প্রয়োজন মেটাতে যথেষ্ট টাকা আছে?	1	2	3	4	5
13 (F20.1)	আপনি কি দৈনন্দিন জীবন-যাপনের জন্য প্রয়োজনীয় তথ্য পান?	1	2	3	4	5
14 (F21.1)	আপনি অবসর/বিনোদনের জন্য কতটা সুযোগ পান?	1	2	3	4	5

		খুব খারাপ	খারাপ	খারাপও নয় বা ভালোও নয়	ভাল	খুব ভালো
15 (F9.1))	আপনি কতটা ভালভাবে চলাফেরা করতে পারেন ?	1	2	3	4	5

		খুব অসন্তুষ্ট	অসন্তুষ্ট	সন্তুষ্ট নয় বা অসন্তুষ্ট নয়	সন্তুষ্ট	খুব সন্তুষ্ট
16 (F3.3)	আপনি আপনার ঘুম নিয়ে কতটা সন্তুষ্ট?	1	2	3	4	5
17 (F10.3)	দৈনন্দিন কাজ করার ক্ষমতা নিয়ে আপনি কতটুকু সন্তুষ্ট ?	1	2	3	4	5
18 (F12.4)	আপনার কাজ করার ক্ষমতা/দক্ষতা নিয়ে আপনি কতটুকু সন্তুষ্ট?	1	2	3	4	5
19 (F6.3)	নিজেকে নিয়ে আপনি কতটা সন্তুষ্ট?	1	2	3	4	5
20 (F13.3)	অন্যদের সাথে আপনার ব্যক্তিগত সম্পর্ক নিয়ে আপনি কতটা সন্তুষ্ট?	1	2	3	4	5
21 (F15.3)	আপনার যৌন জীবন নিয়ে আপনি কতটা সন্তুষ্ট?	1	2	3	4	5
22 (F14.4)	বন্ধুদের কাছ থেকে পাওয়া সাহায্যে আপনি কতটুকু সন্তুষ্ট?	1	2	3	4	5
23 (F17.3)	আপনি আপনার বাসস্তানের অবস্থা নিয়ে কতটা সন্তুষ্ট?	1	2	3	4	5
24 (F19.3)	আপনি যে স্বাস্থ্য সেবা পান তাতে কি সন্তুষ্ট?	1	2	3	4	5
25 (F23.3)	আপনি যাতায়াত ব্যবস্থা নিয়ে কতটা সন্তুষ্ট?	1	2	3	4	5

		কখনই না	কদাচিৎ	মাঝে মাঝে	প্রায়ই	সর্বদা
26 (F8.1)	আপনার হতাশা, উদ্বেগ, বিষণ্ণতা এই সব নেতিবাচক অনুভুতি কত ঘন ঘন হয়?	1	2	3	4	5

দ্বিতীয় অংশ

1.গত মাসে, আপনি সাধারণত রাতে কখন ঘুমাতে গিয়েছেন? — স্বাভাবিক ঘুমাতে যাবার সময়:________________________

2.গত মাসে, প্রতি রাতে সাধারণত কতক্ষণ (মিনিটের মধ্যে) আপনি ঘুমিয়ে পড়েন ? — মিনিটের সংখ্যা:________________________

3.গত মাসে, আপনি সাধারণত কখন সকালে ঘুম থেকে উঠেছেন? — স্বাভাবিক ঘুম থেকে ওঠার সময়: ________________________

4.গত মাসে, আপনি প্রকৃত অর্থে কত ঘন্টা রাতে ঘুমিয়েছিলেন? — প্রতি রাতে ঘুমের পরিমাণ(ঘণ্টা): ________________________

5 গত মাসে, আপনার ঘুমাতে সমস্যা হয়েছে কারণ আপনি...				
	একবারো নয় (0)	সপ্তাহে একবারেরও কম (1)	সপ্তাহে একবার বা দুবার (2)	সপ্তাহে তিন বা তার বেশি বার (3)
a. ৩০ মিনিটের মধ্যে ঘুমাতে পারেন নি	**0**	**1**	**2**	**3**
b. মাঝরাতে বা ভোরবেলা ঘুম থেকে উঠতে হয়েছে	**0**	**1**	**2**	**3**
c. বাথরুম ব্যবহার করতে উঠতে হয়েছে	**0**	**1**	**2**	**3**
d. আরামে শ্বাস নিতে পারেন নি	**0**	**1**	**2**	**3**
e. কাশি বা জোরে নাক ডাকা	**0**	**1**	**2**	**3**
f. খুব ঠান্ডা লেগেছিলো	**0**	**1**	**2**	**3**
g. খুব গরম লেগেছিলো	**0**	**1**	**2**	**3**
h. খারাপ স্বপ্ন ছিল	**0**	**1**	**2**	**3**
i ব্যথা ছিল	**0**	**1**	**2**	**3**

j অন্য কারণ(গুলি), দয়া করে বর্ণনা করুন ____

	একবারো নয় (0)	সপ্তাহে একবারেরও কম (1)	সপ্তাহে একবার বা দুবার (2)	সপ্তাহে তিন বা তার বেশি বার (3)
গত মাসে কতবার এই কারণে আপনার ঘুমাতে সমস্যা হয়েছে?	**0**	**1**	**2**	**3**

	খুব ভাল (0)	মোটামুটি ভাল (1)	মোটামুটি খারাপ (2)	খুব খারাপ (3)
6. গত মাসে, আপনি সামগ্রিকভাবে আপনার ঘুমের গুণমানকে কীভাবে মূল্যায়ন করবেন?	**0**	**1**	**2**	**3**
	একবারো নয় (0)	একবারেরও কম এক সপ্তাহ (1)	একবার অথবা দুইবার এক সপ্তাহ (2)	তিন বা তার বেশি সপ্তাহে বার (3)
7.গত মাসে, আপনি কত ঘন ঘন ঔষধ (নির্ধারিত বা "কাউন্টারে") খেয়েছেন যাতে আপনাকে ঘুমাতে সাহায্য করে?	**0**	**1**	**2**	**3**
	একবারো নয় (0)	একবারেরও কম এক সপ্তাহ (1)	একবার অথবা দুইবার এক সপ্তাহ (2)	তিন বা তার বেশি সপ্তাহে বার (3)
8.গত মাসে, গাড়ি চালানোর সময়, খাবার খাওয়ার সময় বা সামাজিক কার্যকলাপে জড়িত থাকার সময় আপনি কতবার জেগে থাকতে সমস্যায় পড়েছেন?	**0**	**1**	**2**	**3**

	সমস্যা নেই মোটেও (0)	শুধুমাত্র একটি খুব সামান্য সমস্যা (1)	কিছুটা একটি সমস্যা (2)	একটি খুব বড় সমস্যা (3)
9.গত মাসে, কাজকরার জন্য উৎসাহ বজায় রাখতে আপনার জন্য কতটা সমস্যা হয়েছে?	**0**	**1**	**2**	**3**
	বিছানার সঙ্গী অথবা রুম মেট নেই(0)	পার্টনার/রুম অন্য মধ্যে সঙ্গী রুম(1)	একই অংশীদার রুম, কিন্তু না একই বিছানা(2)	একই বিছানায় সঙ্গী (3)
10. আপনার কি একজন বিছানা সঙ্গী বা রুম মেট আছে?	**0**	**1**	**2**	**3**

11. আপনি গত মাসে কতবার...				
	একবারো নয় (0)	সপ্তাহে একবার(1)	সপ্তাহে একবার অথবা দুইবার(2)	সপ্তাহে তিন বা তার বেশি বার (3)
a.জোরে নাক ডাকা	**0**	**1**	**2**	**3**
b.ঘুমানোর সময় শ্বাসের মধ্যে দীর্ঘ বিরতি	**0**	**1**	**2**	**3**
c.ঘুমের সময় পা কাঁপছে বা ঝাঁকুনি দিচ্ছে	**0**	**1**	**2**	**3**
d. ঘুমের সময় বিভ্রান্তি বা বিভ্রান্তির পর্ব	**0**	**1**	**2**	**3**
e. আপনি ঘুমানোর সময় অন্যান্য অস্থিরতা;অনুগ্রহ করে বর্ণনা করুন				

<u>তৃতীয় অংশ</u>

	**দৃঢ়ভাবে একমত**	**একমত**	**অসম্মত**	**দৃঢ়ভাবে অসম্মত**
1. সামগ্রিকভাবে, আমি নিজেকে নিয়ে সন্তুষ্ট।	☐	☐	☐	☐
2. মাঝে মাঝে আমি মনে করি আমি মোটেও ভালো নেই।	☐	☐	☐	☐
3. আমি অনুভব করি যে আমার অনেকগুলি ভাল গুণ রয়েছে।	☐	☐	☐	☐
4. আমি অন্যান্য সবার মতো কাজ করতে সক্ষম।	☐	☐	☐	☐
5. আমি অনুভব করি যে আমার গর্ব করার মতো অনেক কিছুই নেই।	☐	☐	☐	☐
6. আমি মাঝে মাঝে নিজেকে অকেজো মনে করি।	☐	☐	☐	☐
7. আমি অনুভব করি যে আমি একজন মূল্যবান ব্যক্তি, অন্তত অন্যদের সাথে সমান সমতলে।	☐	☐	☐	☐
8. আমি যদি নিজেকে আরও সম্মান করতে পারতাম।	☐	☐	☐	☐
9. সব মিলিয়ে, আমি অনুভব করি যে আমি একজন ব্যর্থ।	☐	☐	☐	☐
10. আমি নিজের প্রতি ইতিবাচক মনোভাব গ্রহণ করি।	☐	☐	☐	☐
