# Supplementary material for: Factors impacting university students’ quality of life
Source: PLoS One. 2025 Aug 6;20(8):e0329851. doi: 10.1371/journal.pone.0329851 (PMC12327633; doi:10.1371/journal.pone.0329851)
Supplement: S2 File — (PDF) [file pone.0329851.s002.pdf]

FOR OFFICIAL USE: Date: _ _ / _ _ / 202_ | Respondent No: _ _ _ | Data Entry: ☐ OK

Consent

You are being asked to participate in a research study that deals with your Quality of Life(QoL). The procedures or activities in this study are completely harmless. There are no costs for your participation in this study neither you will get any payment. If you agree, you will be asked a few personal questions. You can refuse to answer any question or may leave any time you like. All information obtained in this study is strictly confidential and your identity will not be disclosed. Only study related personnel will be allowed to see the information. Your cooperation is highly appreciated.

Signature:________________________

Instructions

This assessment asks how you feel about your quality of life, health, or other areas of your life. Please answer all the questions. If you are unsure about which response to give to a question, please choose the one that appears most appropriate. This can often be your first response.

Please keep in mind your standards, hopes, pleasures and concerns. We ask that you think about your life in the last two weeks. For example, thinking about the last two weeks, a question might ask:

		Not at all	Not much	Moderately	A great deal	Completely
	Do you get the kind of support from others that you need?	1	2	3	4	5

You should circle the number that best fits how much support you got from others over the last two weeks.

<u>Part Zero</u>

DEMOGRAPHIC INFORMATION

01. **Name:** ____________________
02. **Gender:** ☐Male=1 ☐Female=2
03. **Age(yrs):** _____________________ **Weight(kg):** _____________________
04. **Height (ft in):** _____________________ **Waist Circumference:** ______________
05. **Type of University:** ☐Private=1 ☐Public=2 **University name:**______________________________
06. **Department name:**______________________
07. **Education:** ☐1st year=1 ☐2nd year=2 ☐3rd year=3 ☐Final year=4
08. **Place of living:** ☐Hostel=1 ☐University's Hall=2 ☐Relative's House=3 ☐Home=4
09. **Father's occupation:**

    ☐Service=1 ☐Business=2 ☐Professional=3 ☐Menial jobs (driver/plumber/painter)=4

    ☐Self-employed=5 ☐No response=6
10. **Mother's working status:**

    ☐Does not work=1 ☐Part time=2 ☐Full time=3
11. **Family income(approx.):**_________________BDT
12. **Monthly Expenses(approx.):**_______________BDT
13. **Screen time (phone/mobile devices):**________________________hours
14. **Religious practice(Prayer):** ☐Regular ☐Irregular ☐Prefer not to say
15. **Sleeping time:** ☐<4 hrs/d=1 ☐4–6 hrs/d=2 ☐6–8 hrs/d=3 ☐>8 hrs/d=4
16. **Physical exercise:** ☐≥5 days/week=1 ☐3–4 days/week=2 ☐1–2 days/week=3 ☐No exercise=4

PART ONE

THE WHOQOL-BREF

		Very poor	Poor	Neither poor nor good	Good	Very good
1 (G1)	How would you rate your quality of life?	1	2	3	4	5

		Very dissatisfied	Dissatisfied	Neither satisfied nor dissatisfied	Satisfied	Very satisfied
2 (G4)	How satisfied are you with your health?	1	2	3	4	5

The following questions ask about how much you have experienced certain things in the last two weeks.

		Not at all	A little	A moderate amount	Very much	An extreme amount
3 (F1.4)	To what extent do you feel that (physical) pain prevents you from doing what you need to do?	1	2	3	4	5
4 (F11.3)	How much do you need any medical treatment to function in your daily life?	1	2	3	4	5
5 (F4.1)	How much do you enjoy life?	1	2	3	4	5
6 (F24.2)	To what extent do you feel your life to be meaningful?	1	2	3	4	5

		Not at all	A little	A moderate amount	Very much	Extremely
7 (F5.3)	How well are you able to concentrate?	1	2	3	4	5
8 (F16.1)	How safe do you feel in your daily life?	1	2	3	4	5
9 (F22.1)	How healthy is your physical environment?	1	2	3	4	5

The following questions ask about how completely you experience or were able to do certain things in the last two weeks.

		Not at all	A little	Moderately	Mostly	Completely
10 (F2.1)	Do you have enough energy for everyday life?	1	2	3	4	5
11 (F7.1)	Are you able to accept your bodily appearance?	1	2	3	4	5
12 (F18.1)	Have you enough money to meet your needs?	1	2	3	4	5
13 (F20.1)	How available to you is the information that you need in your day-to-day life?					
14 (F21.1)	To what extent do you have the opportunity for leisure activities?					

		Very poor	Poor	Neither poor nor good	Good	Very good
15 (F9.1))	How well are you able to get around?	1	2	3	4	5

The following questions ask you to say how good or satisfied you have felt about various aspects of your life over the last two weeks.

		Very dissatisfied	Dissatisfied	Neither satisfied nor dissatisfied	Satisfied	Very satisfied
16 (F3.3)	How satisfied are you with your sleep?	1	2	3	4	5
17 (F10.3)	How satisfied are you with your ability to perform your daily living activities?	1	2	3	4	5
18 (F12.4)	How satisfied are you with your capacity for work?	1	2	3	4	5
19 (F6.3)	How satisfied are you with yourself?	1	2	3	4	5
20 (F13.3)	How satisfied are you with your personal relationships?	1	2	3	4	5
21 (F15.3)	How satisfied are you with your sex life?	1	2	3	4	5
22 (F14.4)	How satisfied are you with the support you get from your friends?	1	2	3	4	5
23 (F17.3)	How satisfied are you with the conditions of your living place?	1	2	3	4	5
24 (F19.3)	How satisfied are you with your access to health services?	1	2	3	4	5
25 (F23.3)	How satisfied are you with your transport?	1	2	3	4	5

The following question refers to how often you have felt or experienced certain things in the last two weeks.

		Never	Seldom	Quite often	Very often	Always
26 (F8.1)	How often do you have negative feelings such as blue mood, despair, anxiety, depression?	1	2	3	4	5

Did someone help you to fill out this form?...

How long did it take to fill this form out?..

Do you have any comments about the assessment?

..

..

PART TWO

Pittsburgh Sleep Quality Index

The following questions relate to your usual sleep habits during the past month only. Your answers should indicate the most accurate reply for the majority of days and nights in the past month.

1.During the past month, when have you usually gone to bed at night?	Usual bed time	___________________________
2.During the past month, how long (in minutes) has it usually taken you to fall asleep each night?	Number of minutes	___________________________
3.During the past month, when have you usually gotten up in the morning?	Usual getting up time	___________________________
4.During the past month, how many hours of actual sleep did you get at night? (This may be different than the number of hours you spend in bed.)	Hours of sleep per night	___________________________

For each of the remaining questions, check the one best response. Please answer all questions

**5 During the past month, how often have you had trouble sleeping because you...**				
	Not during the past month (0)	Less than once a week (1)	Once or twice a week (2)	Three or more times a week (3)
a. cannot get to sleep within 30 minutes	☐	☐	☐	☐
b. wake up in the middle of the night or early morning	☐	☐	☐	☐
c. have to get up to use the bathroom	☐	☐	☐	☐
d. cannot breathe comfortably	☐	☐	☐	☐
e. cough or snore loudly	☐	☐	☐	☐
f. feel too cold	☐	☐	☐	☐
g. feel too hot	☐	☐	☐	☐
h. have bad dreams	☐	☐	☐	☐
i. have pain	☐	☐	☐	☐

j. other reason(s), please describe ____

	Not during the past month (0)	Less than once a week (1)	Once or twice a week (2)	Three or more times a week (3)
How often during the past month have you had trouble sleeping because of this?	☐	☐	☐	☐

	**Very good (0)**	**Fairly good (1)**	**Fairly bad (2)**	**Very bad (3)**
6. During the past month, how would you rate your sleep quality overall?	☐	☐	☐	☐
	**Not during the past month (0)**	**Less than once a week (1)**	**Once or twice a week (2)**	**Three or more times a week (3)**
7.During the past month, how often have you taken medicine (prescribed or “over the counter”) to help you sleep?	☐	☐	☐	☐

	**Not during the past month (0)**	**Less than once a week (1)**	**Once or twice a week (2)**	**Three or more times a week (3)**
8.During the past month, how often have you had trouble staying awake while driving, eating meals, or engaging in social activity?	☐	☐	☐	☐
	**No problem at all (0)**	**Only a very slight problem (1)**	**Somewhat of a problem (2)**	**A very big problem (3)**
9.During the past month, how much of a problem has it been for you to keep up enthusiasm to get things done?	☐	☐	☐	☐
	**No bed partner or room mate**	**Partner/room mate in other room**	**Partner in same room, but not same bed**	**Partner in same bed**
10.Do you have a bed partner or room mate?	☐	☐	☐	☐

**11.If you have a room-mate or partner, ask him/her how often in the past month you have had...**				
	**Not during the past month**	**Less than once a week**	**Once or twice a week**	**Three or more times a week**
a. loud snoring	☐	☐	☐	☐
b. long pauses between breaths while asleep	☐	☐	☐	☐
c. legs twitching or jerking while asleep	☐	☐	☐	☐
d. episodes of disorientation or confusion during sleep	☐	☐	☐	☐
e. other restlessness while you sleep; please describe				

PART THREE

ROSENBERG SELF-ESTEEM SCALE

Below is a list of statements dealing with your general feelings about yourself. Please indicate how strongly you agree or disagree with each statement.

	Strongly Agree	Agree	Disagree	Strongly Disagree
1. On the whole, I am satisfied with myself.	☐	☐	☐	☐
2. At times I think I am no good at all.	☐	☐	☐	☐
3. I feel that I have a number of good qualities.	☐	☐	☐	☐
4. I am able to do things as well as most other people.	☐	☐	☐	☐
5. I feel I do not have much to be proud of.	☐	☐	☐	☐
6. I certainly feel useless at times.	☐	☐	☐	☐
7. I feel that I'm a person of worth, at least on an equal plane with others.	☐	☐	☐	☐
8. I wish I could have more respect for myself.	☐	☐	☐	☐
9. All in all, I am inclined to feel that I am a failure.	☐	☐	☐	☐
10. I take a positive attitude toward myself.	☐	☐	☐	☐
