# Supplementary material for: Factors impacting university students’ quality of life
Source: PLoS One. 2025 Aug 6;20(8):e0329851. doi: 10.1371/journal.pone.0329851 (PMC12327633; doi:10.1371/journal.pone.0329851)
Supplement: S3 File — (PDF) [file pone.0329851.s003.pdf]

ID	Gender	Ageyrs	BMI	WaistCircumference	Faculty	Education	PlaceOfLiving	FatherOccupation	MotherWorkingStatus	ReligiousPractice Prayer	PhysicalExercise	AgeGroup	BodyMassIndex	WC	Family_Income	Monthly_Expenses	SELFESTEEMSCALE	Self_Esteem	Prception_QoL	Satisfaction_with_Health	PHYS	PSYCH	SOCIAL	ENVIR	PSQI	Sleep_Quality_Index	ScreenTime
1001.00	Female	22.00	18.44	31.94	Business and Entrepreneurship	3rd year	Home	Other jobs	Part time	Irregular	1.00	22-24	Underweight	>=31	>=51,000 BDT	<=20000 BDT	25.00	Low self-esteem	Good	Satisfied	53.57	54.17	50.00	34.38	14.00	Poor	5-8hrs
1002.00	Female	20.00	19.67	31.81	Business and Entrepreneurship	3rd year	Home	Business	Does not work	Regular	1.00	<=21	Normal	>=31	>=51,000 BDT	<=20000 BDT	20.00	Low self-esteem	Very good	Dissatisfied	46.43	54.17	66.67	53.13	12.00	Poor	5-8hrs
1003.00	Female	21.00	17.02	31.11	Business and Entrepreneurship	3rd year	Home	Professional	Full time	Regular	3.00	<=21	Underweight	>=31	>=51,000 BDT	<=20000 BDT	20.00	Low self-esteem	Very good	Very satisfied	46.43	54.17	66.67	56.25	9.00	Poor	5-8hrs
1004.00	Female	21.00	30.51	31.43	Business and Entrepreneurship	1st year	Hostel	Service	Full time	Regular	1.00	<=21	Obese	>=31	<=50,000 BDT	<=20000 BDT	23.00	Low self-esteem	Good	Satisfied	75.00	70.83	75.00	65.63	3.00	Good	5-8hrs
1005.00	Male	22.00	27.34	32.61	Humanities and Social Sciences	Final year	Home	Business	Does not work	Regular	3.00	22-24	Overweight	>=31	>=51,000 BDT	>=21000 BDT	19.00	Low self-esteem	Good	Satisfied	67.86	58.33	58.33	78.13	11.00	Poor	5-8hrs
1006.00	Female	21.00	20.16	31.48	Business and Entrepreneurship	1st year	Home	Service	Does not work	Irregular	4.00	<=21	Normal	>=31	<=50,000 BDT	>=21000 BDT	24.00	Low self-esteem	Good	Satisfied	64.29	54.17	66.67	75.00	11.00	Poor	5-8hrs
1007.00	Male	24.00	23.14	31.03	Humanities and Social Sciences	Final year	Home	Professional	Full time	Regular	4.00	22-24	Normal	>=31	>=51,000 BDT	>=21000 BDT	20.00	Low self-esteem	Good	Satisfied	53.57	37.50	66.67	53.13	12.00	Poor	<4hrs
1008.00	Male	23.00	23.38	31.78	Humanities and Social Sciences	Final year	Hostel	Business	Does not work	Irregular	4.00	22-24	Normal	>=31	>=51,000 BDT	>=21000 BDT	18.00	Low self-esteem	Neither poor nor good	Very satisfied	75.00	54.17	66.67	71.88	12.00	Poor	5-8hrs
1009.00	Male	23.00	30.26	31.44	Humanities and Social Sciences	Final year	Home	Professional	Does not work	Irregular	4.00	22-24	Obese	>=31	>=51,000 BDT	>=21000 BDT	17.00	Low self-esteem	Poor	Neither satisfied nor dissatisfied	50.00	45.83	33.33	50.00	4.00	Good	>8hrs
1010.00	Male	23.00	22.39	31.34	Humanities and Social Sciences	Final year	Hostel	Service	Does not work	Irregular	4.00	22-24	Normal	>=31	>=51,000 BDT	>=21000 BDT	24.00	Low self-esteem	Good	Satisfied	60.71	62.50	66.67	56.25	14.00	Poor	>8hrs
1011.00	Female	22.00	21.94	30.68	Business and Entrepreneurship	3rd year	Home	Business	Does not work	Regular	2.00	22-24	Normal	<=30 inch	<=50,000 BDT	>=21000 BDT	25.00	Low self-esteem	Neither poor nor good	Dissatisfied	71.43	54.17	58.33	59.38	11.00	Poor	<4hrs
1012.00	Female	23.00	24.61	39.57	Business and Entrepreneurship	3rd year	University Hall	Service	Does not work	Regular	2.00	22-24	Normal	>=31	<=50,000 BDT	>=21000 BDT	25.00	Low self-esteem	Neither poor nor good	Dissatisfied	71.43	54.17	58.33	59.38	11.00	Poor	<4hrs
1013.00	Female	21.00	26.48	32.09	Business and Entrepreneurship	3rd year	Hostel	Business	Does not work	Regular	3.00	<=21	Overweight	>=31	>=51,000 BDT	>=21000 BDT	26.00	Moderate self-esteem	Neither poor nor good	Satisfied	57.14	50.00	58.33	37.50	10.00	Poor	5-8hrs
1014.00	Female	23.00	16.76	31.55	Business and Entrepreneurship	3rd year	Home	Business	Does not work	Regular	4.00	22-24	Underweight	>=31	>=51,000 BDT	<=20000 BDT	23.00	Low self-esteem	Good	Satisfied	71.43	54.17	66.67	62.50	5.00	Good	5-8hrs
1015.00	Female	24.00	18.87	31.14	Business and Entrepreneurship	3rd year	Hostel	Business	Does not work	Regular	3.00	22-24	Normal	>=31	>=51,000 BDT	<=20000 BDT	26.00	Moderate self-esteem	Good	Dissatisfied	57.14	41.67	66.67	68.75	11.00	Poor	5-8hrs
1016.00	Female	20.00	20.80	38.25	Business and Entrepreneurship	3rd year	Home	Professional	Does not work	Regular	1.00	<=21	Normal	>=31	>=51,000 BDT	>=21000 BDT	22.00	Low self-esteem	Neither poor nor good	Satisfied	57.14	62.50	66.67	46.88	9.00	Poor	<4hrs
1017.00	Female	22.00	18.34	30.94	Business and Entrepreneurship	3rd year	Home	Business	Does not work	Regular	3.00	22-24	Underweight	<=30 inch	>=51,000 BDT	<=20000 BDT	16.00	Low self-esteem	Good	Neither satisfied nor dissatisfied	89.29	75.00	75.00	90.63	11.00	Poor	<4hrs
1018.00	Female	25.00	17.95	28.93	Business and Entrepreneurship	2nd year	Hostel	Business	Does not work	Regular	2.00	>=25	Underweight	<=30 inch	>=51,000 BDT	<=20000 BDT	20.00	Low self-esteem	Good	Satisfied	75.00	66.67	66.67	62.50	10.00	Poor	>8hrs
1019.00	Female	23.00	23.78	30.1	Business and Entrepreneurship	3rd year	University Hall	Business	Full time	Regular	3.00	22-24	Normal	<=30 inch	>=51,000 BDT	<=20000 BDT	22.00	Low self-esteem	Good	Satisfied	64.29	58.33	58.33	43.75	13.00	Poor	5-8hrs
1020.00	Female	22.00	21.48	30.24	Business and Entrepreneurship	3rd year	Home	Service	Part time	Regular	3.00	22-24	Normal	<=30 inch	>=51,000 BDT	>=21000 BDT	18.00	Low self-esteem	Good	Satisfied	78.57	83.33	75.00	65.63	9.00	Poor	<4hrs
1021.00	Male	22.00	30.23	34.38	Humanities and Social Sciences	2nd year	Hostel	Professional	Full time	Irregular	3.00	22-24	Obese	>=31	<=50,000 BDT	<=20000 BDT	19.00	Low self-esteem	Good	Satisfied	82.14	66.67	75.00	75.00	5.00	Good	5-8hrs
1022.00	Male	22.00	25.99	34.11	Humanities and Social Sciences	2nd year	Hostel	Service	Does not work	Irregular	4.00	22-24	Overweight	>=31	>=51,000 BDT	>=21000 BDT	29.00	Moderate self-esteem	Good	Satisfied	78.57	66.67	66.67	68.75	11.00	Poor	>8hrs
1023.00	Female	22.00	16.09	31.11	Business and Entrepreneurship	3rd year	Hostel	Service	Does not work	Regular	1.00	22-24	Underweight	>=31	<=50,000 BDT	<=20000 BDT	25.00	Low self-esteem	Good	Satisfied	67.86	54.17	75.00	75.00	11.00	Poor	<4hrs
1024.00	Male	23.00	30.01	34.41	Science and Information Technology	3rd year	Hostel	Business	Does not work	Irregular	4.00	22-24	Obese	>=31	<=50,000 BDT	>=21000 BDT	22.00	Low self-esteem	Neither poor nor good	Satisfied	78.57	70.83	66.67	65.63	9.00	Poor	5-8hrs
1025.00	Male	24.00	26.67	32.21	Science and Information Technology	3rd year	Home	No response	Does not work	Regular	4.00	22-24	Overweight	>=31	<=50,000 BDT	<=20000 BDT	20.00	Low self-esteem	Neither poor nor good	Satisfied	78.57	83.33	50.00	40.63	11.00	Poor	5-8hrs
1026.00	Male	24.00	20.79	33.26	Science and Information Technology	2nd year	Hostel	Business	Does not work	Irregular	3.00	22-24	Normal	>=31	<=50,000 BDT	>=21000 BDT	30.00	High self-esteem	Neither poor nor good	Neither satisfied nor dissatisfied	57.14	54.17	83.33	53.13	4.00	Good	5-8hrs
1027.00	Male	25.00	22.98	28.59	Science and Information Technology	3rd year	Hostel	Other jobs	Full time	Prefer not to say	1.00	>=25	Normal	<=30 inch	<=50,000 BDT	<=20000 BDT	21.00	Low self-esteem	Good	Satisfied	92.86	75.00	83.33	53.13	9.00	Poor	<4hrs
1028.00	Female	24.00	31.18	31.87	Business and Entrepreneurship	3rd year	Home	Service	Full time	Prefer not to say	3.00	22-24	Obese	>=31	>=51,000 BDT	>=21000 BDT	24.00	Low self-esteem	Good	Satisfied	82.14	66.67	66.67	78.13	4.00	Good	5-8hrs
1029.00	Female	22.00	21.67	36.33	Business and Entrepreneurship	3rd year	Hostel	Service	Does not work	Regular	4.00	22-24	Normal	>=31	>=51,000 BDT	<=20000 BDT	23.00	Low self-esteem	Good	Neither satisfied nor dissatisfied	67.86	45.83	50.00	50.00	3.00	Good	5-8hrs
1030.00	Female	25.00	20.05	31.89	Business and Entrepreneurship	Final year	Hostel	Service	Full time	Regular	3.00	>=25	Normal	>=31	>=51,000 BDT	<=20000 BDT	24.00	Low self-esteem	Good	Neither satisfied nor dissatisfied	71.43	62.50	66.67	56.25	12.00	Poor	5-8hrs
1031.00	Female	20.00	18.71	31.56	Business and Entrepreneurship	2nd year	Home	No response	Does not work	Regular	4.00	<=21	Normal	>=31	>=51,000 BDT	<=20000 BDT	17.00	Low self-esteem	Neither poor nor good	Neither satisfied nor dissatisfied	67.86	75.00	58.33	75.00	13.00	Poor	<4hrs
1032.00	Male	23.00	29.17	36.16	Science and Information Technology	2nd year	Home	Service	Does not work	Irregular	4.00	22-24	Overweight	>=31	<=50,000 BDT	<=20000 BDT	19.00	Low self-esteem	Good	Dissatisfied	71.43	83.33	58.33	71.88	3.00	Good	5-8hrs
1033.00	Male	22.00	24.22	31.93	Science and Information Technology	2nd year	Hostel	Service	Does not work	Irregular	4.00	22-24	Normal	>=31	>=51,000 BDT	<=20000 BDT	25.00	Low self-esteem	Good	Satisfied	57.14	54.17	50.00	59.38	14.00	Poor	<4hrs
1034.00	Female	20.00	25.72	31.62	Business and Entrepreneurship	2nd year	Home	Business	Full time	Regular	4.00	<=21	Overweight	>=31	>=51,000 BDT	<=20000 BDT	22.00	Low self-esteem	Good	Satisfied	57.14	79.17	75.00	56.25	14.00	Poor	5-8hrs
1035.00	Female	22.00	16.13	28.15	Business and Entrepreneurship	2nd year	Hostel	Service	Does not work	Regular	4.00	22-24	Underweight	<=30 inch	>=51,000 BDT	<=20000 BDT	18.00	Low self-esteem	Good	Satisfied	78.57	58.33	66.67	62.50	12.00	Poor	5-8hrs
1036.00	Male	23.00	28.52	36.64	Science and Information Technology	3rd year	Hostel	Professional	Does not work	Regular	3.00	22-24	Overweight	>=31	<=50,000 BDT	>=21000 BDT	21.00	Low self-esteem	Neither poor nor good	Neither satisfied nor dissatisfied	71.43	75.00	75.00	68.75	13.00	Poor	>8hrs
1037.00	Female	22.00	22.69	28.86	Business and Entrepreneurship	3rd year	Hostel	Business	Does not work	Irregular	4.00	22-24	Normal	<=30 inch	<=50,000 BDT	<=20000 BDT	26.00	Moderate self-esteem	Neither poor nor good	Dissatisfied	71.43	66.67	66.67	56.25	14.00	Poor	5-8hrs
1038.00	Male	21.00	22.17	31.19	Science and Information Technology	2nd year	Hostel	Business	Does not work	Irregular	4.00	<=21	Normal	>=31	>=51,000 BDT	<=20000 BDT	21.00	Low self-esteem	Good	Very satisfied	85.71	83.33	100.00	78.13	1.00	Good	5-8hrs
1039.00	Female	20.00	19.08	37.64	Business and Entrepreneurship	2nd year	Home	Service	Does not work	Regular	4.00	<=21	Normal	>=31	>=51,000 BDT	<=20000 BDT	32.00	High self-esteem	Good	Dissatisfied	32.14	33.33	50.00	56.25	11.00	Poor	5-8hrs
1040.00	Female	23.00	24.92	39.3	Business and Entrepreneurship	3rd year	University Hall	Service	Does not work	Regular	2.00	22-24	Overweight	>=31	<=50,000 BDT	<=20000 BDT	24.00	Low self-esteem	Neither poor nor good	Dissatisfied	71.43	54.17	66.67	53.13	9.00	Poor	5-8hrs
1041.00	Male	24.00	21.53	29.03	Science and Information Technology	Final year	Hostel	Business	Does not work	Irregular	3.00	22-24	Normal	<=30 inch	>=51,000 BDT	<=20000 BDT	24.00	Low self-esteem	Good	Satisfied	92.86	66.67	66.67	59.38	5.00	Good	5-8hrs
1042.00	Female	22.00	27.62	31.69	Business and Entrepreneurship	3rd year	Home	Business	Full time	Regular	4.00	22-24	Overweight	>=31	>=51,000 BDT	<=20000 BDT	25.00	Low self-esteem	Neither poor nor good	Satisfied	71.43	29.17	75.00	62.50	4.00	Good	5-8hrs
1043.00	Female	21.00	20.02	31.95	Business and Entrepreneurship	3rd year	Hostel	Service	Does not work	Regular	2.00	<=21	Normal	>=31	>=51,000 BDT	<=20000 BDT	25.00	Low self-esteem	Neither poor nor good	Satisfied	53.57	41.67	50.00	43.75	2.00	Good	5-8hrs
1044.00	Female	23.00	21.77	31.43	Business and Entrepreneurship	3rd year	Hostel	Service	Does not work	Prefer not to say	4.00	22-24	Normal	>=31	>=51,000 BDT	<=20000 BDT	25.00	Low self-esteem	Good	Neither satisfied nor dissatisfied	64.29	62.50	58.33	43.75	12.00	Poor	<4hrs
1045.00	Male	22.00	28.13	31.25	Science and Information Technology	3rd year	Hostel	Business	Does not work	Irregular	4.00	22-24	Overweight	>=31	<=50,000 BDT	>=21000 BDT	15.00	Low self-esteem	Good	Satisfied	64.29	70.83	75.00	78.13	9.00	Poor	>8hrs
1046.00	Male	23.00	16.60	29.71	Science and Information Technology	3rd year	Hostel	Business	Does not work	Regular	1.00	22-24	Underweight	<=30 inch	<=50,000 BDT	>=21000 BDT	20.00	Low self-esteem	Good	Satisfied	75.00	83.33	50.00	37.50	4.00	Good	<4hrs
1047.00	Male	24.00	29.17	31.21	Science and Information Technology	3rd year	Home	Business	Does not work	Regular	3.00	22-24	Overweight	>=31	>=51,000 BDT	>=21000 BDT	19.00	Low self-esteem	Good	Neither satisfied nor dissatisfied	67.86	70.83	58.33	56.25	9.00	Poor	5-8hrs
1048.00	Female	23.00	20.56	31.27	Business and Entrepreneurship	3rd year	Hostel	Business	Full time	Prefer not to say	4.00	22-24	Normal	>=31	>=51,000 BDT	<=20000 BDT	22.00	Low self-esteem	Good	Satisfied	71.43	62.50	50.00	81.25	4.00	Good	5-8hrs
1049.00	Female	21.00	20.91	31.13	Business and Entrepreneurship	2nd year	Hostel	Business	Part time	Irregular	3.00	<=21	Normal	>=31	>=51,000 BDT	<=20000 BDT	24.00	Low self-esteem	Neither poor nor good	Neither satisfied nor dissatisfied	64.29	41.67	33.33	46.88	11.00	Poor	>8hrs
1050.00	Male	22.00	19.44	31.47	Science and Information Technology	2nd year	Hostel	Business	Does not work	Regular	4.00	22-24	Normal	>=31	>=51,000 BDT	<=20000 BDT	25.00	Low self-esteem	Good	Neither satisfied nor dissatisfied	67.86	45.83	41.67	43.75	11.00	Poor	<4hrs
1051.00	Male	21.00	21.94	31.94	Science and Information Technology	2nd year	Relatives house	Business	Does not work	Regular	3.00	<=21	Normal	>=31	>=51,000 BDT	<=20000 BDT	26.00	Moderate self-esteem	Neither poor nor good	Satisfied	71.43	58.33	50.00	53.13	9.00	Poor	5-8hrs
1052.00	Male	21.00	23.07	30.34	Science and Information Technology	2nd year	Home	Business	Does not work	Prefer not to say	4.00	<=21	Normal	<=30 inch	<=50,000 BDT	<=20000 BDT	20.00	Low self-esteem	Very good	Very satisfied	89.29	83.33	83.33	87.50	5.00	Good	5-8hrs
1053.00	Female	22.00	16.02	28.41	Business and Entrepreneurship	2nd year	Home	Professional	Does not work	Irregular	4.00	22-24	Underweight	<=30 inch	<=50,000 BDT	>=21000 BDT	22.00	Low self-esteem	Good	Neither satisfied nor dissatisfied	53.57	54.17	66.67	59.38	3.00	Good	<4hrs
1054.00	Male	24.00	20.70	32.81	Science and Information Technology	2nd year	Hostel	Service	Does not work	Regular	4.00	22-24	Normal	>=31	>=51,000 BDT	>=21000 BDT	27.00	Moderate self-esteem	Poor	Very dissatisfied	28.57	20.83	100.00	50.00	14.00	Poor	5-8hrs
1055.00	Male	23.00	21.94	32.94	Science and Information Technology	2nd year	Home	Business	Does not work	Irregular	4.00	22-24	Normal	>=31	>=51,000 BDT	<=20000 BDT	21.00	Low self-esteem	Neither poor nor good	Dissatisfied	42.86	75.00	66.67	15.63	10.00	Poor	>8hrs
1056.00	Female	22.00	15.07	31.68	Business and Entrepreneurship	2nd year	Hostel	Service	Does not work	Irregular	4.00	22-24	Underweight	>=31	>=51,000 BDT	<=20000 BDT	32.00	High self-esteem	Very poor	Dissatisfied	21.43	41.67	58.33	50.00	10.00	Poor	>8hrs
1057.00	Male	20.00	21.48	31.49	Science and Information Technology	2nd year	Hostel	Professional	Does not work	Irregular	3.00	<=21	Normal	>=31	>=51,000 BDT	<=20000 BDT	22.00	Low self-esteem	Good	Neither satisfied nor dissatisfied	71.43	62.50	75.00	75.00	3.00	Good	5-8hrs
1058.00	Male	21.00	29.30	31.14	Science and Information Technology	1st year	Hostel	Business	Does not work	Prefer not to say	4.00	<=21	Overweight	>=31	>=51,000 BDT	>=21000 BDT	23.00	Low self-esteem	Neither poor nor good	Neither satisfied nor dissatisfied	71.43	66.67	58.33	59.38	11.00	Poor	5-8hrs
1059.00	Female	21.00	25.39	31.77	Business and Entrepreneurship	2nd year	Hostel	Service	Full time	Irregular	4.00	<=21	Overweight	>=31	>=51,000 BDT	<=20000 BDT	23.00	Low self-esteem	Good	Neither satisfied nor dissatisfied	64.29	45.83	41.67	37.50	4.00	Good	>8hrs
1060.00	Female	21.00	18.91	30.87	Business and Entrepreneurship	2nd year	Home	Business	Does not work	Irregular	4.00	<=21	Normal	<=30 inch	>=51,000 BDT	<=20000 BDT	25.00	Low self-esteem	Neither poor nor good	Satisfied	60.71	50.00	41.67	53.13	10.00	Poor	>8hrs
1061.00	Male	22.00	23.11	31.71	Science and Information Technology	2nd year	Hostel	Service	Does not work	Regular	4.00	22-24	Normal	>=31	>=51,000 BDT	<=20000 BDT	21.00	Low self-esteem	Good	Satisfied	60.71	66.67	41.67	53.13	3.00	Good	<4hrs
1062.00	Male	23.00	16.51	28.9	Science and Information Technology	2nd year	Hostel	Service	Does not work	Irregular	3.00	22-24	Underweight	<=30 inch	>=51,000 BDT	>=21000 BDT	24.00	Low self-esteem	Neither poor nor good	Neither satisfied nor dissatisfied	57.14	50.00	75.00	56.25	4.00	Good	5-8hrs
1063.00	Male	22.00	24.97	31.81	Science and Information Technology	2nd year	Home	Business	Does not work	Irregular	4.00	22-24	Overweight	>=31	>=51,000 BDT	<=20000 BDT	22.00	Low self-esteem	Neither poor nor good	Dissatisfied	46.43	37.50	50.00	34.38	12.00	Poor	5-8hrs
1064.00	Female	22.00	19.94	30.98	Business and Entrepreneurship	2nd year	Home	Business	Does not work	Regular	1.00	22-24	Normal	<=30 inch	>=51,000 BDT	>=21000 BDT	23.00	Low self-esteem	Neither poor nor good	Satisfied	64.29	45.83	58.33	21.88	14.00	Poor	5-8hrs
1065.00	Male	21.00	23.07	30.23	Science and Information Technology	2nd year	Home	Business	Does not work	Prefer not to say	4.00	<=21	Normal	<=30 inch	<=50,000 BDT	<=20000 BDT	20.00	Low self-esteem	Very good	Very satisfied	89.29	83.33	83.33	87.50	5.00	Good	5-8hrs
1066.00	Female	22.00	16.76	31.53	Business and Entrepreneurship	2nd year	Home	Service	Does not work	Regular	4.00	22-24	Underweight	>=31	>=51,000 BDT	<=20000 BDT	17.00	Low self-esteem	Good	Satisfied	78.57	58.33	66.67	62.50	13.00	Poor	5-8hrs
1067.00	Female	22.00	25.80	31.87	Business and Entrepreneurship	2nd year	Home	Service	Does not work	Regular	4.00	22-24	Overweight	>=31	>=51,000 BDT	<=20000 BDT	17.00	Low self-esteem	Good	Satisfied	78.57	58.33	66.67	62.50	13.00	Poor	5-8hrs
1068.00	Female	20.00	23.38	31.74	Business and Entrepreneurship	2nd year	Hostel	Business	Does not work	Regular	4.00	<=21	Normal	>=31	>=51,000 BDT	<=20000 BDT	19.00	Low self-esteem	Neither poor nor good	Neither satisfied nor dissatisfied	71.43	50.00	66.67	46.88	9.00	Poor	<4hrs
1069.00	Female	22.00	20.96	30.49	Business and Entrepreneurship	2nd year	Home	Business	Does not work	Regular	3.00	22-24	Normal	<=30 inch	>=51,000 BDT	<=20000 BDT	25.00	Low self-esteem	Good	Satisfied	75.00	62.50	66.67	46.88	13.00	Poor	5-8hrs
1070.00	Female	21.00	22.98	31.49	Business and Entrepreneurship	2nd year	Home	Business	Does not work	Regular	4.00	<=21	Normal	>=31	>=51,000 BDT	<=20000 BDT	22.00	Low self-esteem	Good	Satisfied	64.29	50.00	50.00	59.38	9.00	Poor	5-8hrs
1071.00	Female	23.00	24.17	31.71	Business and Entrepreneurship	2nd year	Hostel	Business	Full time	Irregular	4.00	22-24	Normal	>=31	>=51,000 BDT	<=20000 BDT	19.00	Low self-esteem	Neither poor nor good	Neither satisfied nor dissatisfied	71.43	50.00	41.67	40.63	11.00	Poor	>8hrs
1072.00	Male	23.00	25.40	32.23	Science and Information Technology	3rd year	Hostel	Professional	Full time	Regular	2.00	22-24	Overweight	>=31	>=51,000 BDT	<=20000 BDT	22.00	Low self-esteem	Neither poor nor good	Satisfied	64.29	45.83	58.33	40.63	9.00	Poor	<4hrs

1073.00	Male	22.00	24.11	30.04	Science and Information Technology	3rd year	Home	Business	Does not work	Irregular	4.00	22-24	Normal	<=30 inch	>=51,000 BDT	>=21000 BDT	25.00	Low self-esteem	Neither poor nor good	Neither satisfied nor dissatisfied	42.86	29.17	50.00	40.63	9.00	Poor	5-8hrs
1074.00	Female	24.00	23.76	31.43	Business and Entrepreneurship	3rd year	Hostel	Service	Does not work	Regular	3.00	22-24	Normal	>=31	>=51,000 BDT	<=20000 BDT	25.00	Low self-esteem	Good	Dissatisfied	67.86	45.83	50.00	62.50	14.00	Poor	5-8hrs
1075.00	Female	22.00	21.37	31.2	Business and Entrepreneurship	3rd year	University Hall	Service	Does not work	Irregular	4.00	22-24	Normal	>=31	>=51,000 BDT	<=20000 BDT	28.00	Moderate self-esteem	Good	Dissatisfied	53.57	66.67	66.67	37.50	10.00	Poor	>8hrs
1076.00	Female	22.00	15.38	31.25	Business and Entrepreneurship	3rd year	Hostel	Service	Does not work	Irregular	3.00	22-24	Underweight	>=31	>=51,000 BDT	<=20000 BDT	22.00	Low self-esteem	Good	Neither satisfied nor dissatisfied	67.86	45.83	50.00	59.38	5.00	Good	5-8hrs
1077.00	Male	24.00	26.20	32.67	Science and Information Technology	3rd year	Home	Service	Does not work	Regular	1.00	22-24	Overweight	>=31	>=51,000 BDT	<=20000 BDT	13.00	Low self-esteem	Very good	Very satisfied	100.00	95.83	100.00	93.75	4.00	Good	5-8hrs
1078.00	Female	22.00	17.78	31.32	Business and Entrepreneurship	3rd year	Home	Service	Does not work	Prefer not to say	4.00	22-24	Underweight	>=31	>=51,000 BDT	<=20000 BDT	24.00	Low self-esteem	Neither poor nor good	Satisfied	50.00	45.83	50.00	34.38	12.00	Poor	5-8hrs
1079.00	Male	22.00	30.42	31.55	Science and Information Technology	3rd year	Hostel	Service	Does not work	Irregular	3.00	22-24	Obese	>=31	>=51,000 BDT	<=20000 BDT	20.00	Low self-esteem	Good	Neither satisfied nor dissatisfied	71.43	75.00	66.67	71.88	3.00	Good	5-8hrs
1080.00	Male	23.00	31.04	31.37	Science and Information Technology	2nd year	Hostel	Professional	Does not work	Irregular	3.00	22-24	Obese	>=31	<=50,000 BDT	>=21000 BDT	26.00	Moderate self-esteem	Good	Satisfied	50.00	58.33	66.67	43.75	5.00	Good	5-8hrs
1081.00	Female	22.00	15.32	28.97	Business and Entrepreneurship	3rd year	Hostel	Service	Does not work	Irregular	4.00	22-24	Underweight	<=30 inch	<=50,000 BDT	>=21000 BDT	31.00	High self-esteem	Good	Neither satisfied nor dissatisfied	53.57	45.83	83.33	75.00	11.00	Poor	<4hrs
1082.00	Female	23.00	16.02	31.93	Business and Entrepreneurship	3rd year	Home	No response	Does not work	Prefer not to say	4.00	22-24	Underweight	>=31	>=51,000 BDT	<=20000 BDT	31.00	High self-esteem	Neither poor nor good	Satisfied	39.29	37.50	58.33	53.13	9.00	Poor	5-8hrs
1083.00	Female	22.00	17.90	31.08	Business and Entrepreneurship	3rd year	Home	Business	Does not work	Regular	4.00	22-24	Underweight	>=31	>=51,000 BDT	<=20000 BDT	23.00	Low self-esteem	Good	Neither satisfied nor dissatisfied	53.57	54.17	58.33	46.88	14.00	Poor	>8hrs
1084.00	Female	23.00	22.09	31.85	Business and Entrepreneurship	3rd year	Home	No response	Does not work	Regular	4.00	22-24	Normal	>=31	>=51,000 BDT	<=20000 BDT	28.00	Moderate self-esteem	Neither poor nor good	Dissatisfied	39.29	50.00	25.00	46.88	9.00	Poor	5-8hrs
1085.00	Female	21.00	23.85	31.57	Business and Entrepreneurship	2nd year	Home	Service	Part time	Regular	3.00	<=21	Normal	>=31	<=50,000 BDT	<=20000 BDT	26.00	Moderate self-esteem	Neither poor nor good	Dissatisfied	46.43	41.67	66.67	43.75	13.00	Poor	>8hrs
1086.00	Female	23.00	25.40	31.62	Business and Entrepreneurship	3rd year	Home	Service	Does not work	Regular	4.00	22-24	Overweight	>=31	>=51,000 BDT	<=20000 BDT	21.00	Low self-esteem	Good	Dissatisfied	71.43	54.17	66.67	50.00	3.00	Good	<4hrs
1087.00	Female	23.00	20.96	31.72	Business and Entrepreneurship	3rd year	Hostel	Business	Does not work	Regular	4.00	22-24	Normal	>=31	>=51,000 BDT	<=20000 BDT	21.00	Low self-esteem	Good	Satisfied	75.00	58.33	66.67	59.38	4.00	Good	<4hrs
1088.00	Male	24.00	28.76	32.09	Science and Information Technology	3rd year	Home	Service	Does not work	Irregular	4.00	22-24	Overweight	>=31	<=50,000 BDT	<=20000 BDT	17.00	Low self-esteem	Neither poor nor good	Satisfied	64.29	50.00	66.67	28.13	12.00	Poor	>8hrs
1089.00	Female	21.00	17.61	30.35	Business and Entrepreneurship	2nd year	Hostel	Business	Does not work	Regular	4.00	<=21	Underweight	<=30 inch	>=51,000 BDT	>=21000 BDT	27.00	Moderate self-esteem	Neither poor nor good	Dissatisfied	67.86	54.17	25.00	34.38	12.00	Poor	5-8hrs
1090.00	Male	22.00	29.30	31.72	Science and Information Technology	2nd year	Hostel	Business	Does not work	Prefer not to say	4.00	22-24	Overweight	>=31	>=51,000 BDT	<=20000 BDT	23.00	Low self-esteem	Very poor	Neither satisfied nor dissatisfied	39.29	41.67	66.67	50.00	10.00	Poor	5-8hrs
1091.00	Female	20.00	19.22	28.56	Business and Entrepreneurship	2nd year	Hostel	Service	Part time	Irregular	4.00	<=21	Normal	<=30 inch	>=51,000 BDT	>=21000 BDT	21.00	Low self-esteem	Good	Satisfied	71.43	62.50	50.00	59.38	10.00	Poor	5-8hrs
1092.00	Male	22.00	21.00	32.41	Science and Information Technology	1st year	University Hall	Business	Does not work	Irregular	3.00	22-24	Normal	>=31	>=51,000 BDT	>=21000 BDT	15.00	Low self-esteem	Good	Satisfied	92.86	75.00	58.33	62.50	5.00	Good	5-8hrs
1093.00	Female	20.00	18.78	31.38	Business and Entrepreneurship	2nd year	Hostel	Business	Does not work	Irregular	4.00	<=21	Normal	>=31	>=51,000 BDT	>=21000 BDT	24.00	Low self-esteem	Neither poor nor good	Satisfied	67.86	50.00	66.67	53.13	5.00	Good	5-8hrs
1094.00	Female	21.00	21.18	31.38	Business and Entrepreneurship	2nd year	Hostel	Business	Does not work	Prefer not to say	3.00	<=21	Normal	>=31	>=51,000 BDT	<=20000 BDT	18.00	Low self-esteem	Good	Neither satisfied nor dissatisfied	64.29	66.67	58.33	50.00	10.00	Poor	5-8hrs
1095.00	Female	20.00	18.87	31.18	Business and Entrepreneurship	2nd year	Hostel	Business	Does not work	Prefer not to say	4.00	<=21	Normal	>=31	>=51,000 BDT	<=20000 BDT	18.00	Low self-esteem	Neither poor nor good	Satisfied	35.71	37.50	58.33	53.13	9.00	Poor	5-8hrs
1096.00	Male	22.00	34.01	38.39	Science and Information Technology	1st year	University Hall	Professional	Full time	Irregular	1.00	22-24	Obese	>=31	>=51,000 BDT	>=21000 BDT	15.00	Low self-esteem	Very good	Satisfied	75.00	83.33	75.00	87.50	1.00	Good	<4hrs
1097.00	Male	22.00	31.86	32.68	Science and Information Technology	2nd year	University Hall	Business	Does not work	Irregular	3.00	22-24	Obese	>=31	>=51,000 BDT	<=20000 BDT	16.00	Low self-esteem	Poor	Dissatisfied	67.86	54.17	50.00	34.38	5.00	Good	>8hrs
1098.00	Female	22.00	21.48	31.79	Business and Entrepreneurship	2nd year	University Hall	Service	Full time	Irregular	4.00	22-24	Normal	>=31	>=51,000 BDT	<=20000 BDT	24.00	Low self-esteem	Very poor	Very dissatisfied	50.00	41.67	58.33	59.38	10.00	Poor	5-8hrs
1099.00	Male	22.00	22.69	32.66	Science and Information Technology	2nd year	University Hall	Business	Does not work	Irregular	4.00	22-24	Normal	>=31	>=51,000 BDT	>=21000 BDT	28.00	Moderate self-esteem	Good	Neither satisfied nor dissatisfied	46.43	37.50	75.00	59.38	4.00	Good	5-8hrs
1100.00	Female	22.00	23.38	31.98	Business and Entrepreneurship	2nd year	Hostel	Business	Does not work	Regular	4.00	22-24	Normal	>=31	>=51,000 BDT	<=20000 BDT	21.00	Low self-esteem	Very good	Neither satisfied nor dissatisfied	64.29	79.17	75.00	65.63	12.00	Poor	>8hrs
1101.00	Female	20.00	20.96	28.25	Business and Entrepreneurship	2nd year	Home	Service	Does not work	Irregular	4.00	<=21	Normal	<=30 inch	>=51,000 BDT	>=21000 BDT	23.00	Low self-esteem	Neither poor nor good	Neither satisfied nor dissatisfied	53.57	50.00	41.67	40.63	14.00	Poor	5-8hrs
1102.00	Female	22.00	20.80	31.48	Business and Entrepreneurship	3rd year	Hostel	Business	Full time	Irregular	4.00	22-24	Normal	>=31	<=50,000 BDT	<=20000 BDT	17.00	Low self-esteem	Neither poor nor good	Dissatisfied	67.86	70.83	66.67	46.88	10.00	Poor	>8hrs
1103.00	Female	22.00	28.37	31.67	Business and Entrepreneurship	3rd year	Home	Service	Full time	Irregular	1.00	22-24	Overweight	>=31	<=50,000 BDT	<=20000 BDT	21.00	Low self-esteem	Good	Satisfied	82.14	70.83	66.67	68.75	5.00	Good	>8hrs
1104.00	Female	22.00	19.94	31.35	Business and Entrepreneurship	3rd year	Hostel	Business	Does not work	Prefer not to say	4.00	22-24	Normal	>=31	>=51,000 BDT	<=20000 BDT	21.00	Low self-esteem	Good	Neither satisfied nor dissatisfied	75.00	54.17	50.00	62.50	4.00	Good	5-8hrs
1105.00	Female	23.00	19.52	37.74	Business and Entrepreneurship	3rd year	Home	Business	Does not work	Regular	4.00	22-24	Normal	>=31	>=51,000 BDT	>=21000 BDT	26.00	Moderate self-esteem	Neither poor nor good	Satisfied	67.86	50.00	58.33	56.25	4.00	Good	5-8hrs
1106.00	Male	22.00	32.25	36.65	Science and Information Technology	3rd year	Home	Service	Does not work	Irregular	3.00	22-24	Obese	>=31	>=51,000 BDT	<=20000 BDT	22.00	Low self-esteem	Neither poor nor good	Neither satisfied nor dissatisfied	42.86	83.33	83.33	46.88	11.00	Poor	5-8hrs
1107.00	Female	20.00	21.88	31.7	Business and Entrepreneurship	3rd year	Relatives house	Business	Does not work	Regular	4.00	<=21	Normal	>=31	>=51,000 BDT	<=20000 BDT	29.00	Moderate self-esteem	Neither poor nor good	Neither satisfied nor dissatisfied	53.57	25.00	41.67	37.50	11.00	Poor	5-8hrs
1108.00	Male	24.00	31.33	37.89	Science and Information Technology	3rd year	Home	Business	Full time	Regular	4.00	22-24	Obese	>=31	>=51,000 BDT	>=21000 BDT	18.00	Low self-esteem	Good	Neither satisfied nor dissatisfied	82.14	66.67	66.67	68.75	12.00	Poor	5-8hrs
1109.00	Female	24.00	23.38	31.13	Business and Entrepreneurship	3rd year	Hostel	Service	Does not work	Regular	3.00	22-24	Normal	>=31	>=51,000 BDT	<=20000 BDT	15.00	Low self-esteem	Good	Satisfied	82.14	75.00	50.00	75.00	4.00	Good	5-8hrs
1110.00	Female	24.00	21.18	31.22	Business and Entrepreneurship	3rd year	Home	No response	Does not work	Irregular	4.00	22-24	Normal	>=31	>=51,000 BDT	<=20000 BDT	26.00	Moderate self-esteem	Neither poor nor good	Satisfied	50.00	50.00	66.67	56.25	10.00	Poor	>8hrs
1111.00	Female	22.00	21.81	31.67	Business and Entrepreneurship	3rd year	Hostel	Professional	Full time	Regular	4.00	22-24	Normal	>=31	>=51,000 BDT	<=20000 BDT	28.00	Moderate self-esteem	Neither poor nor good	Satisfied	53.57	37.50	25.00	40.63	12.00	Poor	5-8hrs
1112.00	Female	25.00	25.68	30.5	Business and Entrepreneurship	3rd year	Home	Business	Does not work	Prefer not to say	4.00	>=25	Overweight	<=30 inch	<=50,000 BDT	<=20000 BDT	26.00	Moderate self-esteem	Good	Neither satisfied nor dissatisfied	67.86	54.17	66.67	59.38	4.00	Good	5-8hrs
1113.00	Female	22.00	19.53	30.85	Business and Entrepreneurship	3rd year	Home	Business	Does not work	Regular	4.00	22-24	Normal	<=30 inch	>=51,000 BDT	>=21000 BDT	14.00	Low self-esteem	Good	Satisfied	75.00	75.00	58.33	56.25	10.00	Poor	5-8hrs
1114.00	Female	21.00	23.44	30.94	Business and Entrepreneurship	1st year	Home	Business	Full time	Irregular	4.00	<=21	Normal	<=30 inch	>=51,000 BDT	>=21000 BDT	26.00	Moderate self-esteem	Good	Neither satisfied nor dissatisfied	67.86	50.00	75.00	53.13	9.00	Poor	5-8hrs
1115.00	Female	23.00	22.17	31.16	Business and Entrepreneurship	Final year	Home	Service	Full time	Regular	3.00	22-24	Normal	>=31	>=51,000 BDT	<=20000 BDT	26.00	Moderate self-esteem	Good	Satisfied	75.00	58.33	66.67	78.13	11.00	Poor	5-8hrs
1116.00	Female	20.00	15.29	31.23	Business and Entrepreneurship	2nd year	Home	Service	Does not work	Regular	3.00	<=21	Underweight	>=31	<=50,000 BDT	<=20000 BDT	20.00	Low self-esteem	Neither poor nor good	Satisfied	71.43	75.00	66.67	56.25	4.00	Good	<4hrs
1117.00	Female	24.00	26.69	31.21	Business and Entrepreneurship	3rd year	Hostel	No response	Does not work	Irregular	4.00	22-24	Overweight	>=31	<=50,000 BDT	>=21000 BDT	27.00	Moderate self-esteem	Good	Dissatisfied	75.00	50.00	58.33	50.00	5.00	Good	5-8hrs
1118.00	Male	24.00	25.40	32.33	Science and Information Technology	3rd year	University Hall	No response	Does not work	Irregular	2.00	22-24	Overweight	>=31	<=50,000 BDT	>=21000 BDT	21.00	Low self-esteem	Good	Very satisfied	78.57	75.00	75.00	71.88	9.00	Poor	5-8hrs
1119.00	Male	24.00	19.14	28.04	Science and Information Technology	3rd year	Hostel	No response	Does not work	Irregular	4.00	22-24	Normal	<=30 inch	<=50,000 BDT	>=21000 BDT	24.00	Low self-esteem	Good	Neither satisfied nor dissatisfied	71.43	50.00	75.00	56.25	13.00	Poor	<4hrs
1120.00	Male	25.00	28.34	34.64	Science and Information Technology	3rd year	University Hall	Professional	Does not work	Prefer not to say	3.00	>=25	Overweight	>=31	>=51,000 BDT	>=21000 BDT	21.00	Low self-esteem	Neither poor nor good	Neither satisfied nor dissatisfied	64.29	58.33	50.00	46.88	4.00	Good	<4hrs
1121.00	Female	21.00	24.17	31.67	Business and Entrepreneurship	2nd year	Hostel	Business	Does not work	Prefer not to say	4.00	<=21	Normal	>=31	<=50,000 BDT	<=20000 BDT	25.00	Low self-esteem	Good	Neither satisfied nor dissatisfied	64.29	37.50	58.33	37.50	9.00	Poor	5-8hrs
1122.00	Female	23.00	27.99	28.29	Business and Entrepreneurship	3rd year	Home	Business	Does not work	Irregular	4.00	22-24	Overweight	<=30 inch	>=51,000 BDT	<=20000 BDT	22.00	Low self-esteem	Good	Neither satisfied nor dissatisfied	60.71	16.67	33.33	59.38	15.00	Poor	>8hrs
1123.00	Female	23.00	25.39	31.97	Business and Entrepreneurship	3rd year	University Hall	Service	Does not work	Irregular	4.00	22-24	Overweight	>=31	>=51,000 BDT	<=20000 BDT	27.00	Moderate self-esteem	Neither poor nor good	Dissatisfied	75.00	45.83	75.00	59.38	12.00	Poor	>8hrs
1124.00	Male	23.00	31.00	40.69	Science and Information Technology	3rd year	Hostel	Service	Does not work	Irregular	4.00	22-24	Obese	>=31	<=50,000 BDT	<=20000 BDT	31.00	High self-esteem	Neither poor nor good	Dissatisfied	67.86	70.83	75.00	40.63	9.00	Poor	>8hrs
1125.00	Female	22.00	16.76	31.84	Business and Entrepreneurship	3rd year	Home	Business	Does not work	Irregular	4.00	22-24	Underweight	>=31	>=51,000 BDT	<=20000 BDT	29.00	Moderate self-esteem	Very poor	Dissatisfied	25.00	33.33	66.67	28.13	11.00	Poor	5-8hrs
1126.00	Male	22.00	30.14	31.81	Science and Information Technology	2nd year	Home	Service	Does not work	Regular	3.00	22-24	Obese	>=31	<=50,000 BDT	<=20000 BDT	19.00	Low self-esteem	Neither poor nor good	Neither satisfied nor dissatisfied	67.86	70.83	75.00	34.38	5.00	Good	<4hrs
1127.00	Female	22.00	22.51	31.75	Business and Entrepreneurship	3rd year	Home	Service	Does not work	Regular	4.00	22-24	Normal	>=31	>=51,000 BDT	<=20000 BDT	27.00	Moderate self-esteem	Neither poor nor good	Dissatisfied	46.43	41.67	50.00	37.50	12.00	Poor	5-8hrs
1128.00	Female	20.00	22.51	31.12	Business and Entrepreneurship	2nd year	Hostel	Service	Full time	Irregular	4.00	<=21	Normal	>=31	>=51,000 BDT	<=20000 BDT	22.00	Low self-esteem	Good	Satisfied	67.86	66.67	66.67	43.75	11.00	Poor	<4hrs
1129.00	Female	20.00	16.13	30.52	Business and Entrepreneurship	2nd year	University Hall	Professional	Full time	Regular	4.00	<=21	Underweight	<=30 inch	>=51,000 BDT	>=21000 BDT	20.00	Low self-esteem	Good	Neither satisfied nor dissatisfied	57.14	83.33	58.33	46.88	9.00	Poor	5-8hrs
1130.00	Female	20.00	21.12	35.23	Business and Entrepreneurship	2nd year	Hostel	Business	Does not work	Regular	4.00	<=21	Normal	>=31	>=51,000 BDT	>=21000 BDT	24.00	Low self-esteem	Good	Satisfied	60.71	58.33	75.00	40.63	9.00	Poor	<4hrs
1131.00	Female	21.00	19.52	31.28	Business and Entrepreneurship	2nd year	Hostel	No response	Does not work	Prefer not to say	4.00	<=21	Normal	>=31	>=51,000 BDT	<=20000 BDT	21.00	Low self-esteem	Good	Neither satisfied nor dissatisfied	53.57	45.83	50.00	62.50	10.00	Poor	5-8hrs
1132.00	Male	24.00	31.86	31.21	Science and Information Technology	3rd year	Home	No response	Full time	Irregular	3.00	22-24	Obese	>=31	<=50,000 BDT	<=20000 BDT	25.00	Low self-esteem	Very poor	Satisfied	53.57	58.33	75.00	56.25	5.00	Good	5-8hrs
1133.00	Female	21.00	22.27	31.48	Business and Entrepreneurship	2nd year	Home	Service	Full time	Prefer not to say	4.00	<=21	Normal	>=31	>=51,000 BDT	>=21000 BDT	24.00	Low self-esteem	Neither poor nor good	Neither satisfied nor dissatisfied	57.14	45.83	58.33	31.25	10.00	Poor	<4hrs
1134.00	Female	21.00	25.40	31.11	Business and Entrepreneurship	2nd year	Home	Business	Does not work	Prefer not to say	4.00	<=21	Overweight	>=31	>=51,000 BDT	<=20000 BDT	24.00	Low self-esteem	Good	Dissatisfied	39.29	50.00	50.00	50.00	14.00	Poor	5-8hrs
1135.00	Female	21.00	21.56	31.3	Business and Entrepreneurship	2nd year	Hostel	Business	Full time	Irregular	4.00	<=21	Normal	>=31	>=51,000 BDT	<=20000 BDT	28.00	Moderate self-esteem	Neither poor nor good	Dissatisfied	71.43	37.50	58.33	46.88	10.00	Poor	5-8hrs
1136.00	Female	21.00	18.54	26.13	Business and Entrepreneurship	2nd year	Hostel	Business	Does not work	Irregular	3.00	<=21	Normal	<=30 inch	>=51,000 BDT	<=20000 BDT	25.00	Low self-esteem	Neither poor nor good	Neither satisfied nor dissatisfied	53.57	58.33	50.00	40.63	15.00	Poor	5-8hrs
1137.00	Female	21.00	17.65	28.57	Business and Entrepreneurship	2nd year	Hostel	Business	Does not work	Regular	4.00	<=21	Underweight	<=30 inch	>=51,000 BDT	<=20000 BDT	17.00	Low self-esteem	Very good	Neither satisfied nor dissatisfied	64.29	66.67	75.00	78.13	12.00	Poor	<4hrs
1138.00	Female	23.00	35.31	28.4	Business and Entrepreneurship	2nd year	Home	Business	Does not work	Regular	4.00	22-24	Obese	<=30 inch	<=50,000 BDT	<=20000 BDT	30.00	High self-esteem	Good	Neither satisfied nor dissatisfied	60.71	37.50	75.00	71.88	13.00	Poor	<4hrs
1139.00	Female	21.00	17.02	29.69	Business and Entrepreneurship	2nd year	Home	Service	Does not work	Regular	4.00	<=21	Underweight	<=30 inch	>=51,000 BDT	<=20000 BDT	21.00	Low self-esteem	Very good	Satisfied	60.71	70.83	75.00	78.13	14.00	Poor	5-8hrs
1140.00	Female	22.00	17.50	31.55	Business and Entrepreneurship	2nd year	Home	No response	Does not work	Prefer not to say	4.00	22-24	Underweight	>=31	>=51,000 BDT	<=20000 BDT	20.00	Low self-esteem	Good	Neither satisfied nor dissatisfied	75.00	70.83	66.67	56.25	5.00	Good	<4hrs
1141.00	Male	21.00	21.48	34.89	Science and Information Technology	2nd year	University Hall	Service	Does not work	Regular	3.00	<=21	Normal	>=31	>=51,000 BDT	>=21000 BDT	27.00	Moderate self-esteem	Neither poor nor good	Neither satisfied nor dissatisfied	64.29	66.67	83.33	71.88	5.00	Good	5-8hrs
1142.00	Female	22.00	23.78	31.71	Business and Entrepreneurship	2nd year	University Hall	Business	Does not work	Irregular	4.00	22-24	Normal	>=31	>=51,000 BDT	>=21000 BDT	18.00	Low self-esteem	Good	Satisfied	67.86	87.50	50.00	50.00	4.00	Good	>8hrs
1143.00	Female	20.00	20.16	31.51	Business and Entrepreneurship	2nd year	Home	Business	Does not work	Irregular	4.00	<=21	Normal	>=31	>=51,000 BDT	<=20000 BDT	28.00	Moderate self-esteem	Poor	Dissatisfied	17.86	12.50	0.00	18.75	10.00	Poor	5-8hrs
1144.00	Female	20.00	16.41	31.75	Business and Entrepreneurship	2nd year	Home	Business	Does not work	Regular	4.00	<=21	Underweight	>=31	>=51,000 BDT	>=21000 BDT	21.00	Low self-esteem	Neither poor nor good	Satisfied	50.00	66.67	50.00	75.00	5.00	Good	>8hrs
1145.00	Female	21.00	22.27	30.89	Business and Entrepreneurship	2nd year	Hostel	Professional	Does not work	Irregular	4.00	<=21	Normal	<=30 inch	>=51,000 BDT	>=21000 BDT	22.00	Low self-esteem	Good	Neither satisfied nor dissatisfied	85.71	54.17	58.33	59.38	4.00	Good	>8hrs
1146.00	Male	24.00	20.71	30.6	Science and Information Technology	Final year	Hostel	Business	Does not work	Irregular	3.00	22-24	Normal	<=30 inch	>=51,000 BDT	<=20000 BDT	19.00	Low self-esteem	Good	Satisfied	78.57	83.33	75.00	56.25	4.00	Good	5-8hrs
1147.00	Male	23.00	21.48	29.41	Science and Information Technology	3rd year	University Hall	Business	Part time	Irregular	1.00	22-24	Normal	<=30 inch	>=51,000 BDT	>=21000 BDT	21.00	Low self-esteem	Good	Neither satisfied nor dissatisfied	78.57	79.17	75.00	62.50	3.00	Good	5-8hrs
1148.00	Male	24.00	23.82	32.52	Science and Information Technology	3rd year	Home	Service	Does not work	Irregular	4.00	22-24	Normal	>=31	<=50,000 BDT	<=20000 BDT	17.00	Low self-esteem	Neither poor nor good	Satisfied	57.14	66.67	66.67	21.88	12.00	Poor	>8hrs
1149.00	Female	22.00	15.07	31.23	Business and Entrepreneurship	2nd year	Hostel	Business	Does not work	Prefer not to say	3.00	22-24	Underweight	>=31	<=50,000 BDT	>=21000 BDT	25.00	Low self-esteem	Neither poor nor good	Neither satisfied nor dissatisfied	64.29	45.83	66.67	50.00	9.00	Poor	5-8hrs
1150.00	Female	21.00	23.48	31.3	Business and Entrepreneurship	2nd year	Home	Business	Does not work	Regular	3.00	<=21	Normal	>=31	>=51,000 BDT	<=20000 BDT	28.00	Moderate self-esteem	Good	Neither satisfied nor dissatisfied	75.00	70.83	41.67	53.13	5.00	Good	5-8hrs

1151.00	Female	21.00	23.38	31.31	Business and Entrepreneurship	2nd year	Home	Business	Does not work	Irregular	3.00	<=21	Normal	>=31	>=51,000 BDT	<=20000 BDT	26.00	Moderate self-esteem	Neither poor nor good	Dissatisfied	46.43	37.50	50.00	46.88	10.00	Poor	5-8hrs
1152.00	Female	21.00	25.84	31.72	Business and Entrepreneurship	2nd year	Home	No response	Does not work	Irregular	4.00	<=21	Overweight	>=31	<=50,000 BDT	<=20000 BDT	20.00	Low self-esteem	Neither poor nor good	Neither satisfied nor dissatisfied	64.29	45.83	66.67	46.88	9.00	Poor	<4hrs
1153.00	Female	22.00	24.19	31.33	Business and Entrepreneurship	2nd year	Home	Business	Does not work	Irregular	2.00	22-24	Normal	>=31	>=51,000 BDT	<=20000 BDT	30.00	High self-esteem	Good	Neither satisfied nor dissatisfied	60.71	70.83	66.67	43.75	4.00	Good	>8hrs
1154.00	Female	21.00	17.36	28.39	Business and Entrepreneurship	2nd year	Hostel	Business	Does not work	Irregular	4.00	<=21	Underweight	<=30 inch	>=51,000 BDT	<=20000 BDT	22.00	Low self-esteem	Good	Very dissatisfied	53.57	37.50	75.00	62.50	9.00	Poor	<4hrs
1155.00	Female	23.00	23.14	31.5	Business and Entrepreneurship	2nd year	Home	Service	Full time	Irregular	3.00	22-24	Normal	>=31	>=51,000 BDT	<=20000 BDT	25.00	Low self-esteem	Neither poor nor good	Neither satisfied nor dissatisfied	67.86	54.17	75.00	68.75	3.00	Good	5-8hrs
1156.00	Male	25.00	29.35	31.25	Science and Information Technology	Final year	Home	No response	Full time	Regular	2.00	>=25	Overweight	>=31	>=51,000 BDT	<=20000 BDT	18.00	Low self-esteem	Neither poor nor good	Very dissatisfied	82.14	45.83	75.00	90.63	13.00	Poor	5-8hrs
1157.00	Female	24.00	26.69	31.79	Business and Entrepreneurship	Final year	Home	Business	Full time	Irregular	4.00	22-24	Overweight	>=31	>=51,000 BDT	<=20000 BDT	25.00	Low self-esteem	Neither poor nor good	Dissatisfied	78.57	45.83	75.00	71.88	11.00	Poor	>8hrs
1158.00	Female	23.00	20.18	31.48	Health and Life Sciences	3rd year	Hostel	No response	Full time	Irregular	4.00	22-24	Normal	>=31	>=51,000 BDT	<=20000 BDT	25.00	Low self-esteem	Neither poor nor good	Neither satisfied nor dissatisfied	71.43	54.17	75.00	59.38	4.00	Good	>8hrs
1159.00	Female	23.00	20.91	32.78	Health and Life Sciences	3rd year	Home	Business	Does not work	Regular	4.00	22-24	Normal	>=31	<=50,000 BDT	<=20000 BDT	23.00	Low self-esteem	Good	Neither satisfied nor dissatisfied	64.29	66.67	75.00	71.88	14.00	Poor	>8hrs
1160.00	Female	22.00	20.31	22.1	Health and Life Sciences	3rd year	Home	Service	Does not work	Irregular	4.00	22-24	Normal	<=30 inch	>=51,000 BDT	<=20000 BDT	21.00	Low self-esteem	Neither poor nor good	Satisfied	75.00	70.83	75.00	65.63	12.00	Poor	5-8hrs
1161.00	Female	23.00	23.45	38.41	Health and Life Sciences	3rd year	Home	Service	Does not work	Regular	4.00	22-24	Normal	>=31	<=50,000 BDT	<=20000 BDT	17.00	Low self-esteem	Good	Satisfied	60.71	62.50	83.33	62.50	10.00	Poor	5-8hrs
1162.00	Male	22.00	31.86	34.53	Science and Information Technology	3rd year	Home	Service	Does not work	Regular	3.00	22-24	Obese	>=31	>=51,000 BDT	>=21000 BDT	17.00	Low self-esteem	Very good	Satisfied	75.00	83.33	100.00	78.13	9.00	Poor	5-8hrs
1163.00	Male	23.00	21.56	30.09	Science and Information Technology	2nd year	Home	Service	Does not work	Irregular	3.00	22-24	Normal	<=30 inch	>=51,000 BDT	<=20000 BDT	18.00	Low self-esteem	Very good	Neither satisfied nor dissatisfied	75.00	79.17	75.00	87.50	5.00	Good	<4hrs
1164.00	Female	23.00	16.22	31.9	Health and Life Sciences	Final year	Hostel	Service	Full time	Regular	1.00	22-24	Underweight	>=31	>=51,000 BDT	<=20000 BDT	21.00	Low self-esteem	Good	Satisfied	67.86	62.50	75.00	65.63	10.00	Poor	5-8hrs
1165.00	Male	23.00	28.22	32.77	Science and Information Technology	3rd year	Hostel	No response	Full time	Irregular	4.00	22-24	Overweight	>=31	>=51,000 BDT	<=20000 BDT	17.00	Low self-esteem	Neither poor nor good	Satisfied	67.86	87.50	75.00	59.38	3.00	Good	5-8hrs
1166.00	Male	23.00	26.10	31.63	Science and Information Technology	3rd year	Hostel	Service	Does not work	Irregular	3.00	22-24	Overweight	>=31	>=51,000 BDT	<=20000 BDT	20.00	Low self-esteem	Neither poor nor good	Satisfied	71.43	66.67	75.00	50.00	11.00	Poor	>8hrs
1167.00	Male	22.00	24.21	32.37	Science and Information Technology	2nd year	Hostel	Service	Does not work	Irregular	4.00	22-24	Normal	>=31	>=51,000 BDT	<=20000 BDT	23.00	Low self-esteem	Good	Neither satisfied nor dissatisfied	67.86	54.17	41.67	50.00	5.00	Good	<4hrs
1168.00	Female	20.00	17.10	31.07	Health and Life Sciences	2nd year	Hostel	Business	Does not work	Regular	3.00	<=21	Underweight	>=31	>=51,000 BDT	>=21000 BDT	20.00	Low self-esteem	Good	Satisfied	67.86	58.33	75.00	59.38	10.00	Poor	5-8hrs
1169.00	Female	22.00	18.91	31.78	Health and Life Sciences	2nd year	University Hall	Professional	Does not work	Regular	4.00	22-24	Normal	>=31	>=51,000 BDT	<=20000 BDT	25.00	Low self-esteem	Neither poor nor good	Satisfied	60.71	62.50	66.67	65.63	10.00	Poor	<4hrs
1170.00	Female	20.00	18.75	26.8	Health and Life Sciences	2nd year	Home	Business	Does not work	Regular	3.00	<=21	Normal	<=30 inch	>=51,000 BDT	<=20000 BDT	29.00	Moderate self-esteem	Good	Satisfied	64.29	70.83	33.33	71.88	9.00	Poor	>8hrs
1171.00	Female	24.00	21.65	30.14	Health and Life Sciences	Final year	Hostel	Business	Does not work	Prefer not to say	4.00	22-24	Normal	<=30 inch	>=51,000 BDT	<=20000 BDT	14.00	Low self-esteem	Good	Satisfied	57.14	79.17	50.00	46.88	13.00	Poor	5-8hrs
1172.00	Female	22.00	18.36	31.58	Health and Life Sciences	2nd year	Home	Business	Does not work	Regular	4.00	22-24	Underweight	>=31	>=51,000 BDT	<=20000 BDT	23.00	Low self-esteem	Good	Satisfied	64.29	58.33	75.00	68.75	9.00	Poor	<4hrs
1173.00	Female	22.00	22.66	31.35	Health and Life Sciences	2nd year	Home	Service	Does not work	Regular	3.00	22-24	Normal	>=31	>=51,000 BDT	<=20000 BDT	25.00	Low self-esteem	Neither poor nor good	Satisfied	60.71	45.83	41.67	28.13	4.00	Good	<4hrs
1174.00	Female	21.00	19.35	30.53	Health and Life Sciences	1st year	Home	Service	Does not work	Regular	3.00	<=21	Normal	<=30 inch	<=50,000 BDT	<=20000 BDT	22.00	Low self-esteem	Good	Neither satisfied nor dissatisfied	71.43	50.00	66.67	65.63	5.00	Good	5-8hrs
1175.00	Female	22.00	18.91	31.66	Health and Life Sciences	2nd year	Home	Business	Full time	Regular	4.00	22-24	Normal	>=31	<=50,000 BDT	<=20000 BDT	23.00	Low self-esteem	Good	Neither satisfied nor dissatisfied	60.71	58.33	58.33	59.38	4.00	Good	>8hrs
1176.00	Female	22.00	21.36	31.86	Health and Life Sciences	2nd year	Home	Service	Does not work	Regular	2.00	22-24	Normal	>=31	>=51,000 BDT	<=20000 BDT	23.00	Low self-esteem	Good	Neither satisfied nor dissatisfied	64.29	50.00	58.33	62.50	11.00	Poor	<4hrs
1177.00	Female	21.00	22.66	31.02	Health and Life Sciences	2nd year	Hostel	No response	Does not work	Prefer not to say	4.00	<=21	Normal	>=31	>=51,000 BDT	<=20000 BDT	23.00	Low self-esteem	Good	Very satisfied	75.00	66.67	66.67	68.75	11.00	Poor	5-8hrs
1178.00	Female	20.00	24.19	31.88	Health and Life Sciences	2nd year	University Hall	Service	Does not work	Irregular	4.00	<=21	Normal	>=31	>=51,000 BDT	<=20000 BDT	23.00	Low self-esteem	Good	Satisfied	75.00	66.67	66.67	68.75	13.00	Poor	5-8hrs
1179.00	Female	21.00	19.59	26.3	Health and Life Sciences	2nd year	University Hall	Business	Full time	Irregular	4.00	<=21	Normal	<=30 inch	>=51,000 BDT	<=20000 BDT	16.00	Low self-esteem	Good	Satisfied	64.29	62.50	91.67	78.13	10.00	Poor	5-8hrs
1180.00	Female	21.00	17.58	31.37	Health and Life Sciences	2nd year	Home	Business	Does not work	Irregular	4.00	<=21	Underweight	>=31	>=51,000 BDT	<=20000 BDT	21.00	Low self-esteem	Good	Satisfied	67.86	66.67	75.00	75.00	4.00	Good	<4hrs
1181.00	Female	21.00	24.99	34.39	Health and Life Sciences	2nd year	Hostel	Business	Does not work	Regular	4.00	<=21	Overweight	>=31	>=51,000 BDT	<=20000 BDT	25.00	Low self-esteem	Good	Neither satisfied nor dissatisfied	60.71	41.67	75.00	62.50	13.00	Poor	<4hrs
1182.00	Female	21.00	22.51	31.77	Health and Life Sciences	2nd year	Home	Business	Part time	Regular	4.00	<=21	Normal	>=31	>=51,000 BDT	<=20000 BDT	20.00	Low self-esteem	Neither poor nor good	Neither satisfied nor dissatisfied	71.43	62.50	50.00	59.38	9.00	Poor	5-8hrs
1183.00	Female	23.00	18.78	31.51	Health and Life Sciences	Final year	University Hall	Service	Full time	Prefer not to say	4.00	22-24	Normal	>=31	>=51,000 BDT	<=20000 BDT	21.00	Low self-esteem	Neither poor nor good	Satisfied	85.71	70.83	58.33	65.63	9.00	Poor	5-8hrs
1184.00	Female	24.00	16.27	28.23	Health and Life Sciences	Final year	Hostel	No response	Full time	Prefer not to say	4.00	22-24	Underweight	<=30 inch	<=50,000 BDT	<=20000 BDT	28.00	Moderate self-esteem	Poor	Neither satisfied nor dissatisfied	35.71	0.00	33.33	31.25	3.00	Good	5-8hrs
1185.00	Female	22.00	17.80	30.9	Health and Life Sciences	Final year	University Hall	No response	Does not work	Prefer not to say	4.00	22-24	Underweight	<=30 inch	>=51,000 BDT	<=20000 BDT	18.00	Low self-esteem	Good	Neither satisfied nor dissatisfied	85.71	66.67	91.67	78.13	5.00	Good	<4hrs
1186.00	Female	22.00	17.26	28.88	Health and Life Sciences	Final year	Home	Professional	Does not work	Irregular	3.00	22-24	Underweight	<=30 inch	<=50,000 BDT	<=20000 BDT	20.00	Low self-esteem	Good	Satisfied	75.00	62.50	58.33	71.88	13.00	Poor	5-8hrs
1187.00	Female	21.00	14.05	30.6	Health and Life Sciences	Final year	Home	Business	Does not work	Regular	4.00	<=21	Underweight	<=30 inch	>=51,000 BDT	<=20000 BDT	20.00	Low self-esteem	Good	Satisfied	67.86	54.17	58.33	68.75	3.00	Good	<4hrs
1188.00	Male	29.00	24.59	32.06	Science and Information Technology	Final year	University Hall	Business	Does not work	Regular	3.00	>=25	Normal	>=31	<=50,000 BDT	<=20000 BDT	25.00	Low self-esteem	Good	Satisfied	71.43	70.83	58.33	56.25	10.00	Poor	<4hrs
1189.00	Female	25.00	21.88	31.37	Health and Life Sciences	Final year	Home	Service	Does not work	Irregular	4.00	>=25	Normal	>=31	<=50,000 BDT	<=20000 BDT	25.00	Low self-esteem	Neither poor nor good	Dissatisfied	57.14	50.00	41.67	40.63	3.00	Good	5-8hrs
1190.00	Female	22.00	21.09	31.79	Health and Life Sciences	2nd year	University Hall	Service	Does not work	Irregular	4.00	22-24	Normal	>=31	>=51,000 BDT	<=20000 BDT	24.00	Low self-esteem	Neither poor nor good	Neither satisfied nor dissatisfied	71.43	54.17	58.33	62.50	5.00	Good	<4hrs
1191.00	Female	22.00	21.79	31.7	Health and Life Sciences	2nd year	Hostel	Service	Does not work	Regular	4.00	22-24	Normal	>=31	>=51,000 BDT	<=20000 BDT	15.00	Low self-esteem	Neither poor nor good	Dissatisfied	67.86	75.00	58.33	71.88	4.00	Good	5-8hrs
1192.00	Female	22.00	15.13	31.17	Health and Life Sciences	2nd year	Hostel	Business	Does not work	Regular	3.00	22-24	Underweight	>=31	>=51,000 BDT	<=20000 BDT	25.00	Low self-esteem	Very good	Satisfied	71.43	62.50	75.00	50.00	3.00	Good	<4hrs
1193.00	Female	23.00	21.22	34.33	Health and Life Sciences	2nd year	University Hall	Service	Does not work	Irregular	4.00	22-24	Normal	>=31	>=51,000 BDT	<=20000 BDT	27.00	Moderate self-esteem	Good	Very dissatisfied	42.86	29.17	50.00	62.50	11.00	Poor	>8hrs
1194.00	Female	21.00	15.13	31.48	Health and Life Sciences	2nd year	Hostel	Business	Does not work	Regular	2.00	<=21	Underweight	>=31	<=50,000 BDT	<=20000 BDT	23.00	Low self-esteem	Good	Neither satisfied nor dissatisfied	53.57	58.33	58.33	53.13	11.00	Poor	5-8hrs
1195.00	Female	21.00	23.07	31.11	Health and Life Sciences	2nd year	University Hall	Service	Does not work	Regular	4.00	<=21	Normal	>=31	>=51,000 BDT	<=20000 BDT	16.00	Low self-esteem	Neither poor nor good	Dissatisfied	57.14	50.00	58.33	62.50	4.00	Good	<4hrs
1196.00	Female	23.00	20.16	31.71	Health and Life Sciences	3rd year	University Hall	Service	Does not work	Irregular	3.00	22-24	Normal	>=31	>=51,000 BDT	<=20000 BDT	22.00	Low self-esteem	Neither poor nor good	Neither satisfied nor dissatisfied	60.71	50.00	50.00	50.00	9.00	Poor	5-8hrs
1197.00	Male	21.00	20.54	31.52	Science and Information Technology	2nd year	Home	Business	Does not work	Regular	4.00	<=21	Normal	>=31	>=51,000 BDT	<=20000 BDT	19.00	Low self-esteem	Good	Satisfied	78.57	83.33	66.67	87.50	5.00	Good	5-8hrs
1198.00	Female	22.00	25.84	31.1	Health and Life Sciences	2nd year	Home	Professional	Part time	Regular	4.00	22-24	Overweight	>=31	>=51,000 BDT	<=20000 BDT	30.00	High self-esteem	Good	Satisfied	50.00	41.67	50.00	59.38	12.00	Poor	>8hrs
1199.00	Female	21.00	16.02	31.89	Health and Life Sciences	2nd year	Home	Professional	Does not work	Prefer not to say	4.00	<=21	Underweight	>=31	>=51,000 BDT	<=20000 BDT	23.00	Low self-esteem	Good	Very satisfied	67.86	62.50	50.00	59.38	5.00	Good	<4hrs
1200.00	Female	23.00	19.08	31.04	Health and Life Sciences	3rd year	University Hall	Service	Does not work	Prefer not to say	3.00	22-24	Normal	>=31	>=51,000 BDT	<=20000 BDT	21.00	Low self-esteem	Neither poor nor good	Neither satisfied nor dissatisfied	42.86	58.33	66.67	56.25	9.00	Poor	5-8hrs
1201.00	Female	22.00	19.59	31.24	Health and Life Sciences	2nd year	University Hall	Service	Does not work	Irregular	4.00	22-24	Normal	>=31	>=51,000 BDT	<=20000 BDT	24.00	Low self-esteem	Good	Neither satisfied nor dissatisfied	50.00	54.17	50.00	68.75	11.00	Poor	5-8hrs
1202.00	Female	22.00	21.88	31.5	Health and Life Sciences	2nd year	Home	Service	Does not work	Irregular	4.00	22-24	Normal	>=31	>=51,000 BDT	<=20000 BDT	23.00	Low self-esteem	Neither poor nor good	Neither satisfied nor dissatisfied	64.29	54.17	58.33	65.63	10.00	Poor	5-8hrs
1203.00	Female	21.00	19.53	31.69	Health and Life Sciences	2nd year	Home	Business	Does not work	Irregular	4.00	<=21	Normal	>=31	>=51,000 BDT	<=20000 BDT	20.00	Low self-esteem	Very good	Neither satisfied nor dissatisfied	71.43	79.17	58.33	56.25	3.00	Good	5-8hrs
1204.00	Female	22.00	22.66	31.36	Health and Life Sciences	2nd year	Hostel	Service	Does not work	Regular	4.00	22-24	Normal	>=31	>=51,000 BDT	<=20000 BDT	26.00	Moderate self-esteem	Neither poor nor good	Neither satisfied nor dissatisfied	46.43	16.67	16.67	40.63	4.00	Good	5-8hrs
1205.00	Female	21.00	29.28	31.72	Health and Life Sciences	2nd year	University Hall	No response	Does not work	Irregular	4.00	<=21	Overweight	>=31	>=51,000 BDT	<=20000 BDT	16.00	Low self-esteem	Good	Neither satisfied nor dissatisfied	64.29	70.83	50.00	50.00	9.00	Poor	5-8hrs
1206.00	Female	22.00	23.34	31.3	Health and Life Sciences	2nd year	Hostel	Service	Does not work	Regular	4.00	22-24	Normal	>=31	>=51,000 BDT	<=20000 BDT	24.00	Low self-esteem	Good	Dissatisfied	42.86	37.50	50.00	18.75	5.00	Good	5-8hrs
1207.00	Female	22.00	19.67	31.22	Health and Life Sciences	2nd year	Home	Business	Does not work	Irregular	4.00	22-24	Normal	>=31	>=51,000 BDT	<=20000 BDT	27.00	Moderate self-esteem	Good	Very dissatisfied	32.14	54.17	41.67	59.38	5.00	Good	<4hrs
1208.00	Female	23.00	24.17	31.89	Health and Life Sciences	Final year	Home	Business	Does not work	Irregular	4.00	22-24	Normal	>=31	>=51,000 BDT	<=20000 BDT	18.00	Low self-esteem	Good	Satisfied	75.00	66.67	66.67	65.63	3.00	Good	5-8hrs
1209.00	Female	21.00	20.31	30.7	Health and Life Sciences	2nd year	Home	Service	Does not work	Irregular	3.00	<=21	Normal	<=30 inch	>=51,000 BDT	<=20000 BDT	19.00	Low self-esteem	Neither poor nor good	Satisfied	67.86	50.00	41.67	56.25	9.00	Poor	5-8hrs
1210.00	Female	22.00	15.51	28.88	Health and Life Sciences	2nd year	Home	Business	Does not work	Regular	4.00	22-24	Underweight	<=30 inch	>=51,000 BDT	<=20000 BDT	21.00	Low self-esteem	Neither poor nor good	Very dissatisfied	32.14	29.17	75.00	53.13	9.00	Poor	5-8hrs
1211.00	Male	23.00	23.86	34.62	Science and Information Technology	2nd year	University Hall	Business	Does not work	Irregular	4.00	22-24	Normal	>=31	<=50,000 BDT	<=20000 BDT	19.00	Low self-esteem	Neither poor nor good	Satisfied	64.29	50.00	66.67	62.50	11.00	Poor	5-8hrs
1212.00	Female	22.00	18.78	31.71	Health and Life Sciences	2nd year	Hostel	Business	Does not work	Regular	4.00	22-24	Normal	>=31	<=50,000 BDT	<=20000 BDT	31.00	High self-esteem	Very poor	Dissatisfied	42.86	45.83	58.33	56.25	11.00	Poor	5-8hrs
1213.00	Male	22.00	27.51	35.3	Science and Information Technology	2nd year	University Hall	Business	Does not work	Regular	3.00	22-24	Overweight	>=31	<=50,000 BDT	<=20000 BDT	26.00	Moderate self-esteem	Neither poor nor good	Neither satisfied nor dissatisfied	60.71	45.83	66.67	40.63	9.00	Poor	>8hrs
1214.00	Female	22.00	19.58	31.78	Health and Life Sciences	2nd year	Hostel	Business	Does not work	Regular	4.00	22-24	Normal	>=31	<=50,000 BDT	<=20000 BDT	23.00	Low self-esteem	Poor	Neither satisfied nor dissatisfied	64.29	62.50	58.33	65.63	12.00	Poor	5-8hrs
1215.00	Male	22.00	26.78	34.48	Science and Information Technology	2nd year	Hostel	Business	Does not work	Irregular	4.00	22-24	Overweight	>=31	>=51,000 BDT	<=20000 BDT	24.00	Low self-esteem	Good	Satisfied	82.14	75.00	75.00	53.13	5.00	Good	5-8hrs
1216.00	Female	23.00	17.43	31.31	Health and Life Sciences	2nd year	University Hall	Business	Does not work	Irregular	3.00	22-24	Underweight	>=31	>=51,000 BDT	<=20000 BDT	21.00	Low self-esteem	Good	Neither satisfied nor dissatisfied	75.00	62.50	66.67	53.13	5.00	Good	<4hrs
1217.00	Male	23.00	23.14	34.71	Science and Information Technology	2nd year	Hostel	Business	Does not work	Irregular	4.00	22-24	Normal	>=31	>=51,000 BDT	<=20000 BDT	33.00	High self-esteem	Neither poor nor good	Neither satisfied nor dissatisfied	64.29	20.83	33.33	62.50	10.00	Poor	>8hrs
1218.00	Female	22.00	20.02	31.07	Health and Life Sciences	3rd year	Hostel	Business	Does not work	Irregular	4.00	22-24	Normal	>=31	>=51,000 BDT	<=20000 BDT	30.00	High self-esteem	Very poor	Dissatisfied	50.00	29.17	33.33	31.25	4.00	Good	5-8hrs
1219.00	Male	28.00	25.72	33.28	Science and Information Technology	2nd year	Hostel	Service	Does not work	Irregular	4.00	>=25	Overweight	>=31	<=50,000 BDT	<=20000 BDT	22.00	Low self-esteem	Neither poor nor good	Very dissatisfied	78.57	54.17	66.67	40.63	5.00	Good	>8hrs
1220.00	Female	21.00	23.14	31.11	Health and Life Sciences	2nd year	Home	No response	Does not work	Prefer not to say	3.00	<=21	Normal	>=31	>=51,000 BDT	<=20000 BDT	20.00	Low self-esteem	Neither poor nor good	Satisfied	64.29	62.50	66.67	59.38	4.00	Good	<4hrs
1221.00	Female	22.00	20.02	31.54	Health and Life Sciences	2nd year	Hostel	Service	Does not work	Irregular	4.00	22-24	Normal	>=31	>=51,000 BDT	<=20000 BDT	24.00	Low self-esteem	Good	Satisfied	67.86	54.17	25.00	50.00	5.00	Good	5-8hrs
1222.00	Female	21.00	20.65	31.66	Health and Life Sciences	2nd year	Hostel	Professional	Does not work	Prefer not to say	4.00	<=21	Normal	>=31	>=51,000 BDT	<=20000 BDT	29.00	Moderate self-esteem	Very poor	Neither satisfied nor dissatisfied	46.43	37.50	58.33	25.00	9.00	Poor	5-8hrs
1223.00	Male	22.00	20.16	30.84	Science and Information Technology	2nd year	Home	Professional	Does not work	Prefer not to say	4.00	22-24	Normal	<=30 inch	<=50,000 BDT	<=20000 BDT	17.00	Low self-esteem	Neither poor nor good	Neither satisfied nor dissatisfied	75.00	83.33	58.33	34.38	3.00	Good	5-8hrs
1224.00	Male	23.00	26.26	31.01	Science and Information Technology	2nd year	Home	Service	Does not work	Prefer not to say	3.00	22-24	Overweight	>=31	>=51,000 BDT	<=20000 BDT	23.00	Low self-esteem	Neither poor nor good	Satisfied	57.14	54.17	50.00	34.38	10.00	Poor	5-8hrs
1225.00	Female	24.00	20.16	27.68	Health and Life Sciences	2nd year	Home	Service	Does not work	Irregular	4.00	22-24	Normal	<=30 inch	<=50,000 BDT	<=20000 BDT	31.00	High self-esteem	Very poor	Satisfied	89.29	37.50	75.00	56.25	5.00	Good	>8hrs
1226.00	Female	23.00	25.01	30.9	Health and Life Sciences	2nd year	Home	No response	Does not work	Irregular	4.00	22-24	Overweight	<=30 inch	<=50,000 BDT	<=20000 BDT	28.00	Moderate self-esteem	Very poor	Neither satisfied nor dissatisfied	67.86	25.00	66.67	53.13	5.00	Good	5-8hrs
1227.00	Female	22.00	18.44	26.69	Health and Life Sciences	2nd year	Hostel	Business	Does not work	Regular	4.00	22-24	Underweight	<=30 inch	>=51,000 BDT	<=20000 BDT	22.00	Low self-esteem	Very poor	Neither satisfied nor dissatisfied	46.43	20.83	33.33	31.25	12.00	Poor	<4hrs
1228.00	Female	20.00	18.87	31.13	Health and Life Sciences	1st year	Hostel	No response	Does not work	Irregular	4.00	<=21	Normal	>=31	>=51,000 BDT	<=20000 BDT	25.00	Low self-esteem	Neither poor nor good	Neither satisfied nor dissatisfied	53.57	45.83	50.00	46.88	10.00	Poor	5-8hrs

1229.00	Female	19.00	24.92	34.23	Health and Life Sciences	1st year	University Hall	Service	Does not work	Prefer not to say	3.00	<=21	Overweight	>=31	>=51,000 BDT	<=20000 BDT	28.00	Moderate self-esteem	Neither poor nor good	Satisfied	53.57	58.33	75.00	37.50	4.00	Good	5-8hrs
1230.00	Female	21.00	15.53	31.98	Health and Life Sciences	1st year	University Hall	No response	Does not work	Prefer not to say	4.00	<=21	Underweight	>=31	>=51,000 BDT	<=20000 BDT	22.00	Low self-esteem	Good	Neither satisfied nor dissatisfied	53.57	58.33	58.33	53.13	5.00	Good	5-8hrs
1231.00	Female	20.00	17.04	31.94	Health and Life Sciences	1st year	Hostel	Service	Part time	Irregular	4.00	<=21	Underweight	>=31	>=51,000 BDT	<=20000 BDT	26.00	Moderate self-esteem	Good	Neither satisfied nor dissatisfied	67.86	45.83	66.67	53.13	5.00	Good	5-8hrs
1232.00	Male	24.00	14.23	31.48	Science and Information Technology	2nd year	University Hall	Business	Full time	Regular	3.00	22-24	Underweight	>=31	<=50,000 BDT	<=20000 BDT	24.00	Low self-esteem	Good	Satisfied	67.86	58.33	75.00	71.88	12.00	Poor	5-8hrs
1233.00	Female	22.00	23.14	31.58	Health and Life Sciences	2nd year	Home	Service	Does not work	Prefer not to say	3.00	22-24	Normal	>=31	>=51,000 BDT	<=20000 BDT	24.00	Low self-esteem	Good	Neither satisfied nor dissatisfied	39.29	58.33	41.67	56.25	4.00	Good	5-8hrs
1234.00	Female	21.00	22.82	28.19	Health and Life Sciences	2nd year	Home	No response	Full time	Regular	4.00	<=21	Normal	<=30 inch	<=50,000 BDT	<=20000 BDT	25.00	Low self-esteem	Good	Satisfied	67.86	58.33	66.67	53.13	11.00	Poor	<4hrs
1235.00	Female	21.00	21.21	26.89	Health and Life Sciences	2nd year	Home	No response	Does not work	Regular	4.00	<=21	Normal	<=30 inch	<=50,000 BDT	<=20000 BDT	25.00	Low self-esteem	Good	Dissatisfied	60.71	66.67	75.00	71.88	11.00	Poor	<4hrs
1236.00	Female	23.00	21.79	31.27	Health and Life Sciences	2nd year	Home	Service	Does not work	Irregular	4.00	22-24	Normal	>=31	>=51,000 BDT	<=20000 BDT	29.00	Moderate self-esteem	Neither poor nor good	Very dissatisfied	60.71	29.17	50.00	43.75	5.00	Good	<4hrs
1237.00	Female	22.00	14.42	28.44	Health and Life Sciences	2nd year	Home	Professional	Does not work	Regular	4.00	22-24	Underweight	<=30 inch	>=51,000 BDT	<=20000 BDT	21.00	Low self-esteem	Good	Dissatisfied	64.29	54.17	75.00	59.38	9.00	Poor	<4hrs
1238.00	Male	22.00	24.11	30.23	Science and Information Technology	2nd year	Hostel	Business	Does not work	Irregular	3.00	22-24	Normal	<=30 inch	>=51,000 BDT	<=20000 BDT	26.00	Moderate self-esteem	Good	Neither satisfied nor dissatisfied	50.00	54.17	66.67	62.50	4.00	Good	5-8hrs
1239.00	Male	21.00	29.02	31.58	Science and Information Technology	2nd year	Home	Service	Does not work	Regular	3.00	<=21	Overweight	>=31	<=50,000 BDT	<=20000 BDT	16.00	Low self-esteem	Good	Neither satisfied nor dissatisfied	60.71	66.67	50.00	75.00	5.00	Good	5-8hrs
1240.00	Female	22.00	28.22	34.6	Health and Life Sciences	2nd year	Home	Service	Does not work	Regular	4.00	22-24	Overweight	>=31	>=51,000 BDT	<=20000 BDT	25.00	Low self-esteem	Neither poor nor good	Satisfied	60.71	45.83	58.33	56.25	9.00	Poor	<4hrs
1241.00	Female	24.00	22.27	30.26	Health and Life Sciences	3rd year	Hostel	Business	Part time	Irregular	4.00	22-24	Normal	<=30 inch	<=50,000 BDT	<=20000 BDT	21.00	Low self-esteem	Good	Dissatisfied	53.57	50.00	58.33	28.13	9.00	Poor	5-8hrs
1242.00	Male	28.00	28.13	31.66	Science and Information Technology	Final year	Hostel	Business	Does not work	Irregular	1.00	>=25	Overweight	>=31	>=51,000 BDT	<=20000 BDT	22.00	Low self-esteem	Neither poor nor good	Dissatisfied	64.29	41.67	50.00	28.13	11.00	Poor	5-8hrs
1243.00	Male	21.00	27.34	31.76	Science and Information Technology	2nd year	Home	Service	Does not work	Regular	4.00	<=21	Overweight	>=31	<=50,000 BDT	<=20000 BDT	29.00	Moderate self-esteem	Poor	Neither satisfied nor dissatisfied	35.71	29.17	16.67	21.88	10.00	Poor	<4hrs
1244.00	Female	23.00	19.58	31.38	Health and Life Sciences	2nd year	Hostel	Service	Full time	Irregular	4.00	22-24	Normal	>=31	>=51,000 BDT	<=20000 BDT	25.00	Low self-esteem	Neither poor nor good	Dissatisfied	53.57	50.00	33.33	53.13	5.00	Good	<4hrs
1245.00	Female	22.00	21.94	30.83	Health and Life Sciences	2nd year	Home	Business	Does not work	Regular	4.00	22-24	Normal	<=30 inch	>=51,000 BDT	<=20000 BDT	14.00	Low self-esteem	Very good	Very satisfied	67.86	87.50	83.33	71.88	3.00	Good	5-8hrs
1246.00	Female	22.00	17.53	31.87	Health and Life Sciences	2nd year	Hostel	Service	Full time	Regular	4.00	22-24	Underweight	>=31	>=51,000 BDT	<=20000 BDT	28.00	Moderate self-esteem	Neither poor nor good	Neither satisfied nor dissatisfied	53.57	41.67	50.00	65.63	10.00	Poor	>8hrs
1247.00	Female	20.00	20.05	31.53	Health and Life Sciences	1st year	Home	Business	Does not work	Prefer not to say	4.00	<=21	Normal	>=31	>=51,000 BDT	<=20000 BDT	24.00	Low self-esteem	Neither poor nor good	Satisfied	64.29	54.17	50.00	53.13	5.00	Good	5-8hrs
1248.00	Female	20.00	17.40	28.1	Health and Life Sciences	1st year	Home	Service	Full time	Irregular	4.00	<=21	Underweight	<=30 inch	>=51,000 BDT	<=20000 BDT	29.00	Moderate self-esteem	Poor	Dissatisfied	46.43	16.67	25.00	43.75	12.00	Poor	5-8hrs
1249.00	Female	19.00	24.17	31.28	Health and Life Sciences	1st year	Home	Service	Does not work	Regular	1.00	<=21	Normal	>=31	>=51,000 BDT	<=20000 BDT	17.00	Low self-esteem	Neither poor nor good	Dissatisfied	67.86	58.33	75.00	65.63	10.00	Poor	5-8hrs
1250.00	Female	22.00	19.14	31.68	Health and Life Sciences	1st year	Hostel	Service	Full time	Irregular	4.00	22-24	Normal	>=31	>=51,000 BDT	<=20000 BDT	31.00	High self-esteem	Neither poor nor good	Dissatisfied	32.14	16.67	50.00	50.00	13.00	Poor	5-8hrs
1251.00	Female	20.00	17.09	31.36	Health and Life Sciences	1st year	Home	Service	Does not work	Regular	4.00	<=21	Underweight	>=31	>=51,000 BDT	<=20000 BDT	21.00	Low self-esteem	Neither poor nor good	Neither satisfied nor dissatisfied	57.14	58.33	50.00	65.63	5.00	Good	5-8hrs
1252.00	Female	20.00	21.96	31.44	Health and Life Sciences	1st year	Home	Professional	Does not work	Irregular	4.00	<=21	Normal	>=31	>=51,000 BDT	<=20000 BDT	25.00	Low self-esteem	Good	Neither satisfied nor dissatisfied	57.14	70.83	75.00	59.38	3.00	Good	5-8hrs
1253.00	Female	21.00	18.53	28.42	Health and Life Sciences	2nd year	Hostel	Service	Does not work	Regular	4.00	<=21	Normal	<=30 inch	<=50,000 BDT	<=20000 BDT	17.00	Low self-esteem	Neither poor nor good	Dissatisfied	53.57	33.33	58.33	18.75	13.00	Poor	>8hrs
1254.00	Female	21.00	26.20	31.95	Health and Life Sciences	2nd year	University Hall	No response	Does not work	Prefer not to say	4.00	<=21	Overweight	>=31	<=50,000 BDT	<=20000 BDT	25.00	Low self-esteem	Good	Neither satisfied nor dissatisfied	57.14	58.33	75.00	59.38	10.00	Poor	5-8hrs
1255.00	Female	22.00	24.17	31.32	Health and Life Sciences	2nd year	Home	Business	Does not work	Irregular	4.00	22-24	Normal	>=31	<=50,000 BDT	<=20000 BDT	10.00	Low self-esteem	Good	Very dissatisfied	67.86	79.17	66.67	56.25	5.00	Good	>8hrs
1256.00	Female	21.00	28.34	31.71	Health and Life Sciences	2nd year	University Hall	Service	Does not work	Regular	4.00	<=21	Overweight	>=31	>=51,000 BDT	<=20000 BDT	27.00	Moderate self-esteem	Neither poor nor good	Dissatisfied	53.57	54.17	58.33	53.13	11.00	Poor	5-8hrs
1257.00	Female	21.00	24.21	31.3	Health and Life Sciences	2nd year	University Hall	Professional	Does not work	Regular	3.00	<=21	Normal	>=31	<=50,000 BDT	<=20000 BDT	26.00	Moderate self-esteem	Neither poor nor good	Neither satisfied nor dissatisfied	53.57	45.83	66.67	46.88	5.00	Good	5-8hrs
1258.00	Female	21.00	18.03	28.46	Health and Life Sciences	2nd year	Home	Professional	Full time	Prefer not to say	2.00	<=21	Underweight	<=30 inch	<=50,000 BDT	<=20000 BDT	34.00	High self-esteem	Good	Satisfied	67.86	41.67	50.00	40.63	4.00	Good	<4hrs
1259.00	Female	22.00	20.88	31.74	Health and Life Sciences	2nd year	Hostel	Service	Full time	Regular	4.00	22-24	Normal	>=31	<=50,000 BDT	<=20000 BDT	24.00	Low self-esteem	Neither poor nor good	Very dissatisfied	46.43	45.83	41.67	40.63	4.00	Good	<4hrs
1260.00	Female	21.00	20.65	31.8	Health and Life Sciences	2nd year	Home	Service	Does not work	Irregular	1.00	<=21	Normal	>=31	>=51,000 BDT	<=20000 BDT	20.00	Low self-esteem	Good	Satisfied	57.14	58.33	58.33	59.38	5.00	Good	>8hrs
1261.00	Female	22.00	24.19	28.12	Health and Life Sciences	2nd year	University Hall	Business	Full time	Regular	4.00	22-24	Normal	<=30 inch	>=51,000 BDT	<=20000 BDT	26.00	Moderate self-esteem	Neither poor nor good	Very dissatisfied	53.57	37.50	50.00	46.88	5.00	Good	5-8hrs
1262.00	Female	21.00	23.85	31.32	Health and Life Sciences	2nd year	Home	Service	Does not work	Regular	4.00	<=21	Normal	>=31	<=50,000 BDT	<=20000 BDT	28.00	Moderate self-esteem	Neither poor nor good	Dissatisfied	42.86	41.67	50.00	40.63	10.00	Poor	>8hrs
1263.00	Female	22.00	25.39	30.13	Health and Life Sciences	2nd year	University Hall	Business	Does not work	Irregular	4.00	22-24	Overweight	<=30 inch	>=51,000 BDT	<=20000 BDT	28.00	Moderate self-esteem	Neither poor nor good	Dissatisfied	46.43	62.50	75.00	31.25	11.00	Poor	>8hrs
1264.00	Female	22.00	24.19	31.44	Health and Life Sciences	2nd year	Home	Business	Does not work	Irregular	3.00	22-24	Normal	>=31	>=51,000 BDT	<=20000 BDT	26.00	Moderate self-esteem	Neither poor nor good	Dissatisfied	32.14	37.50	41.67	56.25	5.00	Good	5-8hrs
1265.00	Female	21.00	16.93	28.25	Health and Life Sciences	2nd year	Home	Business	Does not work	Prefer not to say	4.00	<=21	Underweight	<=30 inch	>=51,000 BDT	<=20000 BDT	27.00	Moderate self-esteem	Good	Satisfied	50.00	41.67	41.67	56.25	14.00	Poor	5-8hrs
1266.00	Female	20.00	16.02	31.67	Health and Life Sciences	2nd year	University Hall	Business	Does not work	Regular	3.00	<=21	Underweight	>=31	>=51,000 BDT	<=20000 BDT	20.00	Low self-esteem	Neither poor nor good	Neither satisfied nor dissatisfied	46.43	50.00	66.67	37.50	10.00	Poor	<4hrs
1267.00	Female	21.00	22.98	31.26	Health and Life Sciences	2nd year	Home	No response	Full time	Prefer not to say	4.00	<=21	Normal	>=31	>=51,000 BDT	<=20000 BDT	26.00	Moderate self-esteem	Good	Neither satisfied nor dissatisfied	57.14	37.50	50.00	43.75	10.00	Poor	5-8hrs
1268.00	Female	21.00	17.61	28.19	Health and Life Sciences	2nd year	University Hall	Business	Part time	Prefer not to say	2.00	<=21	Underweight	<=30 inch	>=51,000 BDT	<=20000 BDT	20.00	Low self-esteem	Good	Neither satisfied nor dissatisfied	71.43	79.17	66.67	53.13	11.00	Poor	5-8hrs
1269.00	Female	19.00	22.01	31.61	Health and Life Sciences	1st year	Home	Service	Does not work	Regular	2.00	<=21	Normal	>=31	>=51,000 BDT	<=20000 BDT	26.00	Moderate self-esteem	Neither poor nor good	Very satisfied	57.14	83.33	75.00	59.38	11.00	Poor	>8hrs
1270.00	Female	19.00	23.38	31.67	Health and Life Sciences	1st year	Hostel	Business	Full time	Prefer not to say	3.00	<=21	Normal	>=31	>=51,000 BDT	<=20000 BDT	31.00	High self-esteem	Neither poor nor good	Neither satisfied nor dissatisfied	53.57	50.00	50.00	59.38	3.00	Good	5-8hrs
1271.00	Female	19.00	17.02	31.59	Health and Life Sciences	1st year	Home	Service	Does not work	Prefer not to say	4.00	<=21	Underweight	>=31	<=50,000 BDT	<=20000 BDT	25.00	Low self-esteem	Neither poor nor good	Neither satisfied nor dissatisfied	53.57	58.33	66.67	43.75	3.00	Good	<4hrs
1272.00	Female	20.00	23.25	31.74	Health and Life Sciences	1st year	University Hall	Professional	Does not work	Regular	4.00	<=21	Normal	>=31	<=50,000 BDT	<=20000 BDT	27.00	Moderate self-esteem	Good	Satisfied	67.86	41.67	75.00	59.38	5.00	Good	>8hrs
1273.00	Female	20.00	21.18	30.8	Health and Life Sciences	1st year	Hostel	Business	Does not work	Prefer not to say	4.00	<=21	Normal	<=30 inch	>=51,000 BDT	<=20000 BDT	24.00	Low self-esteem	Neither poor nor good	Neither satisfied nor dissatisfied	60.71	62.50	66.67	43.75	5.00	Good	5-8hrs
1274.00	Female	23.00	13.81	31.09	Health and Life Sciences	2nd year	University Hall	Business	Full time	Irregular	4.00	22-24	Underweight	>=31	<=50,000 BDT	<=20000 BDT	21.00	Low self-esteem	Neither poor nor good	Dissatisfied	64.29	50.00	75.00	59.38	14.00	Poor	<4hrs
1275.00	Female	21.00	21.48	30.68	Health and Life Sciences	2nd year	Hostel	Service	Does not work	Irregular	3.00	<=21	Normal	<=30 inch	>=51,000 BDT	<=20000 BDT	22.00	Low self-esteem	Good	Neither satisfied nor dissatisfied	71.43	54.17	50.00	56.25	4.00	Good	<4hrs
1276.00	Female	21.00	21.88	28.33	Health and Life Sciences	2nd year	Hostel	Business	Does not work	Regular	3.00	<=21	Normal	<=30 inch	>=51,000 BDT	<=20000 BDT	24.00	Low self-esteem	Good	Neither satisfied nor dissatisfied	60.71	54.17	75.00	59.38	12.00	Poor	<4hrs
1277.00	Female	22.00	18.91	31.96	Health and Life Sciences	2nd year	Home	Business	Part time	Regular	4.00	22-24	Normal	>=31	<=50,000 BDT	<=20000 BDT	24.00	Low self-esteem	Good	Satisfied	75.00	87.50	66.67	81.25	5.00	Good	5-8hrs
1278.00	Female	22.00	18.51	31.69	Health and Life Sciences	2nd year	University Hall	Professional	Does not work	Regular	1.00	22-24	Normal	>=31	>=51,000 BDT	<=20000 BDT	20.00	Low self-esteem	Good	Dissatisfied	53.57	62.50	66.67	65.63	10.00	Poor	<4hrs
1279.00	Female	23.00	17.61	28.61	Health and Life Sciences	2nd year	University Hall	Service	Does not work	Irregular	4.00	22-24	Underweight	<=30 inch	>=51,000 BDT	<=20000 BDT	29.00	Moderate self-esteem	Poor	Dissatisfied	46.43	58.33	33.33	21.88	14.00	Poor	5-8hrs
1280.00	Female	21.00	22.75	32.35	Health and Life Sciences	2nd year	Home	Business	Does not work	Regular	4.00	<=21	Normal	>=31	>=51,000 BDT	<=20000 BDT	29.00	Moderate self-esteem	Very poor	Satisfied	71.43	29.17	41.67	56.25	5.00	Good	5-8hrs
1281.00	Female	22.00	19.75	28.99	Health and Life Sciences	2nd year	University Hall	Business	Does not work	Regular	4.00	22-24	Normal	<=30 inch	>=51,000 BDT	<=20000 BDT	15.00	Low self-esteem	Neither poor nor good	Satisfied	92.86	83.33	58.33	71.88	11.00	Poor	5-8hrs
1282.00	Male	20.00	18.99	30.61	Science and Information Technology	1st year	Hostel	Service	Does not work	Irregular	1.00	<=21	Normal	<=30 inch	>=51,000 BDT	<=20000 BDT	30.00	High self-esteem	Neither poor nor good	Dissatisfied	75.00	33.33	66.67	56.25	4.00	Good	5-8hrs
1283.00	Female	20.00	23.38	31.83	Health and Life Sciences	1st year	Hostel	Business	Does not work	Regular	4.00	<=21	Normal	>=31	<=50,000 BDT	<=20000 BDT	25.00	Low self-esteem	Neither poor nor good	Neither satisfied nor dissatisfied	53.57	50.00	66.67	56.25	5.00	Good	>8hrs
1284.00	Female	20.00	12.86	31.26	Humanities and Social Sciences	1st year	Hostel	Business	Does not work	Regular	3.00	<=21	Underweight	>=31	>=51,000 BDT	<=20000 BDT	22.00	Low self-esteem	Good	Satisfied	57.14	58.33	58.33	59.38	12.00	Poor	5-8hrs
1285.00	Male	20.00	23.45	31.25	Science and Information Technology	1st year	Hostel	Professional	Does not work	Irregular	4.00	<=21	Normal	>=31	<=50,000 BDT	<=20000 BDT	24.00	Low self-esteem	Neither poor nor good	Dissatisfied	57.14	58.33	58.33	40.63	9.00	Poor	5-8hrs
1286.00	Female	19.00	20.45	31.5	Humanities and Social Sciences	1st year	Home	Business	Does not work	Regular	3.00	<=21	Normal	>=31	>=51,000 BDT	<=20000 BDT	21.00	Low self-esteem	Very good	Satisfied	60.71	58.33	66.67	84.38	9.00	Poor	<4hrs
1287.00	Female	19.00	19.67	31.26	Humanities and Social Sciences	1st year	Home	Business	Does not work	Regular	3.00	<=21	Normal	>=31	>=51,000 BDT	<=20000 BDT	19.00	Low self-esteem	Good	Satisfied	50.00	58.33	50.00	75.00	9.00	Poor	5-8hrs
1288.00	Female	19.00	26.17	31.97	Humanities and Social Sciences	1st year	Home	Service	Does not work	Prefer not to say	4.00	<=21	Overweight	>=31	>=51,000 BDT	<=20000 BDT	25.00	Low self-esteem	Good	Very satisfied	46.43	41.67	75.00	59.38	9.00	Poor	5-8hrs
1289.00	Female	20.00	20.18	31.63	Humanities and Social Sciences	1st year	Home	No response	Part time	Irregular	4.00	<=21	Normal	>=31	>=51,000 BDT	<=20000 BDT	18.00	Low self-esteem	Good	Satisfied	71.43	66.67	83.33	50.00	12.00	Poor	5-8hrs
1290.00	Female	19.00	23.44	31.13	Humanities and Social Sciences	1st year	Home	Business	Does not work	Prefer not to say	4.00	<=21	Normal	>=31	>=51,000 BDT	<=20000 BDT	26.00	Moderate self-esteem	Neither poor nor good	Very dissatisfied	42.86	45.83	83.33	46.88	10.00	Poor	5-8hrs
1291.00	Female	20.00	19.92	31.21	Humanities and Social Sciences	1st year	Home	Business	Does not work	Prefer not to say	4.00	<=21	Normal	>=31	>=51,000 BDT	<=20000 BDT	20.00	Low self-esteem	Neither poor nor good	Very satisfied	60.71	50.00	50.00	34.38	9.00	Poor	5-8hrs
1292.00	Male	24.00	28.22	31.25	Science and Information Technology	3rd year	Hostel	Professional	Does not work	Irregular	4.00	22-24	Overweight	>=31	>=51,000 BDT	<=20000 BDT	21.00	Low self-esteem	Neither poor nor good	Satisfied	75.00	70.83	58.33	71.88	9.00	Poor	5-8hrs
1293.00	Male	22.00	24.59	31.32	Science and Information Technology	2nd year	Hostel	Service	Does not work	Irregular	4.00	22-24	Normal	>=31	>=51,000 BDT	<=20000 BDT	20.00	Low self-esteem	Neither poor nor good	Neither satisfied nor dissatisfied	71.43	79.17	75.00	75.00	5.00	Good	5-8hrs
1294.00	Male	23.00	21.36	31.44	Science and Information Technology	2nd year	Hostel	Business	Does not work	Regular	4.00	22-24	Normal	>=31	>=51,000 BDT	<=20000 BDT	21.00	Low self-esteem	Good	Satisfied	64.29	70.83	66.67	75.00	3.00	Good	5-8hrs
1295.00	Female	21.00	25.00	31.83	Humanities and Social Sciences	1st year	Hostel	Service	Does not work	Irregular	4.00	<=21	Overweight	>=31	>=51,000 BDT	<=20000 BDT	21.00	Low self-esteem	Neither poor nor good	Neither satisfied nor dissatisfied	39.29	54.17	58.33	68.75	9.00	Poor	5-8hrs
1296.00	Female	21.00	25.83	31.73	Humanities and Social Sciences	2nd year	Relatives house	Professional	Full time	Prefer not to say	4.00	<=21	Overweight	>=31	<=50,000 BDT	<=20000 BDT	22.00	Low self-esteem	Neither poor nor good	Dissatisfied	57.14	41.67	66.67	59.38	9.00	Poor	<4hrs
1297.00	Female	21.00	20.16	31.23	Humanities and Social Sciences	2nd year	Home	No response	Does not work	Regular	4.00	<=21	Normal	>=31	>=51,000 BDT	<=20000 BDT	22.00	Low self-esteem	Neither poor nor good	Very dissatisfied	64.29	50.00	58.33	50.00	9.00	Poor	5-8hrs
1298.00	Female	21.00	24.17	31.21	Humanities and Social Sciences	2nd year	Relatives house	Business	Does not work	Prefer not to say	3.00	<=21	Normal	>=31	>=51,000 BDT	<=20000 BDT	22.00	Low self-esteem	Good	Satisfied	75.00	62.50	75.00	71.88	3.00	Good	<4hrs
1299.00	Male	22.00	23.44	30.17	Science and Information Technology	2nd year	Hostel	Business	Does not work	Irregular	3.00	22-24	Normal	<=30 inch	>=51,000 BDT	<=20000 BDT	23.00	Low self-esteem	Good	Satisfied	60.71	66.67	91.67	56.25	15.00	Poor	>8hrs
1300.00	Male	23.00	24.99	30.95	Science and Information Technology	2nd year	Home	Business	Does not work	Irregular	4.00	22-24	Overweight	<=30 inch	<=50,000 BDT	<=20000 BDT	25.00	Low self-esteem	Good	Satisfied	50.00	54.17	75.00	56.25	15.00	Poor	>8hrs
1301.00	Female	22.00	19.59	28.41	Humanities and Social Sciences	Final year	Hostel	Service	Full time	Prefer not to say	4.00	22-24	Normal	<=30 inch	<=50,000 BDT	<=20000 BDT	23.00	Low self-esteem	Good	Very satisfied	60.71	66.67	58.33	56.25	10.00	Poor	<4hrs
1302.00	Female	22.00	22.80	38.98	Humanities and Social Sciences	Final year	Home	Service	Does not work	Prefer not to say	4.00	22-24	Normal	>=31	>=51,000 BDT	<=20000 BDT	23.00	Low self-esteem	Good	Satisfied	82.14	62.50	50.00	65.63	11.00	Poor	5-8hrs
1303.00	Female	22.00	19.52	36.68	Humanities and Social Sciences	Final year	Home	Service	Does not work	Regular	4.00	22-24	Normal	>=31	<=50,000 BDT	<=20000 BDT	25.00	Low self-esteem	Neither poor nor good	Neither satisfied nor dissatisfied	57.14	50.00	75.00	50.00	5.00	Good	5-8hrs
1304.00	Female	21.00	24.92	31.88	Humanities and Social Sciences	2nd year	University Hall	Business	Does not work	Irregular	4.00	<=21	Overweight	>=31	>=51,000 BDT	>=21000 BDT	23.00	Low self-esteem	Neither poor nor good	Satisfied	82.14	66.67	75.00	96.88	4.00	Good	>8hrs
1305.00	Female	21.00	16.92	31.15	Humanities and Social Sciences	2nd year	Hostel	Business	Does not work	Irregular	4.00	<=21	Underweight	>=31	<=50,000 BDT	<=20000 BDT	26.00	Moderate self-esteem	Good	Satisfied	71.43	66.67	50.00	62.50	9.00	Poor	<4hrs
1306.00	Female	21.00	14.65	30.24	Humanities and Social Sciences	2nd year	Home	Business	Does not work	Regular	4.00	<=21	Underweight	<=30 inch	>=51,000 BDT	<=20000 BDT	25.00	Low self-esteem	Good	Satisfied	64.29	66.67	58.33	68.75	12.00	Poor	<4hrs

1307.00	Male	23.00	27.61	31.44	Science and Information Technology	2nd year	University Hall	Service	Full time	Irregular	4.00	22-24	Overweight	>=31	<=50,000 BDT	<=20000 BDT	30.00	High self-esteem	Neither poor nor good	Neither satisfied nor dissatisfied	50.00	50.00	50.00	50.00	3.00	Good	5-8hrs
1308.00	Male	24.00	21.88	31.15	Science and Information Technology	2nd year	Home	Service	Does not work	Prefer not to say	2.00	22-24	Normal	>=31	>=51,000 BDT	<=20000 BDT	21.00	Low self-esteem	Good	Very satisfied	100.00	100.00	100.00	100.00	5.00	Good	<4hrs
1309.00	Male	24.00	25.39	32.37	Science and Information Technology	2nd year	Hostel	Service	Part time	Regular	2.00	22-24	Overweight	>=31	<=50,000 BDT	<=20000 BDT	15.00	Low self-esteem	Very good	Very satisfied	71.43	87.50	83.33	59.38	9.00	Poor	<4hrs
1310.00	Female	21.00	20.12	32.27	Humanities and Social Sciences	2nd year	Hostel	Service	Does not work	Prefer not to say	4.00	<=21	Normal	>=31	<=50,000 BDT	<=20000 BDT	25.00	Low self-esteem	Neither poor nor good	Neither satisfied nor dissatisfied	67.86	70.83	66.67	62.50	9.00	Poor	5-8hrs
1311.00	Female	21.00	21.56	28.71	Humanities and Social Sciences	2nd year	Hostel	Business	Does not work	Regular	4.00	<=21	Normal	<=30 inch	>=51,000 BDT	<=20000 BDT	17.00	Low self-esteem	Neither poor nor good	Satisfied	82.14	79.17	75.00	87.50	14.00	Poor	5-8hrs
1312.00	Female	22.00	19.24	32.52	Humanities and Social Sciences	2nd year	Home	Service	Does not work	Regular	2.00	22-24	Normal	>=31	>=51,000 BDT	<=20000 BDT	35.00	High self-esteem	Neither poor nor good	Satisfied	82.14	70.83	75.00	93.75	11.00	Poor	5-8hrs
1313.00	Female	21.00	17.46	28.28	Humanities and Social Sciences	2nd year	Hostel	Business	Does not work	Irregular	4.00	<=21	Underweight	<=30 inch	<=50,000 BDT	<=20000 BDT	21.00	Low self-esteem	Neither poor nor good	Satisfied	75.00	66.67	58.33	50.00	10.00	Poor	<4hrs
1314.00	Female	22.00	17.02	32.79	Humanities and Social Sciences	2nd year	University Hall	Professional	Does not work	Regular	4.00	22-24	Underweight	>=31	<=50,000 BDT	<=20000 BDT	38.00	High self-esteem	Very poor	Very dissatisfied	25.00	8.33	50.00	18.75	10.00	Poor	5-8hrs
1315.00	Male	22.00	19.90	29.05	Science and Information Technology	2nd year	Home	Professional	Does not work	Regular	3.00	22-24	Normal	<=30 inch	<=50,000 BDT	<=20000 BDT	16.00	Low self-esteem	Good	Very satisfied	78.57	87.50	58.33	62.50	5.00	Good	5-8hrs
1316.00	Female	23.00	21.12	31.53	Humanities and Social Sciences	Final year	Home	Business	Full time	Regular	4.00	22-24	Normal	>=31	<=50,000 BDT	<=20000 BDT	23.00	Low self-esteem	Neither poor nor good	Satisfied	60.71	58.33	66.67	71.88	9.00	Poor	>8hrs
1317.00	Female	24.00	27.86	31.72	Humanities and Social Sciences	Final year	Hostel	Service	Full time	Irregular	4.00	22-24	Overweight	>=31	>=51,000 BDT	<=20000 BDT	26.00	Moderate self-esteem	Neither poor nor good	Very dissatisfied	14.29	20.83	58.33	40.63	15.00	Poor	5-8hrs
1318.00	Female	22.00	18.40	31.97	Humanities and Social Sciences	Final year	Home	Service	Does not work	Irregular	1.00	22-24	Underweight	>=31	<=50,000 BDT	<=20000 BDT	21.00	Low self-esteem	Good	Satisfied	64.29	66.67	41.67	68.75	5.00	Good	5-8hrs
1319.00	Female	20.00	13.44	23.08	Humanities and Social Sciences	2nd year	Home	Service	Does not work	Prefer not to say	3.00	<=21	Underweight	<=30 inch	<=50,000 BDT	<=20000 BDT	23.00	Low self-esteem	Good	Satisfied	71.43	62.50	58.33	62.50	13.00	Poor	5-8hrs
1320.00	Female	22.00	17.81	31.21	Humanities and Social Sciences	2nd year	University Hall	Business	Does not work	Irregular	4.00	22-24	Underweight	>=31	<=50,000 BDT	<=20000 BDT	19.00	Low self-esteem	Neither poor nor good	Neither satisfied nor dissatisfied	46.43	45.83	50.00	43.75	3.00	Good	<4hrs
1321.00	Female	19.00	20.59	31.53	Humanities and Social Sciences	1st year	Home	Business	Part time	Regular	2.00	<=21	Normal	>=31	>=51,000 BDT	<=20000 BDT	25.00	Low self-esteem	Neither poor nor good	Satisfied	46.43	54.17	58.33	62.50	4.00	Good	<4hrs
1322.00	Female	20.00	26.51	31.55	Humanities and Social Sciences	3rd year	Home	Professional	Does not work	Regular	4.00	<=21	Overweight	>=31	<=50,000 BDT	<=20000 BDT	25.00	Low self-esteem	Very poor	Dissatisfied	50.00	58.33	75.00	56.25	11.00	Poor	<4hrs
1323.00	Male	25.00	33.13	31.92	Science and Information Technology	1st year	Home	Professional	Does not work	Regular	3.00	>=25	Obese	>=31	<=50,000 BDT	<=20000 BDT	25.00	Low self-esteem	Neither poor nor good	Satisfied	78.57	75.00	50.00	75.00	5.00	Good	<4hrs
1324.00	Male	20.00	28.84	38.88	Science and Information Technology	1st year	Hostel	Business	Does not work	Prefer not to say	3.00	<=21	Overweight	>=31	>=51,000 BDT	<=20000 BDT	21.00	Low self-esteem	Good	Neither satisfied nor dissatisfied	57.14	50.00	83.33	53.13	5.00	Good	5-8hrs
1325.00	Male	20.00	23.34	31.8	Science and Information Technology	1st year	Hostel	Service	Does not work	Irregular	3.00	<=21	Normal	>=31	<=50,000 BDT	<=20000 BDT	21.00	Low self-esteem	Good	Satisfied	78.57	45.83	75.00	59.38	9.00	Poor	>8hrs
1326.00	Female	22.00	23.09	31.58	Humanities and Social Sciences	1st year	Home	Business	Does not work	Regular	4.00	22-24	Normal	>=31	>=51,000 BDT	<=20000 BDT	32.00	High self-esteem	Neither poor nor good	Very dissatisfied	57.14	41.67	41.67	59.38	13.00	Poor	5-8hrs
1327.00	Female	21.00	16.13	31.72	Humanities and Social Sciences	1st year	Home	Service	Does not work	Regular	1.00	<=21	Underweight	>=31	>=51,000 BDT	<=20000 BDT	25.00	Low self-esteem	Good	Neither satisfied nor dissatisfied	64.29	58.33	75.00	62.50	4.00	Good	5-8hrs
1328.00	Female	23.00	21.46	38.89	Humanities and Social Sciences	2nd year	Hostel	Business	Does not work	Regular	4.00	22-24	Normal	>=31	>=51,000 BDT	<=20000 BDT	26.00	Moderate self-esteem	Neither poor nor good	Satisfied	60.71	50.00	75.00	68.75	10.00	Poor	5-8hrs
1329.00	Female	22.00	23.88	31.9	Humanities and Social Sciences	3rd year	University Hall	Professional	Full time	Prefer not to say	2.00	22-24	Normal	>=31	>=51,000 BDT	<=20000 BDT	16.00	Low self-esteem	Good	Neither satisfied nor dissatisfied	78.57	75.00	58.33	75.00	9.00	Poor	5-8hrs
1330.00	Female	23.00	23.88	31.55	Humanities and Social Sciences	3rd year	Home	Business	Does not work	Prefer not to say	3.00	22-24	Normal	>=31	>=51,000 BDT	<=20000 BDT	22.00	Low self-esteem	Good	Neither satisfied nor dissatisfied	64.29	70.83	83.33	68.75	13.00	Poor	5-8hrs
1331.00	Female	22.00	20.25	34.13	Humanities and Social Sciences	3rd year	Home	Business	Does not work	Regular	3.00	22-24	Normal	>=31	<=50,000 BDT	<=20000 BDT	28.00	Moderate self-esteem	Neither poor nor good	Neither satisfied nor dissatisfied	60.71	37.50	41.67	37.50	10.00	Poor	<4hrs
1332.00	Female	23.00	23.09	31.84	Humanities and Social Sciences	3rd year	Home	Business	Does not work	Prefer not to say	2.00	22-24	Normal	>=31	>=51,000 BDT	<=20000 BDT	26.00	Moderate self-esteem	Neither poor nor good	Neither satisfied nor dissatisfied	39.29	66.67	58.33	53.13	19.00	Poor	<4hrs
1333.00	Female	21.00	18.15	27.5	Humanities and Social Sciences	3rd year	Hostel	Service	Does not work	Regular	1.00	<=21	Underweight	<=30 inch	>=51,000 BDT	<=20000 BDT	13.00	Low self-esteem	Good	Satisfied	82.14	87.50	100.00	81.25	3.00	Good	<4hrs
1334.00	Female	21.00	25.62	31.05	Humanities and Social Sciences	3rd year	Hostel	Business	Does not work	Regular	2.00	<=21	Overweight	>=31	<=50,000 BDT	<=20000 BDT	22.00	Low self-esteem	Very good	Dissatisfied	67.86	54.17	66.67	59.38	4.00	Good	<4hrs
1335.00	Female	22.00	23.09	31.93	Humanities and Social Sciences	3rd year	University Hall	Service	Does not work	Irregular	4.00	22-24	Normal	>=31	>=51,000 BDT	<=20000 BDT	21.00	Low self-esteem	Good	Neither satisfied nor dissatisfied	57.14	62.50	58.33	62.50	5.00	Good	5-8hrs
1336.00	Female	22.00	19.93	31.9	Humanities and Social Sciences	3rd year	Home	Service	Full time	Regular	1.00	22-24	Normal	>=31	>=51,000 BDT	<=20000 BDT	10.00	Low self-esteem	Very good	Very satisfied	89.29	95.83	83.33	87.50	5.00	Good	5-8hrs
1337.00	Female	22.00	30.89	31.59	Humanities and Social Sciences	3rd year	Home	Service	Does not work	Regular	4.00	22-24	Obese	>=31	>=51,000 BDT	<=20000 BDT	24.00	Low self-esteem	Neither poor nor good	Dissatisfied	50.00	54.17	58.33	59.38	5.00	Good	5-8hrs
1338.00	Female	22.00	15.87	31.52	Humanities and Social Sciences	3rd year	Hostel	Other jobs	Full time	Irregular	4.00	22-24	Underweight	>=31	<=50,000 BDT	<=20000 BDT	25.00	Low self-esteem	Good	Neither satisfied nor dissatisfied	64.29	54.17	66.67	53.13	11.00	Poor	5-8hrs
1339.00	Female	23.00	15.90	27.94	Humanities and Social Sciences	3rd year	University Hall	Service	Does not work	Regular	3.00	22-24	Underweight	<=30 inch	>=51,000 BDT	<=20000 BDT	21.00	Low self-esteem	Good	Satisfied	64.29	66.67	75.00	68.75	12.00	Poor	5-8hrs
1340.00	Male	24.00	19.93	30.22	Science and Information Technology	3rd year	Home	Business	Does not work	Irregular	3.00	22-24	Normal	<=30 inch	>=51,000 BDT	>=21000 BDT	11.00	Low self-esteem	Good	Satisfied	82.14	83.33	91.67	53.13	9.00	Poor	5-8hrs
1341.00	Female	22.00	24.53	31.6	Humanities and Social Sciences	3rd year	Home	Service	Does not work	Irregular	4.00	22-24	Normal	>=31	>=51,000 BDT	<=20000 BDT	15.00	Low self-esteem	Good	Satisfied	78.57	87.50	75.00	43.75	4.00	Good	<4hrs
1342.00	Female	22.00	22.15	31.94	Humanities and Social Sciences	3rd year	Hostel	Professional	Does not work	Regular	3.00	22-24	Normal	>=31	>=51,000 BDT	>=21000 BDT	15.00	Low self-esteem	Good	Neither satisfied nor dissatisfied	78.57	75.00	66.67	78.13	12.00	Poor	5-8hrs
1343.00	Female	21.00	17.85	31.18	Humanities and Social Sciences	2nd year	Hostel	Professional	Full time	Regular	2.00	<=21	Underweight	>=31	>=51,000 BDT	<=20000 BDT	19.00	Low self-esteem	Good	Satisfied	67.86	70.83	75.00	68.75	10.00	Poor	<4hrs
1344.00	Female	23.00	22.77	34.05	Humanities and Social Sciences	Final year	Home	Service	Does not work	Prefer not to say	3.00	22-24	Normal	>=31	>=51,000 BDT	<=20000 BDT	22.00	Low self-esteem	Good	Neither satisfied nor dissatisfied	53.57	75.00	75.00	56.25	4.00	Good	5-8hrs
1345.00	Female	22.00	23.13	31.99	Humanities and Social Sciences	3rd year	Hostel	Business	Does not work	Regular	3.00	22-24	Normal	>=31	>=51,000 BDT	<=20000 BDT	22.00	Low self-esteem	Good	Satisfied	64.29	70.83	66.67	59.38	4.00	Good	5-8hrs
1346.00	Male	25.00	31.84	31.23	Science and Information Technology	3rd year	University Hall	Service	Does not work	Irregular	1.00	>=25	Obese	>=31	<=50,000 BDT	<=20000 BDT	25.00	Low self-esteem	Very good	Very satisfied	60.71	70.83	66.67	81.25	15.00	Poor	>8hrs
1347.00	Male	25.00	17.23	32.22	Science and Information Technology	3rd year	Hostel	Business	Does not work	Regular	1.00	>=25	Underweight	>=31	<=50,000 BDT	<=20000 BDT	26.00	Moderate self-esteem	Good	Satisfied	60.71	62.50	75.00	75.00	14.00	Poor	<4hrs
1348.00	Male	22.00	27.86	31.82	Science and Information Technology	3rd year	Home	No response	Full time	Prefer not to say	3.00	22-24	Overweight	>=31	>=51,000 BDT	<=20000 BDT	22.00	Low self-esteem	Neither poor nor good	Dissatisfied	64.29	50.00	50.00	46.88	9.00	Poor	5-8hrs
1349.00	Female	21.00	20.67	31.49	Humanities and Social Sciences	2nd year	University Hall	Business	Does not work	Irregular	4.00	<=21	Normal	>=31	>=51,000 BDT	<=20000 BDT	23.00	Low self-esteem	Good	Neither satisfied nor dissatisfied	78.57	70.83	91.67	71.88	11.00	Poor	5-8hrs
1350.00	Female	23.00	21.04	31.52	Humanities and Social Sciences	2nd year	Home	Service	Does not work	Regular	4.00	22-24	Normal	>=31	>=51,000 BDT	<=20000 BDT	16.00	Low self-esteem	Very good	Very satisfied	53.57	54.17	75.00	78.13	10.00	Poor	<4hrs
1351.00	Female	24.00	17.92	31.08	Humanities and Social Sciences	3rd year	Hostel	Business	Does not work	Regular	4.00	22-24	Underweight	>=31	>=51,000 BDT	<=20000 BDT	25.00	Low self-esteem	Good	Satisfied	78.57	79.17	66.67	43.75	11.00	Poor	>8hrs
1352.00	Female	23.00	22.15	32.7	Humanities and Social Sciences	3rd year	University Hall	Service	Does not work	Irregular	4.00	22-24	Normal	>=31	>=51,000 BDT	<=20000 BDT	12.00	Low self-esteem	Good	Satisfied	82.14	83.33	100.00	68.75	3.00	Good	5-8hrs
1353.00	Female	24.00	29.51	40.59	Humanities and Social Sciences	3rd year	University Hall	Service	Does not work	Regular	2.00	22-24	Overweight	>=31	<=50,000 BDT	<=20000 BDT	30.00	High self-esteem	Good	Dissatisfied	64.29	50.00	58.33	65.63	10.00	Poor	5-8hrs
1354.00	Female	23.00	22.77	31.61	Humanities and Social Sciences	2nd year	Home	Business	Does not work	Prefer not to say	3.00	22-24	Normal	>=31	>=51,000 BDT	<=20000 BDT	16.00	Low self-esteem	Very good	Very satisfied	67.86	79.17	83.33	100.00	11.00	Poor	5-8hrs
1355.00	Female	23.00	20.69	31.1	Humanities and Social Sciences	3rd year	University Hall	Professional	Does not work	Regular	4.00	22-24	Normal	>=31	>=51,000 BDT	<=20000 BDT	11.00	Low self-esteem	Very good	Very satisfied	92.86	87.50	100.00	93.75	4.00	Good	>8hrs
1356.00	Female	21.00	16.48	31.02	Humanities and Social Sciences	1st year	Hostel	Service	Does not work	Regular	4.00	<=21	Underweight	>=31	>=51,000 BDT	<=20000 BDT	23.00	Low self-esteem	Good	Satisfied	92.86	66.67	91.67	71.88	3.00	Good	<4hrs
1357.00	Female	23.00	24.28	31.58	Humanities and Social Sciences	2nd year	Home	Service	Does not work	Prefer not to say	4.00	22-24	Normal	>=31	>=51,000 BDT	<=20000 BDT	18.00	Low self-esteem	Neither poor nor good	Neither satisfied nor dissatisfied	46.43	50.00	75.00	59.38	3.00	Good	5-8hrs
1358.00	Female	23.00	17.35	31.1	Humanities and Social Sciences	2nd year	Hostel	Business	Does not work	Prefer not to say	4.00	22-24	Underweight	>=31	>=51,000 BDT	<=20000 BDT	17.00	Low self-esteem	Neither poor nor good	Neither satisfied nor dissatisfied	57.14	54.17	66.67	50.00	4.00	Good	<4hrs
1359.00	Female	23.00	17.85	30.62	Humanities and Social Sciences	2nd year	Hostel	Service	Does not work	Irregular	3.00	22-24	Underweight	<=30 inch	>=51,000 BDT	<=20000 BDT	23.00	Low self-esteem	Good	Satisfied	60.71	66.67	75.00	71.88	12.00	Poor	5-8hrs
1360.00	Female	22.00	19.16	31.14	Humanities and Social Sciences	2nd year	Home	Service	Does not work	Regular	4.00	22-24	Normal	>=31	>=51,000 BDT	<=20000 BDT	19.00	Low self-esteem	Good	Satisfied	64.29	70.83	50.00	75.00	4.00	Good	<4hrs
1361.00	Female	23.00	26.39	31.42	Humanities and Social Sciences	2nd year	Home	Professional	Does not work	Prefer not to say	4.00	22-24	Overweight	>=31	>=51,000 BDT	<=20000 BDT	20.00	Low self-esteem	Very good	Very satisfied	75.00	66.67	83.33	75.00	4.00	Good	<4hrs
1362.00	Male	23.00	23.88	30.84	Science and Information Technology	Final year	Hostel	Business	Does not work	Regular	4.00	22-24	Normal	<=30 inch	>=51,000 BDT	<=20000 BDT	25.00	Low self-esteem	Good	Satisfied	53.57	58.33	50.00	59.38	10.00	Poor	>8hrs
1363.00	Female	20.00	20.69	31.76	Humanities and Social Sciences	3rd year	Home	Service	Does not work	Prefer not to say	4.00	<=21	Normal	>=31	<=50,000 BDT	<=20000 BDT	26.00	Moderate self-esteem	Good	Satisfied	64.29	54.17	83.33	65.63	13.00	Poor	<4hrs
1364.00	Female	23.00	22.99	31.78	Humanities and Social Sciences	3rd year	Home	Business	Does not work	Irregular	2.00	22-24	Normal	>=31	>=51,000 BDT	<=20000 BDT	25.00	Low self-esteem	Very good	Neither satisfied nor dissatisfied	67.86	75.00	66.67	46.88	5.00	Good	>8hrs
1365.00	Male	23.00	19.50	28.37	Science and Information Technology	3rd year	University Hall	Service	Does not work	Irregular	4.00	22-24	Normal	<=30 inch	<=50,000 BDT	>=21000 BDT	25.00	Low self-esteem	Good	Satisfied	75.00	45.83	75.00	68.75	14.00	Poor	5-8hrs
1366.00	Female	22.00	22.76	31.18	Humanities and Social Sciences	2nd year	University Hall	Service	Full time	Prefer not to say	3.00	22-24	Normal	>=31	>=51,000 BDT	<=20000 BDT	15.00	Low self-esteem	Good	Satisfied	75.00	79.17	50.00	56.25	10.00	Poor	<4hrs
1367.00	Female	22.00	14.72	31.92	Humanities and Social Sciences	2nd year	Home	Professional	Full time	Prefer not to say	4.00	22-24	Underweight	>=31	>=51,000 BDT	<=20000 BDT	16.00	Low self-esteem	Good	Satisfied	53.57	58.33	75.00	59.38	13.00	Poor	<4hrs
1368.00	Female	23.00	19.17	31.1	Humanities and Social Sciences	2nd year	Hostel	Service	Does not work	Prefer not to say	4.00	22-24	Normal	>=31	>=51,000 BDT	<=20000 BDT	22.00	Low self-esteem	Neither poor nor good	Neither satisfied nor dissatisfied	39.29	54.17	16.67	75.00	5.00	Good	<4hrs
1369.00	Female	23.00	19.91	31.22	Humanities and Social Sciences	2nd year	Home	Professional	Does not work	Irregular	4.00	22-24	Normal	>=31	>=51,000 BDT	<=20000 BDT	31.00	High self-esteem	Poor	Dissatisfied	42.86	29.17	58.33	34.38	10.00	Poor	5-8hrs
1370.00	Female	21.00	13.91	31.97	Humanities and Social Sciences	2nd year	Hostel	Business	Does not work	Irregular	4.00	<=21	Underweight	>=31	>=51,000 BDT	<=20000 BDT	29.00	Moderate self-esteem	Poor	Dissatisfied	32.14	20.83	58.33	40.63	12.00	Poor	5-8hrs
1371.00	Female	22.00	18.38	31.46	Humanities and Social Sciences	2nd year	University Hall	Business	Does not work	Prefer not to say	4.00	22-24	Underweight	>=31	>=51,000 BDT	<=20000 BDT	19.00	Low self-esteem	Poor	Dissatisfied	32.14	41.67	41.67	43.75	3.00	Good	<4hrs
1372.00	Male	21.00	30.27	38.28	Science and Information Technology	2nd year	Home	Business	Does not work	Prefer not to say	3.00	<=21	Obese	>=31	>=51,000 BDT	<=20000 BDT	25.00	Low self-esteem	Good	Neither satisfied nor dissatisfied	60.71	66.67	58.33	50.00	5.00	Good	5-8hrs
1373.00	Female	23.00	18.39	31.44	Humanities and Social Sciences	2nd year	Home	Service	Does not work	Prefer not to say	4.00	22-24	Underweight	>=31	>=51,000 BDT	<=20000 BDT	13.00	Low self-esteem	Neither poor nor good	Neither satisfied nor dissatisfied	53.57	50.00	83.33	84.38	3.00	Good	<4hrs
1374.00	Female	22.00	21.46	31.13	Humanities and Social Sciences	3rd year	University Hall	Service	Does not work	Prefer not to say	4.00	22-24	Normal	>=31	>=51,000 BDT	<=20000 BDT	15.00	Low self-esteem	Very good	Very satisfied	67.86	45.83	75.00	65.63	11.00	Poor	5-8hrs
1375.00	Female	23.00	19.52	31.45	Humanities and Social Sciences	3rd year	Home	Business	Does not work	Prefer not to say	4.00	22-24	Normal	>=31	>=51,000 BDT	<=20000 BDT	18.00	Low self-esteem	Very good	Very satisfied	60.71	75.00	75.00	90.63	5.00	Good	<4hrs
1376.00	Female	24.00	23.63	31.96	Humanities and Social Sciences	3rd year	Hostel	Service	Does not work	Regular	4.00	22-24	Normal	>=31	>=51,000 BDT	<=20000 BDT	20.00	Low self-esteem	Neither poor nor good	Satisfied	67.86	62.50	75.00	75.00	5.00	Good	5-8hrs
1377.00	Female	22.00	15.12	31.8	Humanities and Social Sciences	3rd year	University Hall	Service	Does not work	Regular	4.00	22-24	Underweight	>=31	>=51,000 BDT	<=20000 BDT	21.00	Low self-esteem	Neither poor nor good	Satisfied	67.86	62.50	75.00	78.13	10.00	Poor	<4hrs
1378.00	Female	23.00	15.33	31.63	Humanities and Social Sciences	3rd year	Hostel	Business	Does not work	Irregular	4.00	22-24	Underweight	>=31	>=51,000 BDT	<=20000 BDT	23.00	Low self-esteem	Neither poor nor good	Satisfied	42.86	58.33	66.67	62.50	10.00	Poor	5-8hrs
1379.00	Female	23.00	21.78	31.27	Humanities and Social Sciences	3rd year	Home	Service	Does not work	Prefer not to say	4.00	22-24	Normal	>=31	>=51,000 BDT	<=20000 BDT	20.00	Low self-esteem	Very good	Satisfied	71.43	83.33	75.00	90.63	12.00	Poor	<4hrs
1380.00	Female	22.00	21.49	31.94	Humanities and Social Sciences	3rd year	University Hall	Business	Does not work	Prefer not to say	4.00	22-24	Normal	>=31	>=51,000 BDT	<=20000 BDT	13.00	Low self-esteem	Neither poor nor good	Neither satisfied nor dissatisfied	28.57	50.00	50.00	46.88	4.00	Good	<4hrs
1381.00	Female	24.00	22.77	31.94	Humanities and Social Sciences	3rd year	Home	Service	Does not work	Prefer not to say	3.00	22-24	Normal	>=31	>=51,000 BDT	<=20000 BDT	17.00	Low self-esteem	Very good	Very satisfied	71.43	91.67	75.00	100.00	1.00	Good	<4hrs
1382.00	Female	24.00	28.14	31.9	Humanities and Social Sciences	3rd year	Hostel	Service	Does not work	Prefer not to say	4.00	22-24	Overweight	>=31	>=51,000 BDT	<=20000 BDT	19.00	Low self-esteem	Very good	Very satisfied	85.71	100.00	83.33	100.00	3.00	Good	5-8hrs
1383.00	Female	21.00	23.34	30.96	Humanities and Social Sciences	2nd year	Hostel	Service	Does not work	Regular	3.00	<=21	Normal	<=30 inch	<=50,000 BDT	<=20000 BDT	23.00	Low self-esteem	Neither poor nor good	Dissatisfied	67.86	58.33	83.33	53.13	10.00	Poor	5-8hrs
1384.00	Female	24.00	24.20	31.88	Humanities and Social Sciences	2nd year	Home	Service	Does not work	Regular	4.00	22-24	Normal	>=31	>=51,000 BDT	<=20000 BDT	20.00	Low self-esteem	Neither poor nor good	Neither satisfied nor dissatisfied	67.86	58.33	66.67	68.75	5.00	Good	<4hrs

1385.00	Female	22.00	24.42	31.41	Humanities and Social Sciences	2nd year	Home	Service	Part time	Prefer not to say	4.00	22-24	Normal	>=31	>=51,000 BDT	<=20000 BDT	21.00	Low self-esteem	Very good	Very satisfied	67.86	62.50	75.00	71.88	10.00	Poor	5-8hrs
1386.00	Female	22.00	28.74	40.25	Humanities and Social Sciences	2nd year	University Hall	No response	Does not work	Irregular	3.00	22-24	Overweight	>=31	<=50,000 BDT	<=20000 BDT	29.00	Moderate self-esteem	Neither poor nor good	Neither satisfied nor dissatisfied	50.00	58.33	66.67	46.88	9.00	Poor	5-8hrs
1387.00	Female	22.00	16.48	31.55	Humanities and Social Sciences	2nd year	Home	Business	Does not work	Prefer not to say	4.00	22-24	Underweight	>=31	>=51,000 BDT	<=20000 BDT	13.00	Low self-esteem	Good	Satisfied	39.29	54.17	66.67	53.13	11.00	Poor	<4hrs
1388.00	Male	21.00	23.76	32.17	Science and Information Technology	2nd year	University Hall	Professional	Full time	Irregular	4.00	<=21	Normal	>=31	<=50,000 BDT	<=20000 BDT	16.00	Low self-esteem	Good	Satisfied	85.71	91.67	83.33	46.88	4.00	Good	5-8hrs
1389.00	Female	21.00	24.12	24.11	Humanities and Social Sciences	2nd year	Home	Business	Does not work	Regular	3.00	<=21	Normal	<=30 inch	<=50,000 BDT	<=20000 BDT	17.00	Low self-esteem	Neither poor nor good	Very dissatisfied	32.14	75.00	91.67	62.50	10.00	Poor	5-8hrs
1390.00	Female	22.00	20.69	30.2	Humanities and Social Sciences	2nd year	Hostel	Service	Full time	Regular	3.00	22-24	Normal	<=30 inch	>=51,000 BDT	<=20000 BDT	21.00	Low self-esteem	Neither poor nor good	Satisfied	60.71	66.67	58.33	46.88	9.00	Poor	5-8hrs
1391.00	Female	21.00	22.15	31.29	Humanities and Social Sciences	2nd year	Hostel	Professional	Does not work	Regular	3.00	<=21	Normal	>=31	>=51,000 BDT	<=20000 BDT	19.00	Low self-esteem	Neither poor nor good	Satisfied	67.86	58.33	58.33	43.75	4.00	Good	5-8hrs
1392.00	Female	23.00	21.81	31.22	Humanities and Social Sciences	2nd year	Home	Service	Does not work	Irregular	4.00	22-24	Normal	>=31	>=51,000 BDT	<=20000 BDT	18.00	Low self-esteem	Neither poor nor good	Neither satisfied nor dissatisfied	75.00	62.50	58.33	75.00	10.00	Poor	>8hrs
1393.00	Female	22.00	24.00	29.1	Humanities and Social Sciences	2nd year	University Hall	Business	Full time	Prefer not to say	4.00	22-24	Normal	<=30 inch	<=50,000 BDT	<=20000 BDT	25.00	Low self-esteem	Neither poor nor good	Dissatisfied	75.00	62.50	66.67	46.88	4.00	Good	5-8hrs
1394.00	Female	22.00	18.39	28.65	Humanities and Social Sciences	2nd year	Home	No response	Does not work	Prefer not to say	4.00	22-24	Underweight	<=30 inch	>=51,000 BDT	<=20000 BDT	28.00	Moderate self-esteem	Good	Neither satisfied nor dissatisfied	35.71	37.50	50.00	37.50	9.00	Poor	5-8hrs
1395.00	Female	21.00	19.20	30.18	Humanities and Social Sciences	2nd year	Home	Business	Does not work	Prefer not to say	4.00	<=21	Normal	<=30 inch	>=51,000 BDT	<=20000 BDT	30.00	High self-esteem	Neither poor nor good	Neither satisfied nor dissatisfied	50.00	41.67	66.67	43.75	12.00	Poor	5-8hrs
1396.00	Female	21.00	17.93	32.58	Humanities and Social Sciences	2nd year	Home	No response	Does not work	Prefer not to say	3.00	<=21	Underweight	>=31	>=51,000 BDT	<=20000 BDT	24.00	Low self-esteem	Good	Dissatisfied	50.00	50.00	66.67	43.75	9.00	Poor	5-8hrs
1397.00	Female	23.00	13.44	31.37	Humanities and Social Sciences	2nd year	University Hall	No response	Full time	Prefer not to say	4.00	22-24	Underweight	>=31	>=51,000 BDT	<=20000 BDT	20.00	Low self-esteem	Neither poor nor good	Dissatisfied	57.14	50.00	75.00	53.13	3.00	Good	5-8hrs
1398.00	Female	21.00	15.66	31.32	Humanities and Social Sciences	2nd year	University Hall	Business	Does not work	Regular	3.00	<=21	Underweight	>=31	>=51,000 BDT	<=20000 BDT	17.00	Low self-esteem	Good	Neither satisfied nor dissatisfied	64.29	50.00	50.00	46.88	12.00	Poor	5-8hrs
1399.00	Female	23.00	19.91	31.48	Humanities and Social Sciences	2nd year	Home	Business	Does not work	Irregular	4.00	22-24	Normal	>=31	>=51,000 BDT	<=20000 BDT	20.00	Low self-esteem	Good	Satisfied	60.71	58.33	58.33	46.88	4.00	Good	>8hrs
1400.00	Female	22.00	20.64	31.05	Humanities and Social Sciences	2nd year	Home	Business	Does not work	Prefer not to say	2.00	22-24	Normal	>=31	>=51,000 BDT	<=20000 BDT	16.00	Low self-esteem	Good	Dissatisfied	50.00	66.67	50.00	53.13	9.00	Poor	>8hrs
1401.00	Female	22.00	19.52	31.46	Humanities and Social Sciences	3rd year	Hostel	Service	Does not work	Prefer not to say	4.00	22-24	Normal	>=31	>=51,000 BDT	<=20000 BDT	14.00	Low self-esteem	Very good	Very satisfied	64.29	91.67	66.67	90.63	5.00	Good	5-8hrs
1402.00	Male	24.00	24.37	31.2	Science and Information Technology	2nd year	Hostel	No response	Does not work	Irregular	4.00	22-24	Normal	>=31	>=51,000 BDT	<=20000 BDT	23.00	Low self-esteem	Good	Satisfied	71.43	66.67	58.33	59.38	2.00	Good	5-8hrs
1403.00	Male	21.00	19.88	31.79	Science and Information Technology	1st year	Hostel	Business	Does not work	Irregular	4.00	<=21	Normal	>=31	>=51,000 BDT	<=20000 BDT	22.00	Low self-esteem	Good	Satisfied	64.29	62.50	58.33	65.63	3.00	Good	5-8hrs
1404.00	Male	24.00	23.99	31.25	Science and Information Technology	3rd year	Hostel	Service	Does not work	Irregular	4.00	22-24	Normal	>=31	>=51,000 BDT	<=20000 BDT	22.00	Low self-esteem	Neither poor nor good	Neither satisfied nor dissatisfied	46.43	62.50	58.33	71.88	4.00	Good	5-8hrs
1405.00	Male	22.00	24.03	31.16	Science and Information Technology	2nd year	Hostel	Business	Does not work	Irregular	4.00	22-24	Normal	>=31	>=51,000 BDT	<=20000 BDT	25.00	Low self-esteem	Neither poor nor good	Neither satisfied nor dissatisfied	64.29	70.83	66.67	68.75	4.00	Good	5-8hrs
1406.00	Male	23.00	23.19	31.83	Science and Information Technology	3rd year	Hostel	Professional	Does not work	Irregular	4.00	22-24	Normal	>=31	>=51,000 BDT	<=20000 BDT	26.00	Moderate self-esteem	Neither poor nor good	Neither satisfied nor dissatisfied	67.86	62.50	75.00	59.38	5.00	Good	5-8hrs
1407.00	Male	24.00	23.06	31.71	Science and Information Technology	2nd year	Hostel	Business	Does not work	Regular	3.00	22-24	Normal	>=31	>=51,000 BDT	<=20000 BDT	25.00	Low self-esteem	Poor	Satisfied	75.00	45.83	50.00	62.50	4.00	Good	5-8hrs
1408.00	Male	22.00	19.51	31.67	Science and Information Technology	2nd year	Hostel	Service	Does not work	Regular	4.00	22-24	Normal	>=31	>=51,000 BDT	<=20000 BDT	27.00	Moderate self-esteem	Good	Satisfied	78.57	79.17	66.67	68.75	5.00	Good	5-8hrs
1409.00	Male	23.00	21.28	31.65	Science and Information Technology	2nd year	Hostel	Service	Does not work	Irregular	4.00	22-24	Normal	>=31	>=51,000 BDT	<=20000 BDT	22.00	Low self-esteem	Good	Satisfied	53.57	75.00	75.00	65.63	5.00	Good	5-8hrs
1410.00	Male	22.00	21.28	31.52	Science and Information Technology	1st year	Hostel	Service	Does not work	Irregular	3.00	22-24	Normal	>=31	>=51,000 BDT	<=20000 BDT	21.00	Low self-esteem	Good	Satisfied	71.43	66.67	66.67	68.75	4.00	Good	5-8hrs
1411.00	Male	24.00	24.83	31.11	Science and Information Technology	3rd year	Hostel	Service	Does not work	Irregular	3.00	22-24	Normal	>=31	>=51,000 BDT	<=20000 BDT	22.00	Low self-esteem	Good	Neither satisfied nor dissatisfied	60.71	54.17	66.67	71.88	3.00	Good	5-8hrs
1412.00	Male	28.00	23.77	31.22	Science and Information Technology	3rd year	Hostel	Service	Does not work	Irregular	4.00	>=25	Normal	>=31	>=51,000 BDT	<=20000 BDT	26.00	Moderate self-esteem	Good	Satisfied	71.43	70.83	66.67	71.88	3.00	Good	5-8hrs
1413.00	Male	23.00	21.28	31.55	Science and Information Technology	2nd year	Hostel	Professional	Does not work	Irregular	4.00	22-24	Normal	>=31	>=51,000 BDT	<=20000 BDT	25.00	Low self-esteem	Good	Neither satisfied nor dissatisfied	50.00	66.67	50.00	59.38	3.00	Good	5-8hrs
1414.00	Male	25.00	23.41	31.72	Science and Information Technology	3rd year	Hostel	Business	Does not work	Irregular	4.00	>=25	Normal	>=31	>=51,000 BDT	<=20000 BDT	22.00	Low self-esteem	Good	Satisfied	71.43	66.67	58.33	71.88	1.00	Good	5-8hrs
1415.00	Male	23.00	20.57	31.64	Science and Information Technology	2nd year	Hostel	Service	Does not work	Irregular	4.00	22-24	Normal	>=31	>=51,000 BDT	<=20000 BDT	20.00	Low self-esteem	Good	Neither satisfied nor dissatisfied	64.29	62.50	66.67	75.00	4.00	Good	5-8hrs
1416.00	Male	21.00	21.28	31.25	Science and Information Technology	1st year	Hostel	Service	Does not work	Irregular	3.00	<=21	Normal	>=31	>=51,000 BDT	<=20000 BDT	21.00	Low self-esteem	Neither poor nor good	Neither satisfied nor dissatisfied	71.43	70.83	66.67	75.00	4.00	Good	5-8hrs
1417.00	Male	24.00	23.41	31.71	Science and Information Technology	3rd year	Hostel	Business	Does not work	Irregular	4.00	22-24	Normal	>=31	>=51,000 BDT	<=20000 BDT	20.00	Low self-esteem	Good	Satisfied	64.29	75.00	66.67	75.00	3.00	Good	5-8hrs
